# Supplementary material for: Topology-aware pathway analysis of spatial transcriptomics
Source: PeerJ. 2025 Aug 14;13:e19729. doi: 10.7717/peerj.19729 (PMC12358110; doi:10.7717/peerj.19729)
Supplement: Supplemental Information 4 [file peerj-13-19729-s004.pdf]

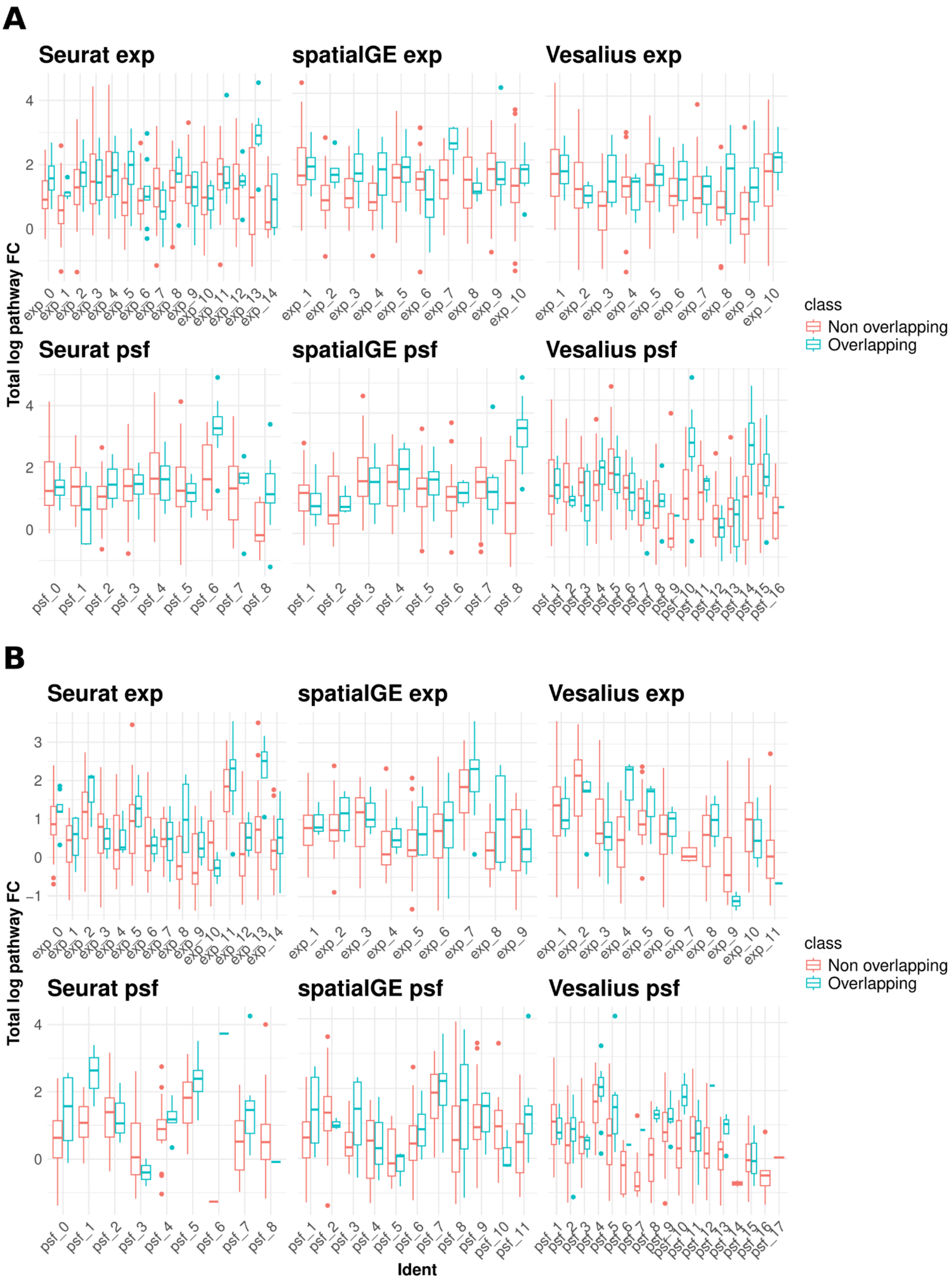

A
Seurat exp
spatialGE exp
Vesalius exp
Seurat psf
spatialGE psf
Vesalius psf
Total log pathway FC
class
Non overlapping
Overlapping
B
Seurat exp
spatialGE exp
Vesalius exp
Seurat psf
spatialGE psf
Vesalius psf
Total log pathway FC
class
Non overlapping
Overlapping
Ident

Figure S1. Total pathway activity change per cluster, calculated as the sum of absolute $\log_2$ pathway activity values, for pathways only detected by PSF (red) versus those overlapping (blue) with ORA and GSDensity. Six boxplot groups are shown for each dataset, corresponding to the three clustering methods (Seurat, Vesalius, spatialGE) applied to both gene expression and pathway activity data. Panel A represents the human melanoma dataset, and Panel B represents the mouse brain dataset. The plots show that, in nearly all clusters across both datasets and all clustering methods, the total pathway activity change is greater for pathways that overlap with other methods, compared to those uniquely identified by PSF.

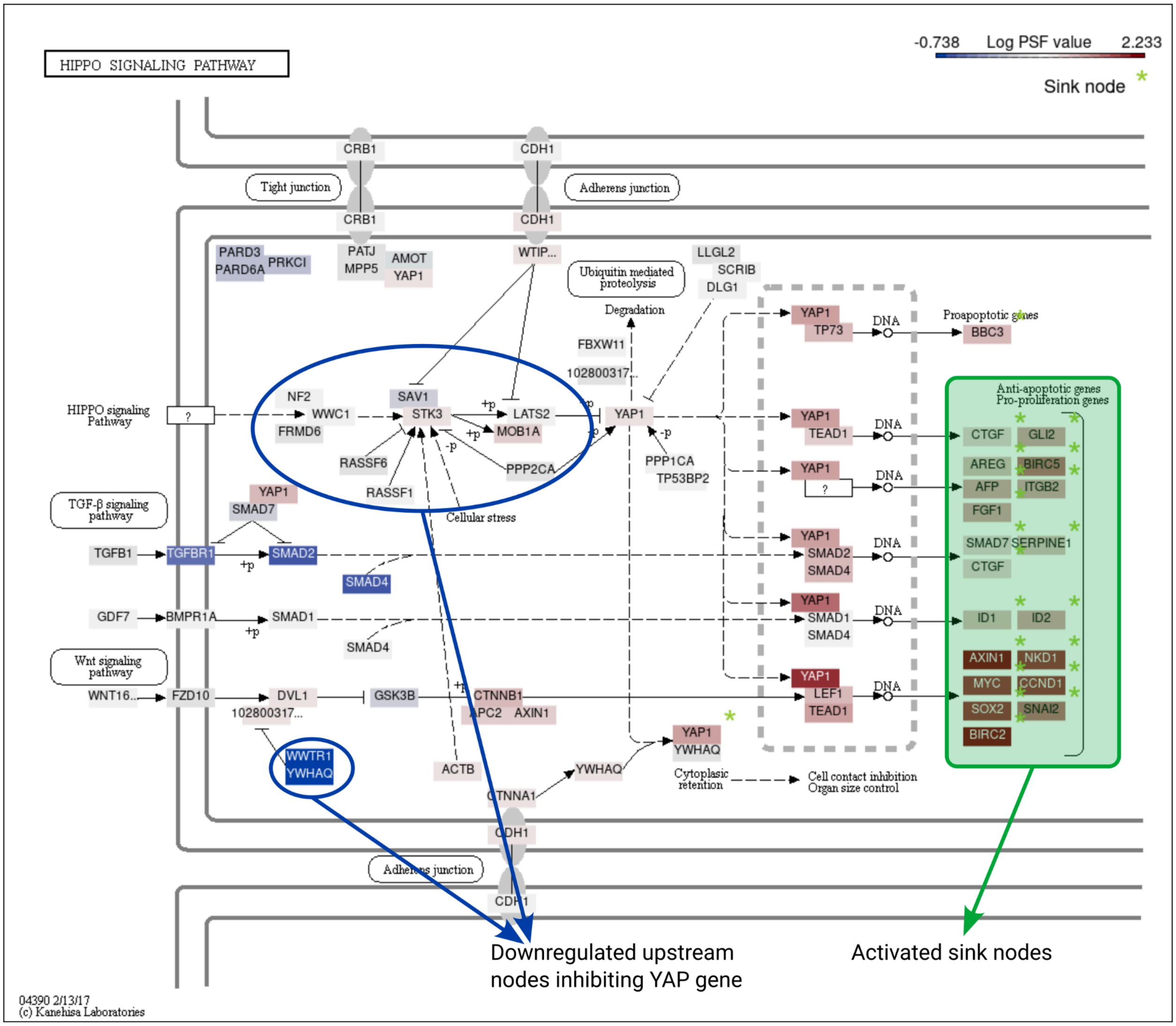

Figure S2. Pathway activity profile of the Hippo signaling pathway in PSF cluster 3 of the melanoma dataset. The color coding represents the mean log fold change PSF values, calculated by dividing the average PSF activity of cluster 3 spots by the average PSF activity of all other clusters. Colors range from white to red, indicating activation, and white to blue, indicating inhibition.

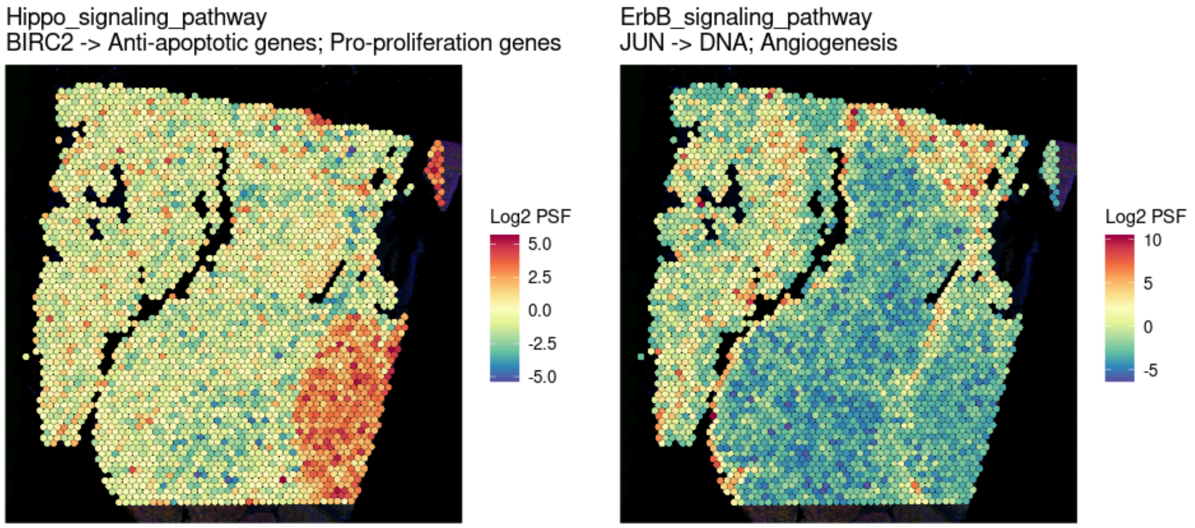

Figure S3. Visualization of PSF-derived activity values for two significantly deregulated pathways highlights the spatial distribution of pathway activity across the melanoma tissue slice. Each panel represents a single pathway branch, with the terminal (sink) node and its associated downstream biological processes indicated. Notably, activation of the BRIC2 branch of the Hippo signaling pathway linked to anti-apoptosis and proliferation is concentrated in the lower right region of the tissue, corresponding to the proliferative melanoma cell cluster identified by Schmidt et al. [ref]. Similarly, the JUN branch of the ErbB signaling pathway, associated with gene expression and angiogenesis, shows elevated activity in the upper right corner of the slice, aligning with the immune-active melanoma type 2 region described in the same study.

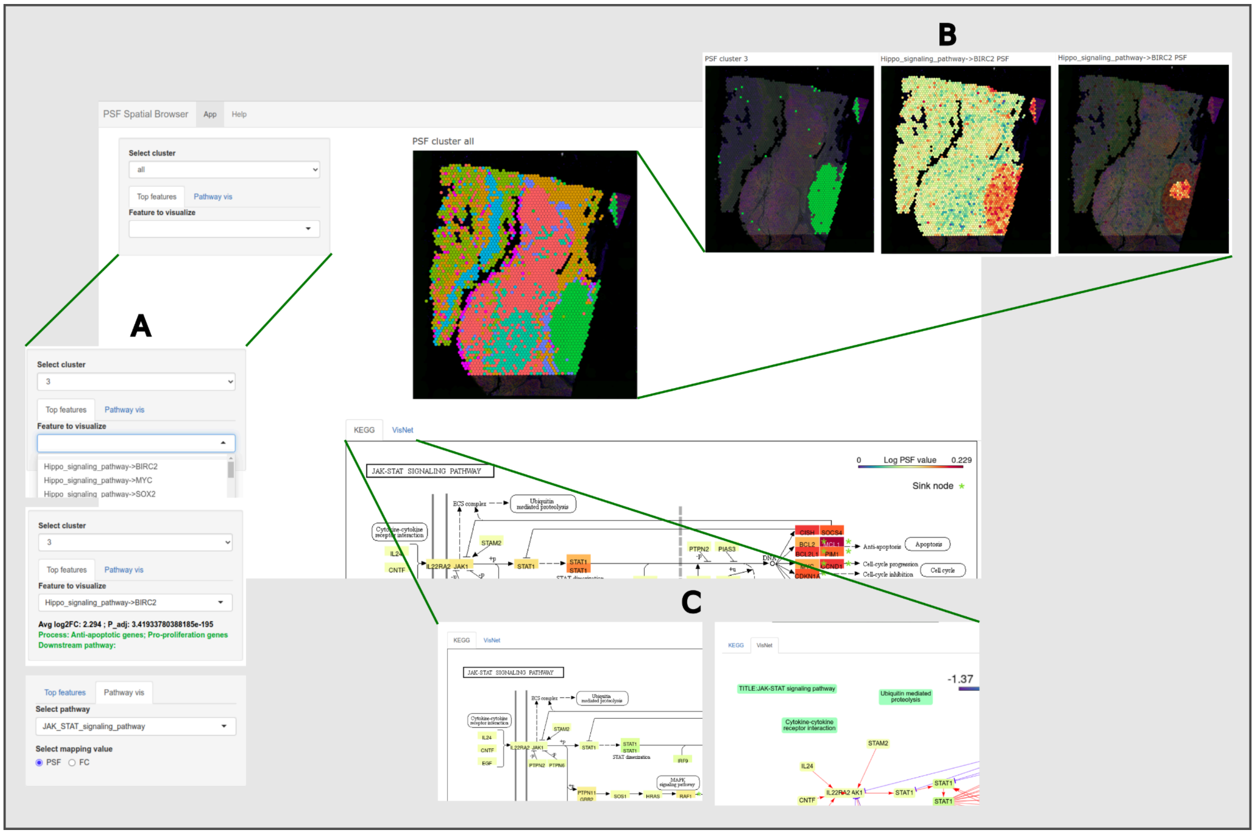

Figure S4. PSF Spatial Browser interface and visualization modules. **A.** The main control panel (top to bottom) includes: a dropdown menu to select a cluster of interest; an input panel to select top cluster-specific features; and a feature information box displaying fold change, adjusted p-value, and associated downstream biological processes for the selected pathway branch. The pathway visualization module allows users to select a specific pathway and data type (gene expression or pathway activity) to render on the pathway map. **B.** Visualization modes of the interactive spatial plot. From left to right: highlighting of a selected cluster, overlay of pathway activity or gene expression values on the spatial tissue map, and custom spot selection.
C. Pathway visualization options. On the left, a KEGG pathway image-based view allows hovering over nodes to display gene names, expression fold changes, and PSF activity scores. On the right, an interactive network-based visualization provides an alternative view of the pathway topology. In both modes, selected genes can be mapped onto the spatial tissue plot to visualize their expression or activity values.
