# Supplementary material for: Topology-aware pathway analysis of spatial transcriptomics
Source: PeerJ. 2025 Aug 14;13:e19729. doi: 10.7717/peerj.19729 (PMC12358110; doi:10.7717/peerj.19729)
Supplement: Supplemental Information 5 — For each clustering method and dataset, two pages of results are provided: the first page visualizes spatial clustering by cluster assignments, and the second page displays Venn diagrams showing the overlap of significantly deregulated pathways identified by the three functional annotation tools. [file peerj-13-19729-s005.pdf]

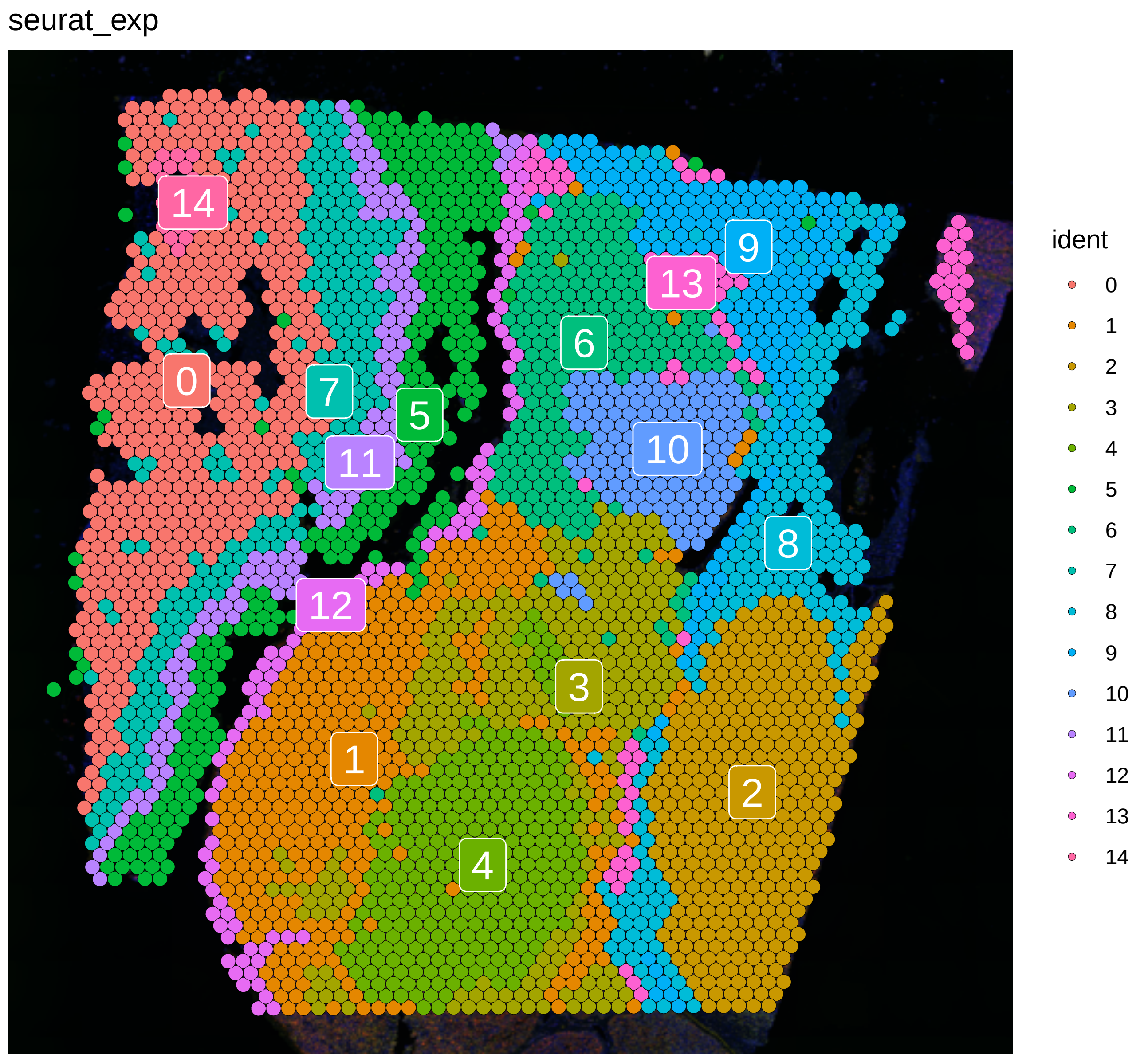
seurat_exp
14
0
7
5
11
12
6
13
9
10
8
3
1
2
4
ident
0
1
2
3
4
5
6
7
8
9
10
11
12
13
14

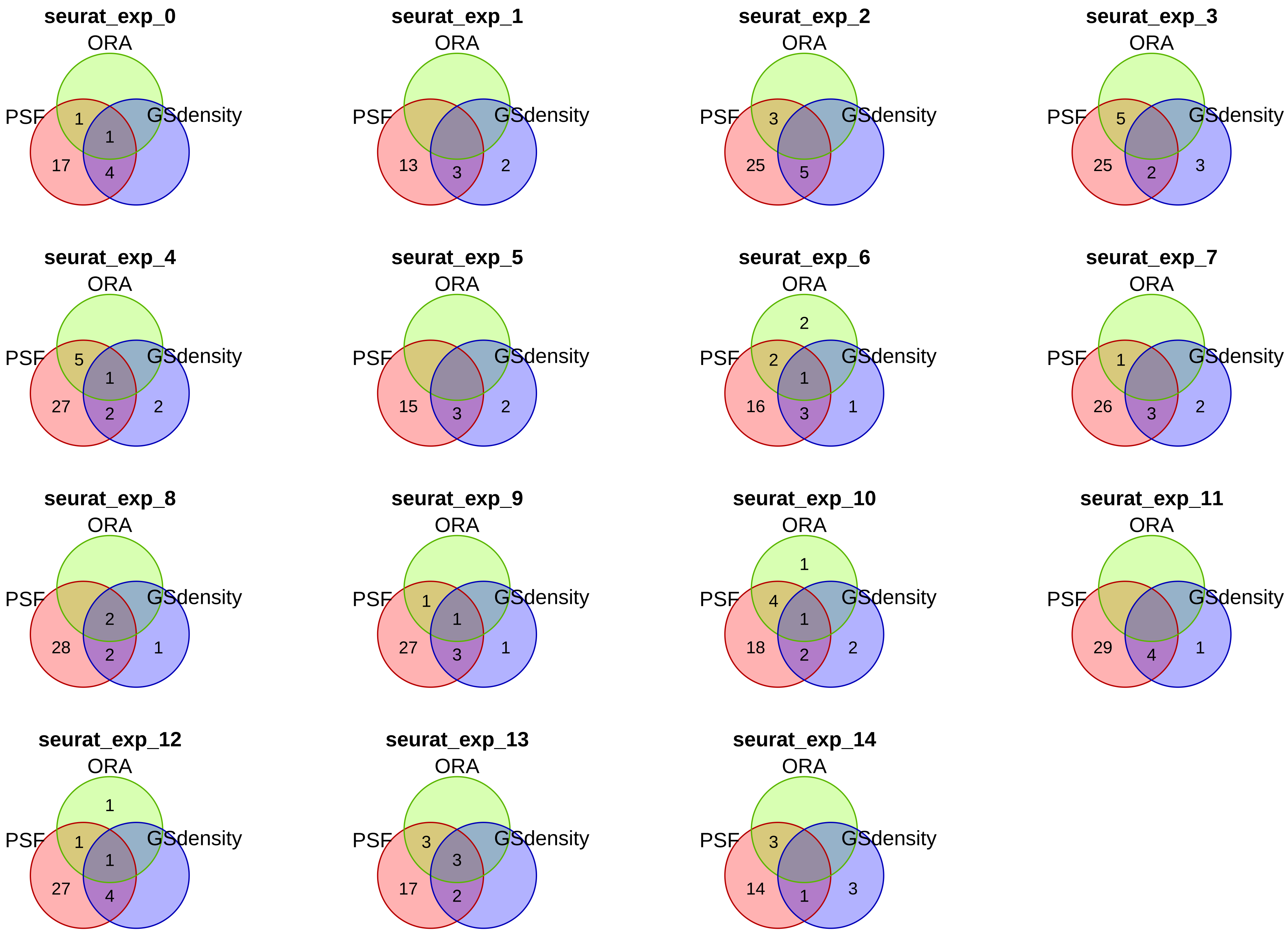
seurat_exp_0
ORA
PSF
GSdensity
1
1
17
4
seurat_exp_1
ORA
PSF
GSdensity
13
3
2
seurat_exp_2
ORA
PSF
GSdensity
3
25
5
seurat_exp_3
ORA
PSF
GSdensity
5
25
2
3
seurat_exp_4
ORA
PSF
GSdensity
5
1
27
2
2
seurat_exp_5
ORA
PSF
GSdensity
15
3
2
seurat_exp_6
ORA
PSF
GSdensity
2
2
1
16
3
1
seurat_exp_7
ORA
PSF
GSdensity
1
26
3
2
seurat_exp_8
ORA
PSF
GSdensity
2
28
2
1
seurat_exp_9
ORA
PSF
GSdensity
1
1
27
3
1
seurat_exp_10
ORA
PSF
GSdensity
1
4
1
18
2
2
seurat_exp_11
ORA
PSF
GSdensity
29
4
1
seurat_exp_12
ORA
PSF
GSdensity
1
1
1
27
4
seurat_exp_13
ORA
PSF
GSdensity
3
3
17
2
seurat_exp_14
ORA
PSF
GSdensity
3
14
1
3

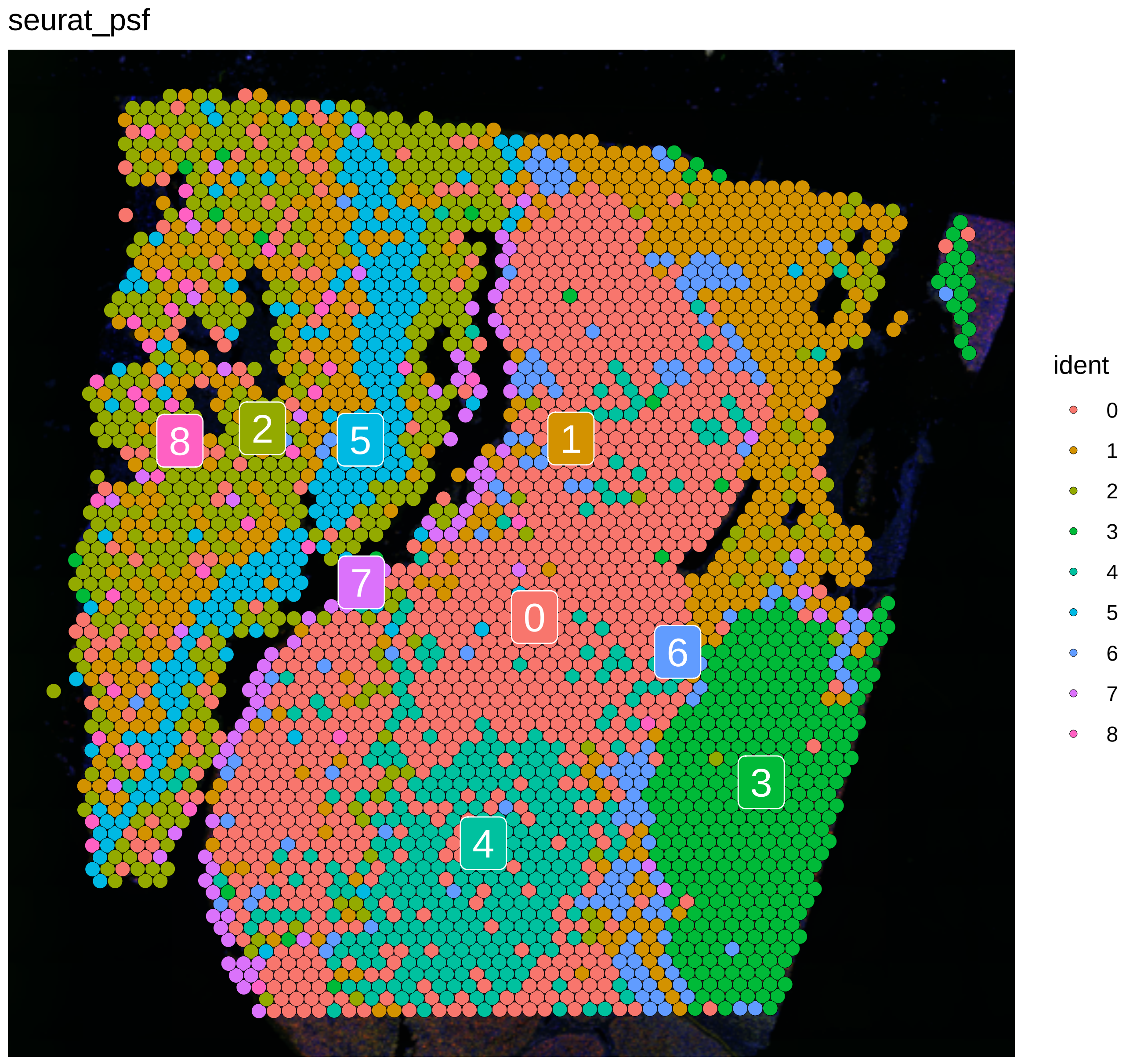
seurat_psf
ident
0
1
2
3
4
5
6
7
8

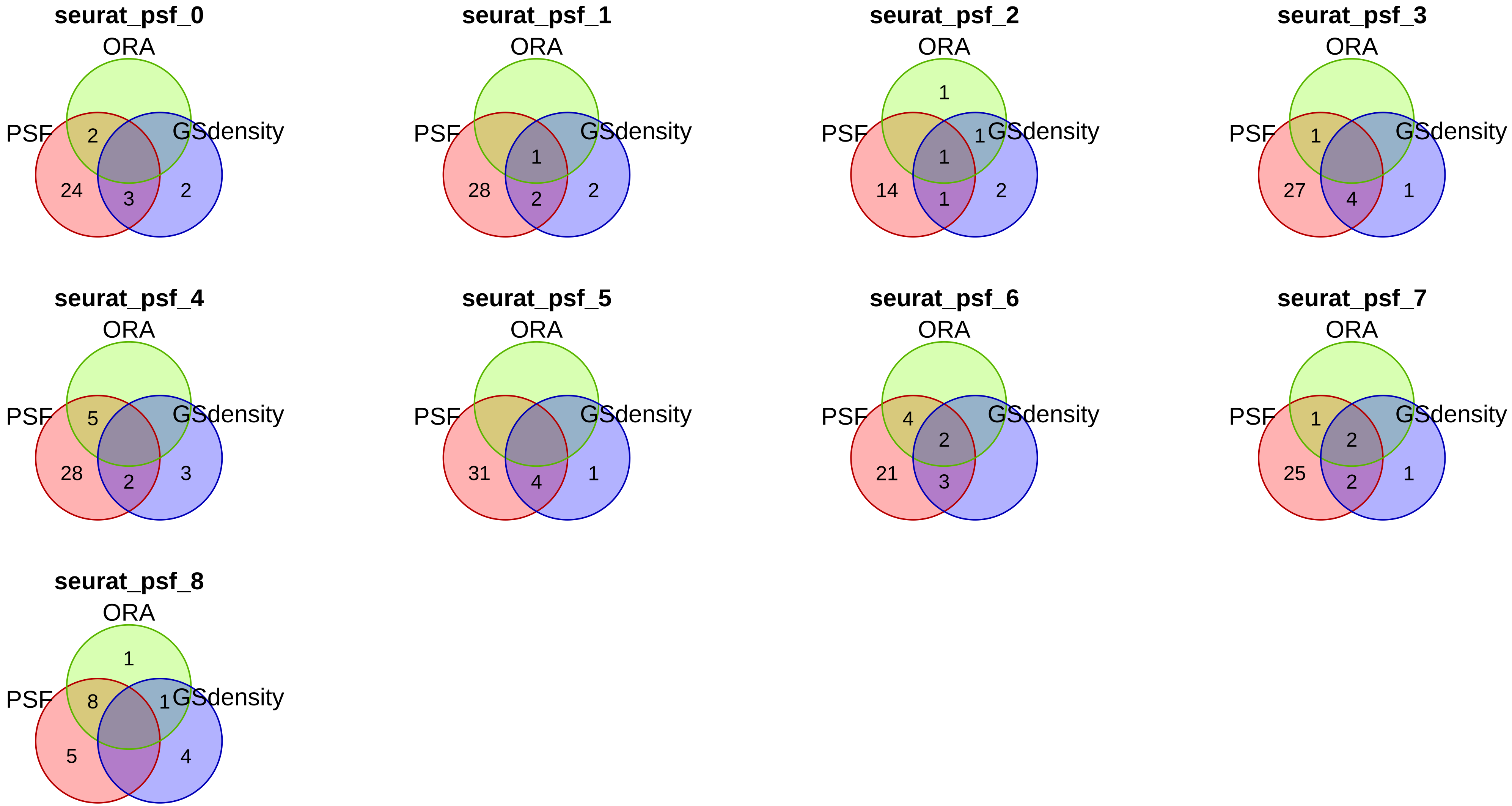
seurat_psf_0
ORA
PSF
GSdensity
2
24
3
2
seurat_psf_1
ORA
PSF
GSdensity
1
28
2
2
seurat_psf_2
ORA
PSF
GSdensity
1
1
1
14
1
2
seurat_psf_3
ORA
PSF
GSdensity
1
27
4
1
seurat_psf_4
ORA
PSF
GSdensity
5
28
2
3
seurat_psf_5
ORA
PSF
GSdensity
31
4
1
seurat_psf_6
ORA
PSF
GSdensity
4
2
21
3
seurat_psf_7
ORA
PSF
GSdensity
1
2
25
2
1
seurat_psf_8
ORA
PSF
GSdensity
1
8
1
5
4

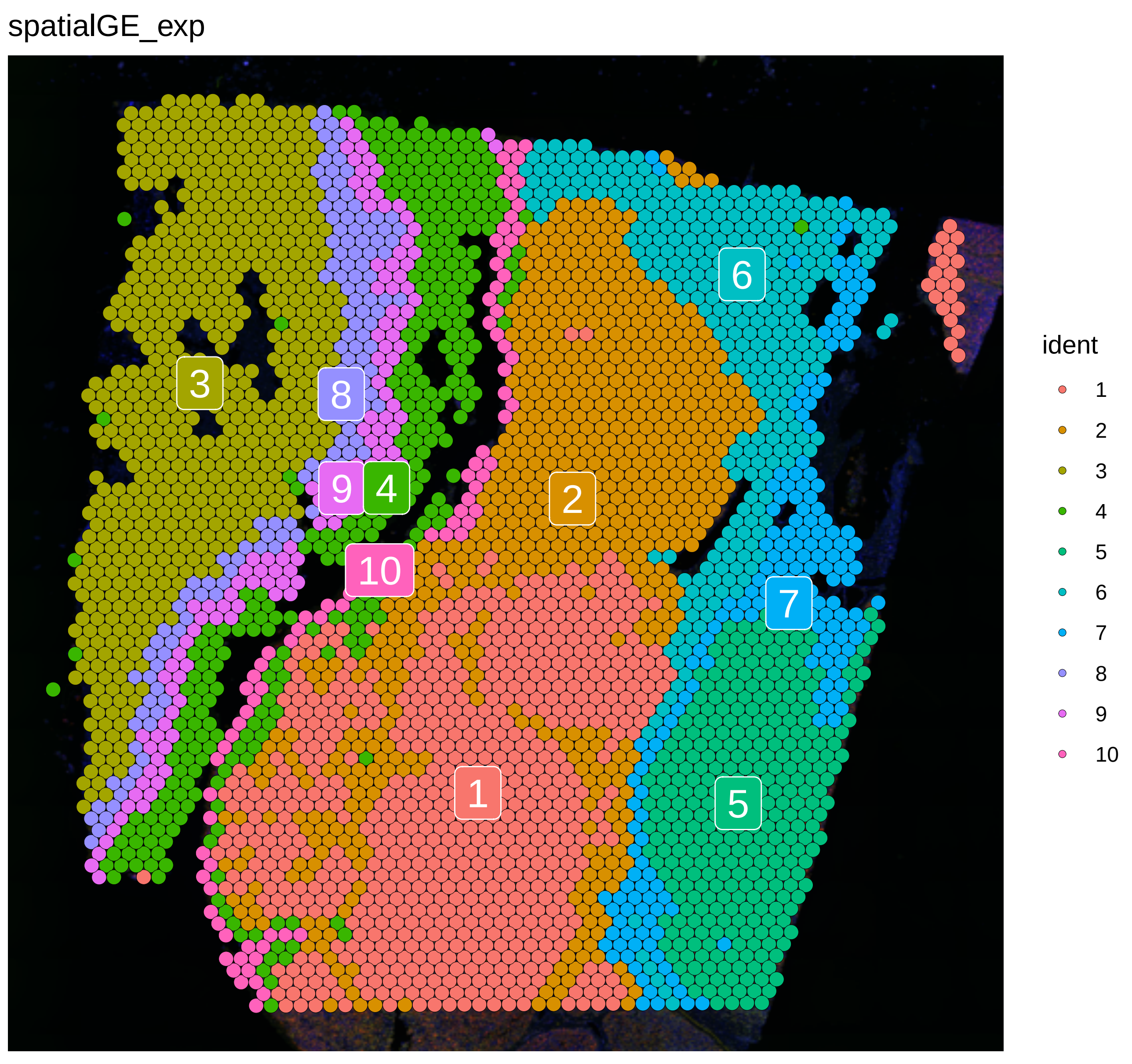

spatialGE_exp
3
8
9
4
10
2
6
7
1
5
ident
1
2
3
4
5
6
7
8
9
10

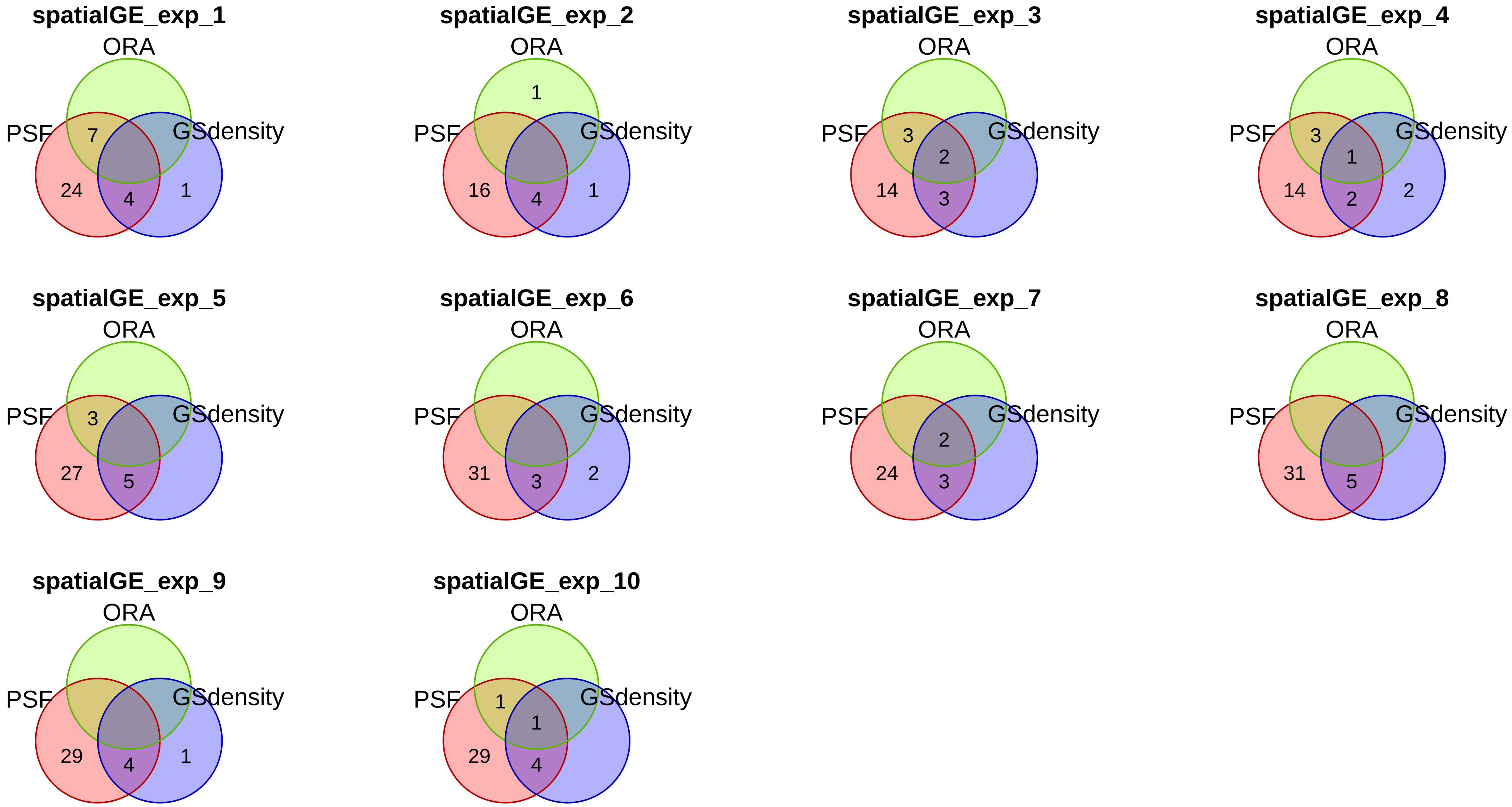
spatialGE_exp_1
ORA
PSF
GSdensity
7
24
4
1
spatialGE_exp_2
ORA
PSF
GSdensity
1
16
4
1
spatialGE_exp_3
ORA
PSF
GSdensity
3
2
14
3
spatialGE_exp_4
ORA
PSF
GSdensity
3
1
14
2
2
spatialGE_exp_5
ORA
PSF
GSdensity
3
27
5
spatialGE_exp_6
ORA
PSF
GSdensity
31
3
2
spatialGE_exp_7
ORA
PSF
GSdensity
2
24
3
spatialGE_exp_8
ORA
PSF
GSdensity
31
5
spatialGE_exp_9
ORA
PSF
GSdensity
29
4
1
spatialGE_exp_10
ORA
PSF
GSdensity
1
1
29
4

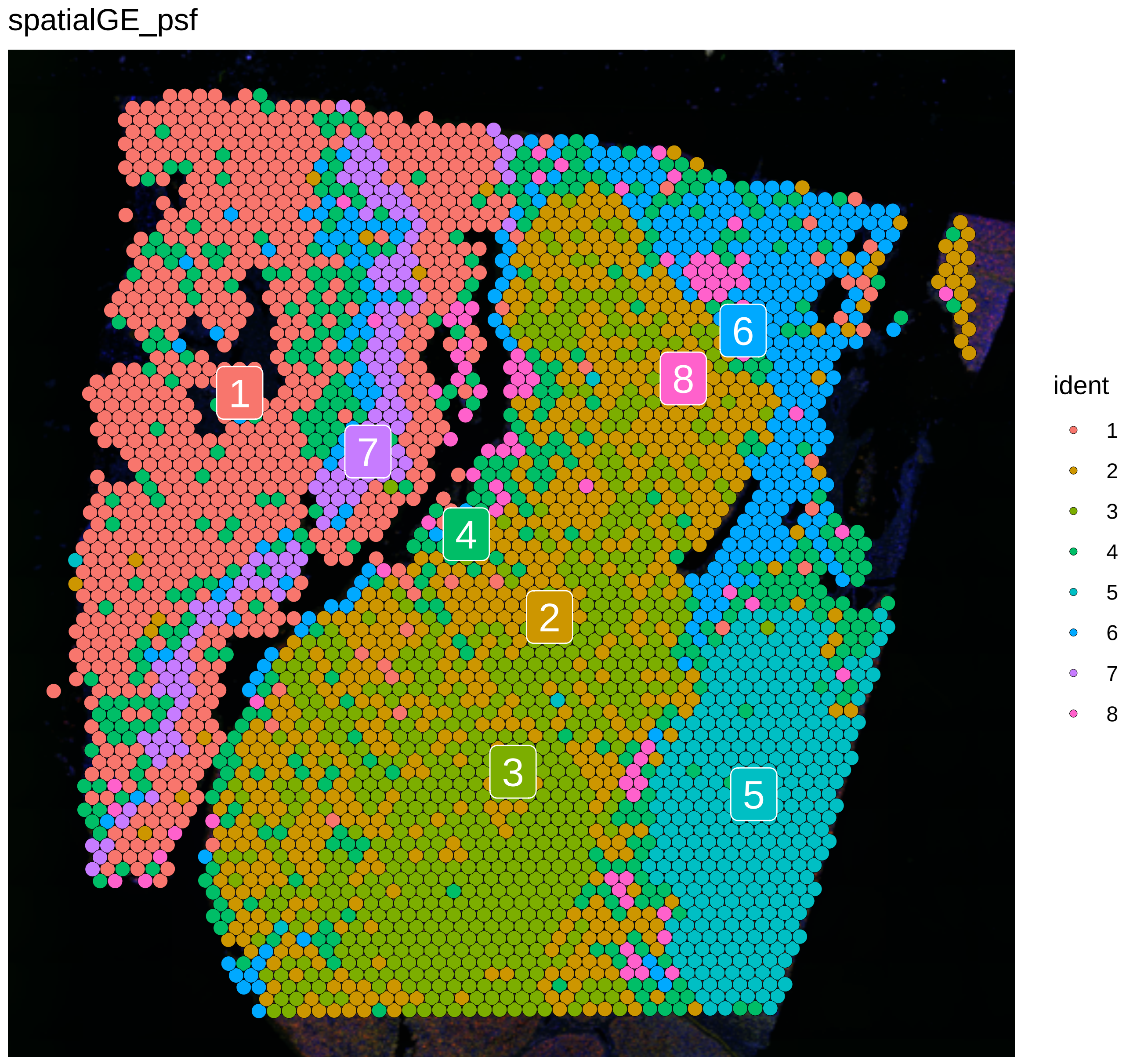

spatialGE_psf
1
2
3
4
5
6
7
8
ident
1
2
3
4
5
6
7
8

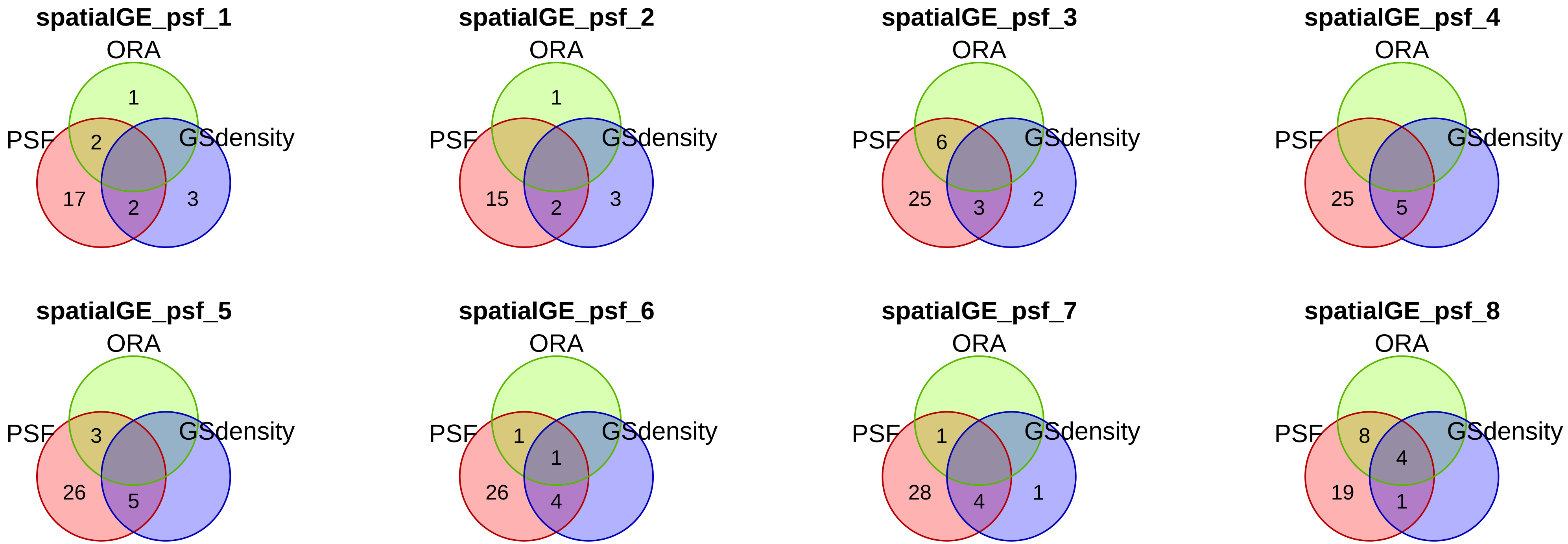
spatialGE_psf_1
ORA
PSF
GSdensity
1
2
17
2
3
spatialGE_psf_2
ORA
PSF
GSdensity
1
15
2
3
spatialGE_psf_3
ORA
PSF
GSdensity
6
25
3
2
spatialGE_psf_4
ORA
PSF
GSdensity
25
5
spatialGE_psf_5
ORA
PSF
GSdensity
3
26
5
spatialGE_psf_6
ORA
PSF
GSdensity
1
1
26
4
spatialGE_psf_7
ORA
PSF
GSdensity
1
28
4
1
spatialGE_psf_8
ORA
PSF
GSdensity
8
4
19
1

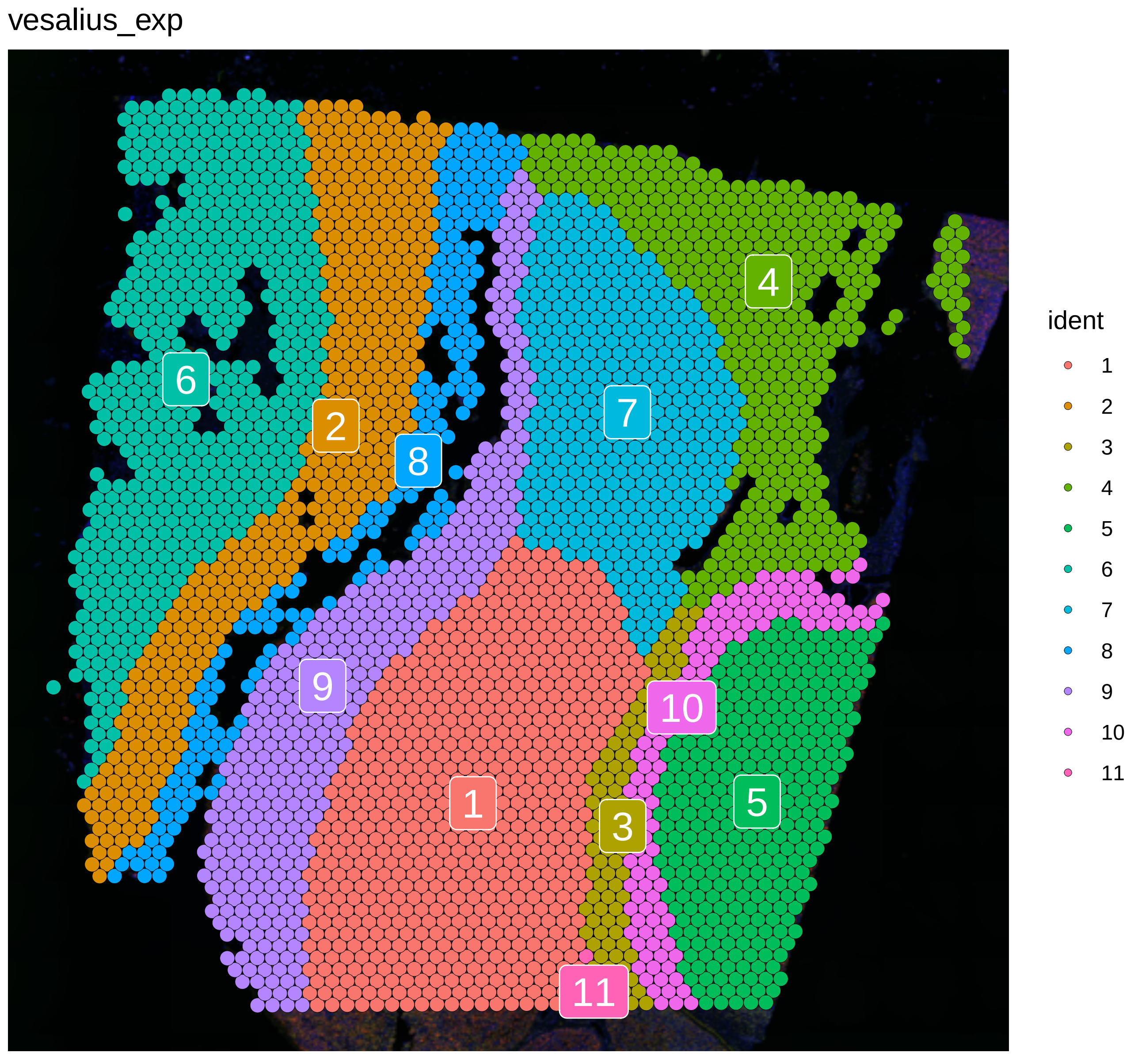

vesalius_exp
ident
1
2
3
4
5
6
7
8
9
10
11

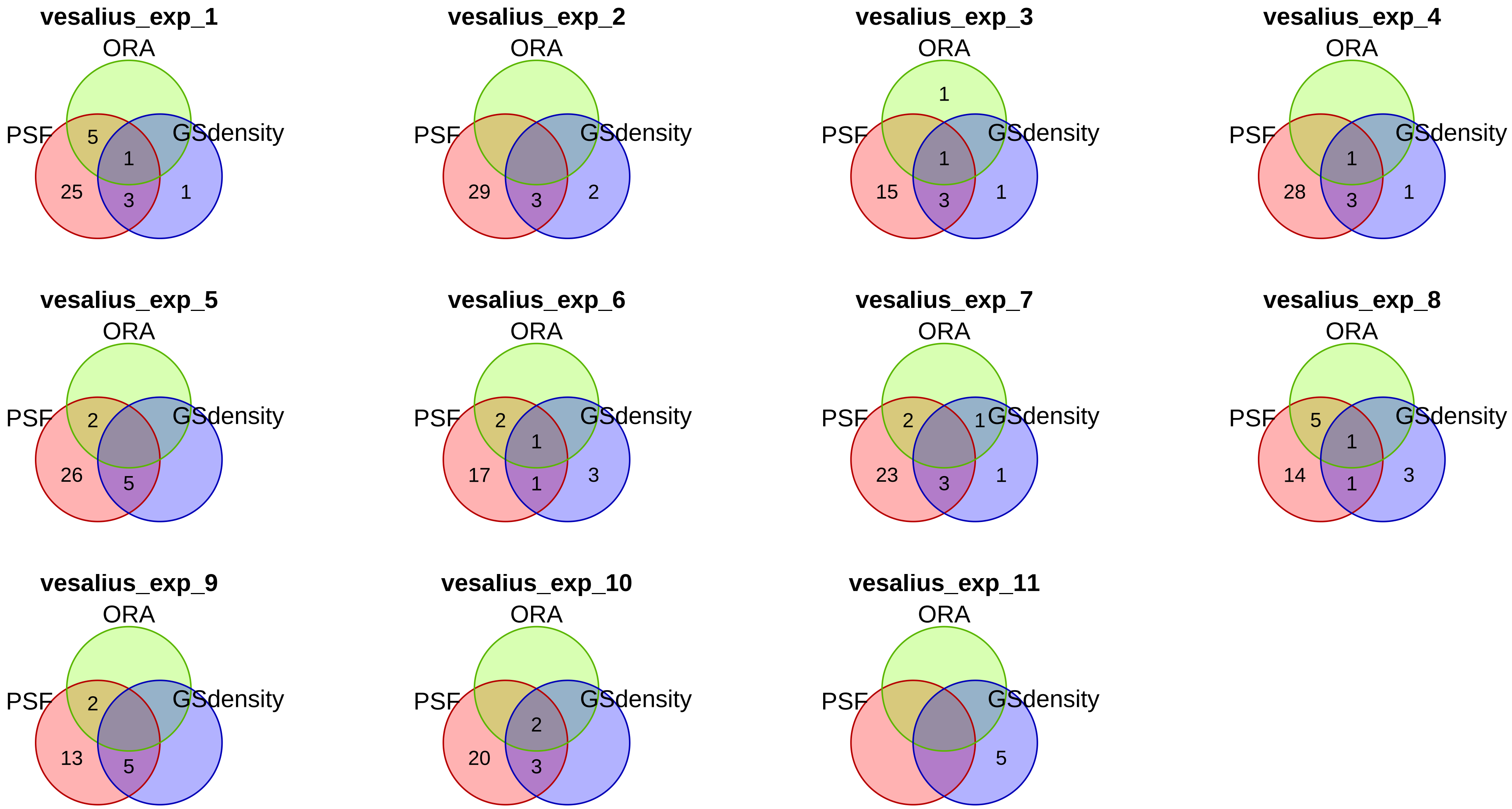
vesalius_exp_1
ORA
PSF
GSdensity
5
1
25
3
1
vesalius_exp_2
ORA
PSF
GSdensity
29
3
2
vesalius_exp_3
ORA
PSF
GSdensity
1
1
15
3
1
vesalius_exp_4
ORA
PSF
GSdensity
1
28
3
1
vesalius_exp_5
ORA
PSF
GSdensity
2
26
5
vesalius_exp_6
ORA
PSF
GSdensity
2
1
17
1
3
vesalius_exp_7
ORA
PSF
GSdensity
2
1
23
3
1
vesalius_exp_8
ORA
PSF
GSdensity
5
1
14
1
3
vesalius_exp_9
ORA
PSF
GSdensity
2
13
5
vesalius_exp_10
ORA
PSF
GSdensity
2
20
3
vesalius_exp_11
ORA
PSF
GSdensity
5

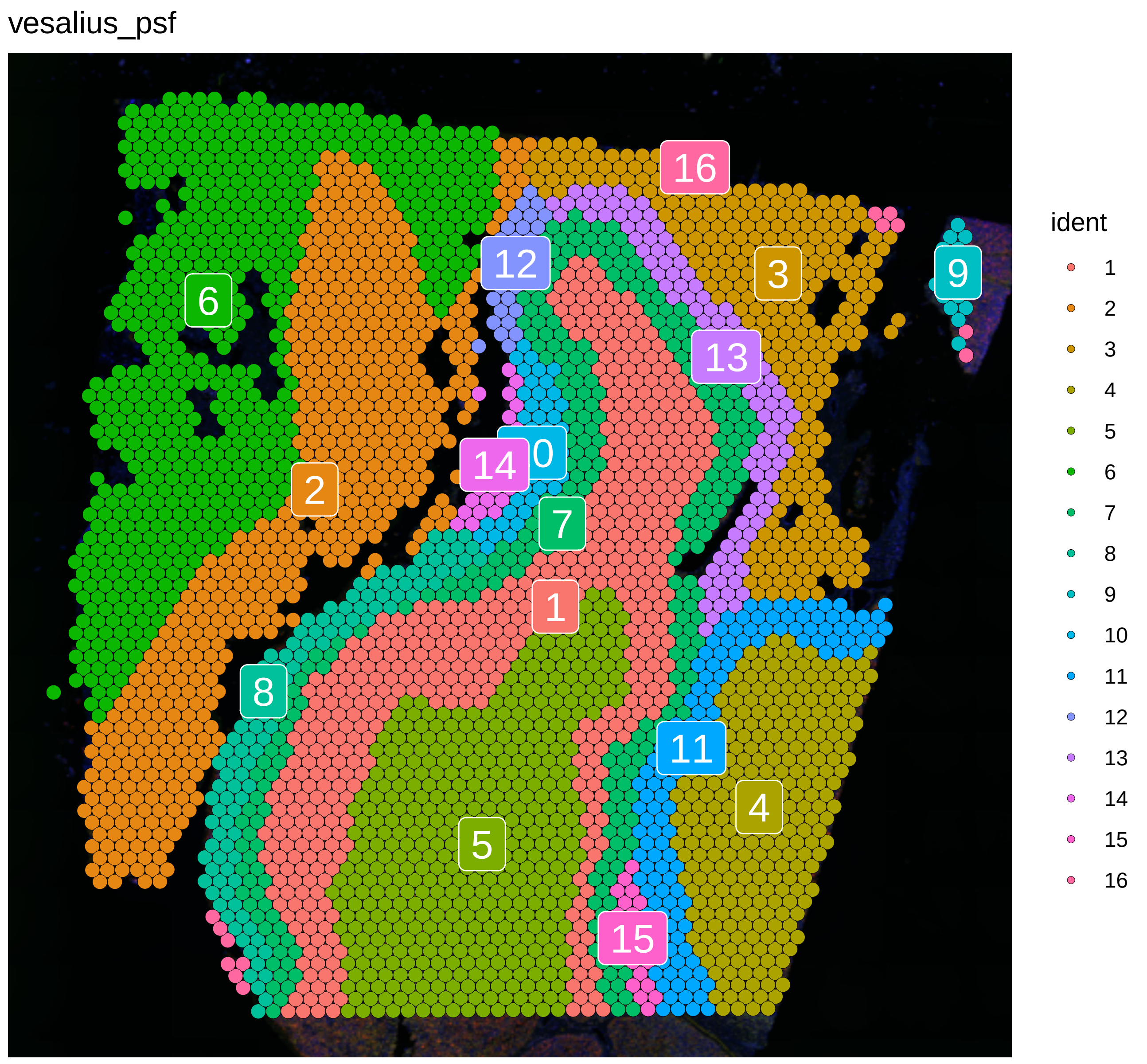
vesalius_psf
16
12
3
9
13
6
14
0
7
2
1
8
11
4
5
15
ident
1
2
3
4
5
6
7
8
9
10
11
12
13
14
15
16

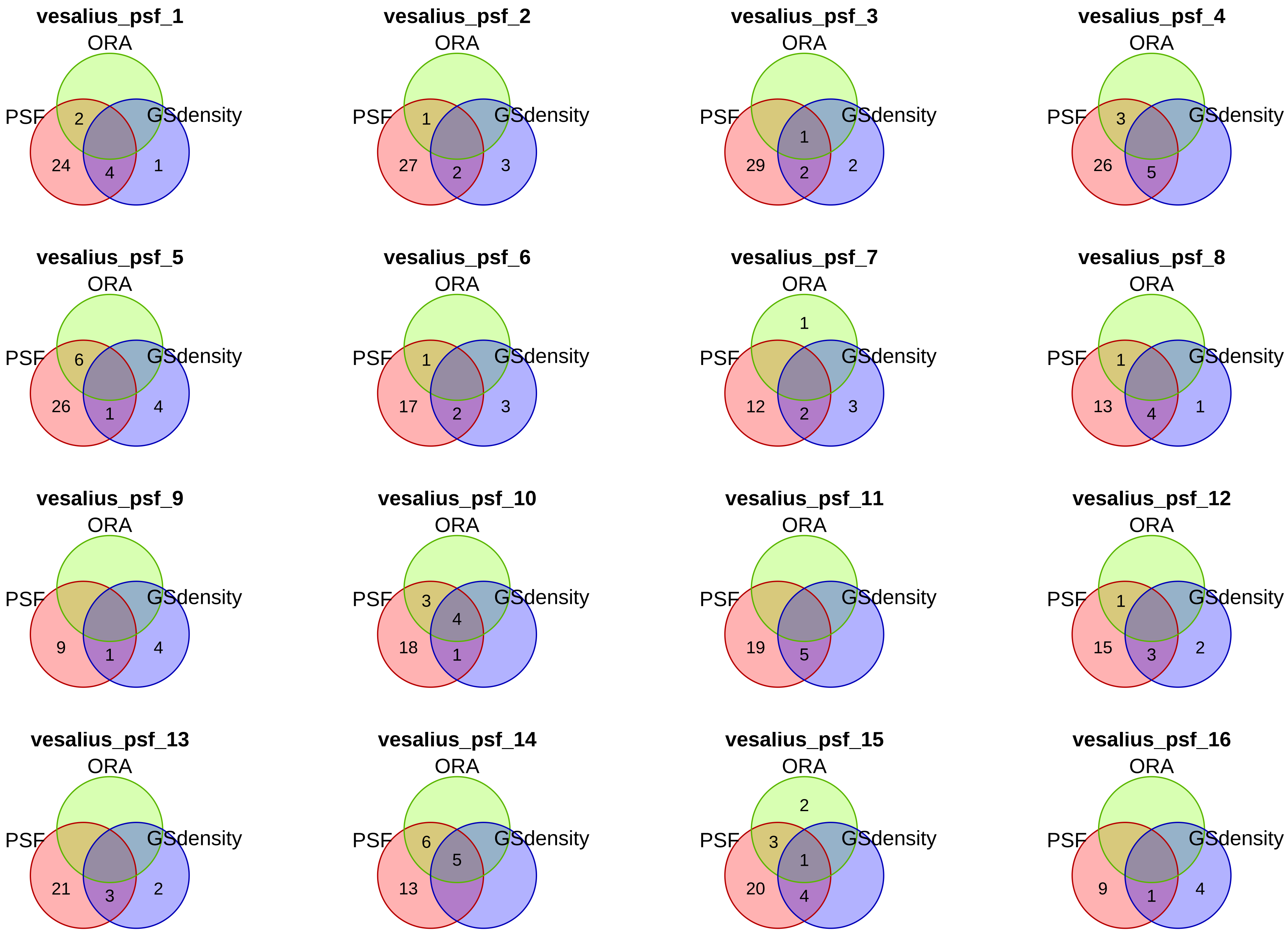
vesalius_psf_1
ORA
PSF
GSdensity
2
24
4
1
vesalius_psf_2
ORA
PSF
GSdensity
1
27
2
3
vesalius_psf_3
ORA
PSF
GSdensity
1
29
2
2
vesalius_psf_4
ORA
PSF
GSdensity
3
26
5
vesalius_psf_5
ORA
PSF
GSdensity
6
26
1
4
vesalius_psf_6
ORA
PSF
GSdensity
1
17
2
3
vesalius_psf_7
ORA
1
PSF
GSdensity
12
2
3
vesalius_psf_8
ORA
PSF
GSdensity
1
13
4
1
vesalius_psf_9
ORA
PSF
GSdensity
9
1
4
vesalius_psf_10
ORA
PSF
GSdensity
3
4
18
1
vesalius_psf_11
ORA
PSF
GSdensity
19
5
vesalius_psf_12
ORA
PSF
GSdensity
1
15
3
2
vesalius_psf_13
ORA
PSF
GSdensity
21
3
2
vesalius_psf_14
ORA
PSF
GSdensity
6
5
13
vesalius_psf_15
ORA
2
PSF
GSdensity
3
1
20
4
vesalius_psf_16
ORA
PSF
GSdensity
9
1
4

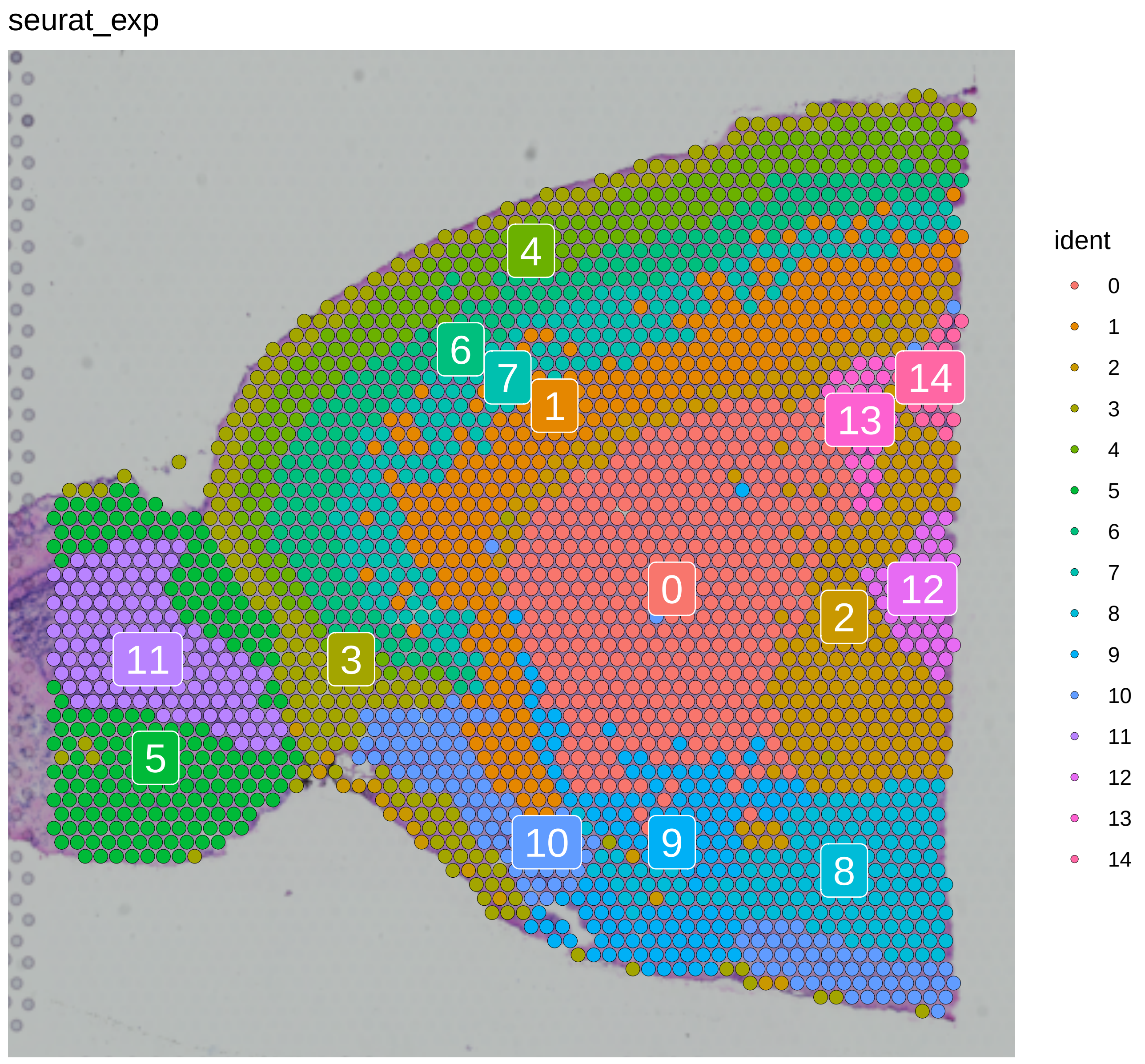

seurat_exp
4
6
7
1
14
13
0
12
2
11
3
5
10
9
8
ident
0
1
2
3
4
5
6
7
8
9
10
11
12
13
14

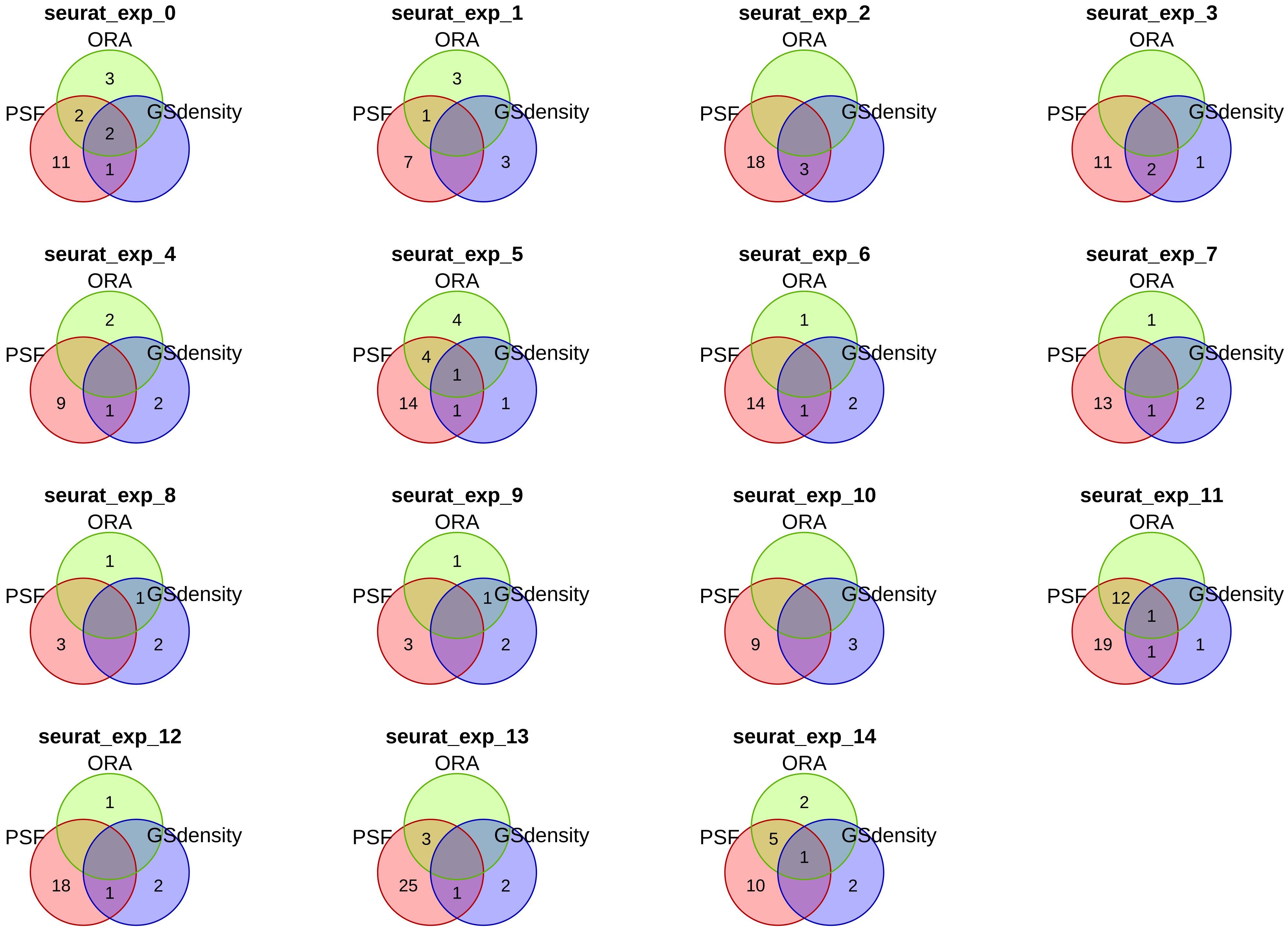
seurat_exp_0
ORA
PSF
GSdensity
3
2
2
11
1
seurat_exp_1
ORA
PSF
GSdensity
3
1
7
3
seurat_exp_2
ORA
PSF
GSdensity
18
3
seurat_exp_3
ORA
PSF
GSdensity
11
2
1
seurat_exp_4
ORA
PSF
GSdensity
2
9
1
2
seurat_exp_5
ORA
PSF
GSdensity
4
4
1
14
1
1
seurat_exp_6
ORA
PSF
GSdensity
1
14
1
2
seurat_exp_7
ORA
PSF
GSdensity
1
13
1
2
seurat_exp_8
ORA
PSF
GSdensity
1
1
3
2
seurat_exp_9
ORA
PSF
GSdensity
1
1
3
2
seurat_exp_10
ORA
PSF
GSdensity
9
3
seurat_exp_11
ORA
PSF
GSdensity
12
1
19
1
1
seurat_exp_12
ORA
PSF
GSdensity
1
18
1
2
seurat_exp_13
ORA
PSF
GSdensity
3
25
1
2
seurat_exp_14
ORA
PSF
GSdensity
2
5
1
10
2

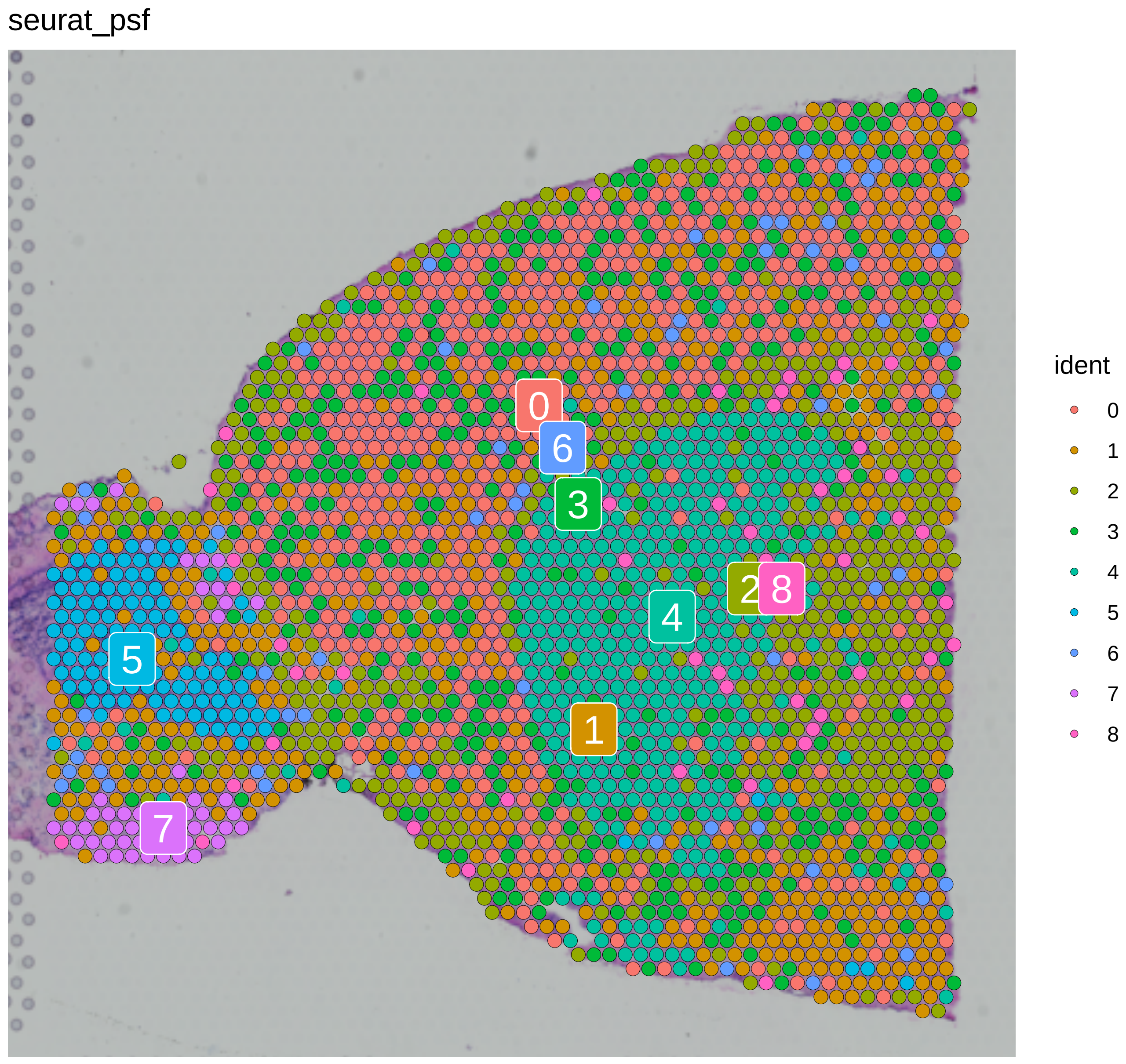
seurat_psf
ident
0
1
2
3
4
5
6
7
8

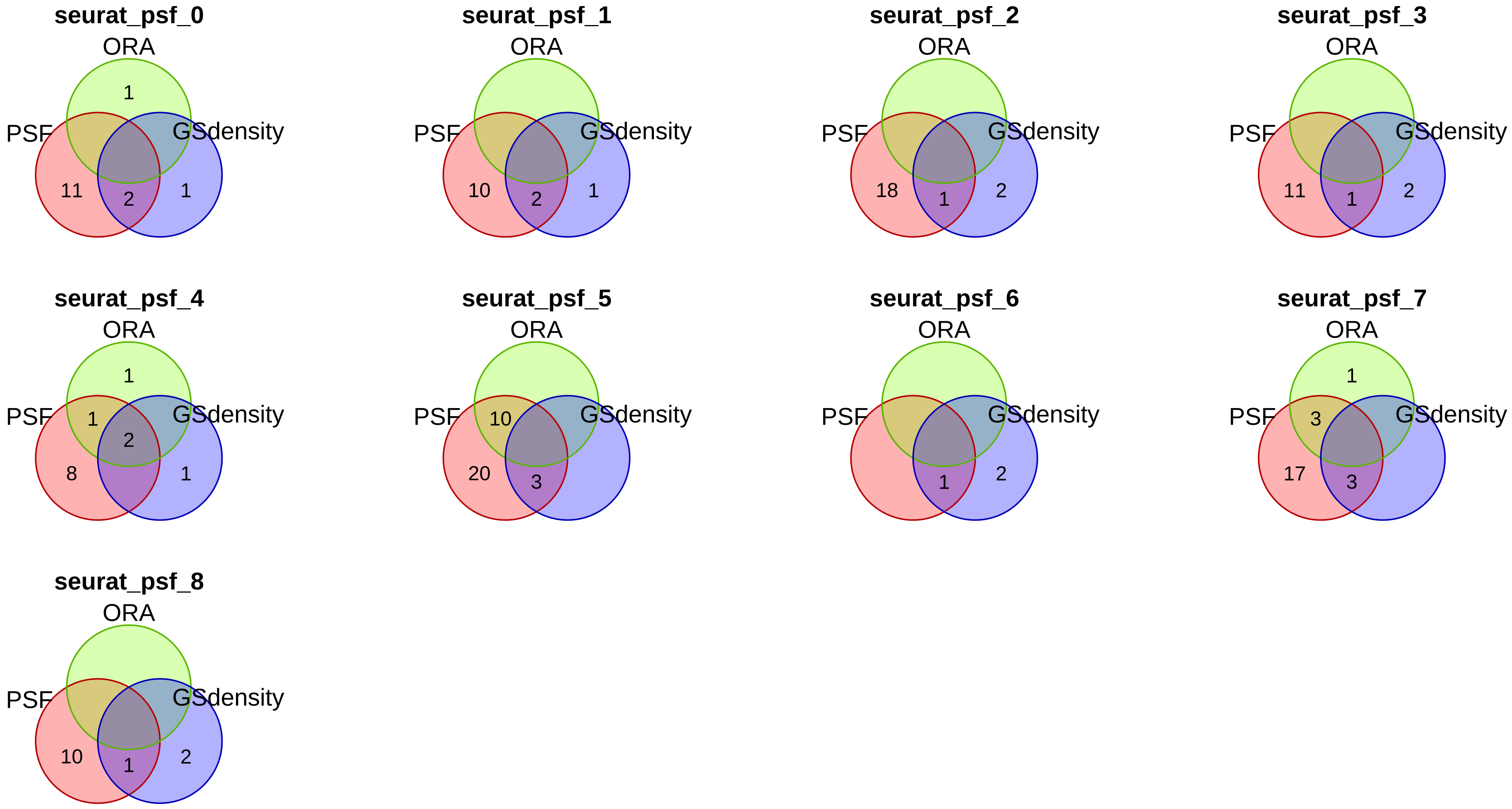
seurat_psf_0
ORA
PSF
GSdensity
1
11
2
1
seurat_psf_1
ORA
PSF
GSdensity
10
2
1
seurat_psf_2
ORA
PSF
GSdensity
18
1
2
seurat_psf_3
ORA
PSF
GSdensity
11
1
2
seurat_psf_4
ORA
PSF
GSdensity
1
1
2
8
1
seurat_psf_5
ORA
PSF
GSdensity
10
20
3
seurat_psf_6
ORA
PSF
GSdensity
1
2
seurat_psf_7
ORA
PSF
GSdensity
1
3
17
3
seurat_psf_8
ORA
PSF
GSdensity
10
1
2

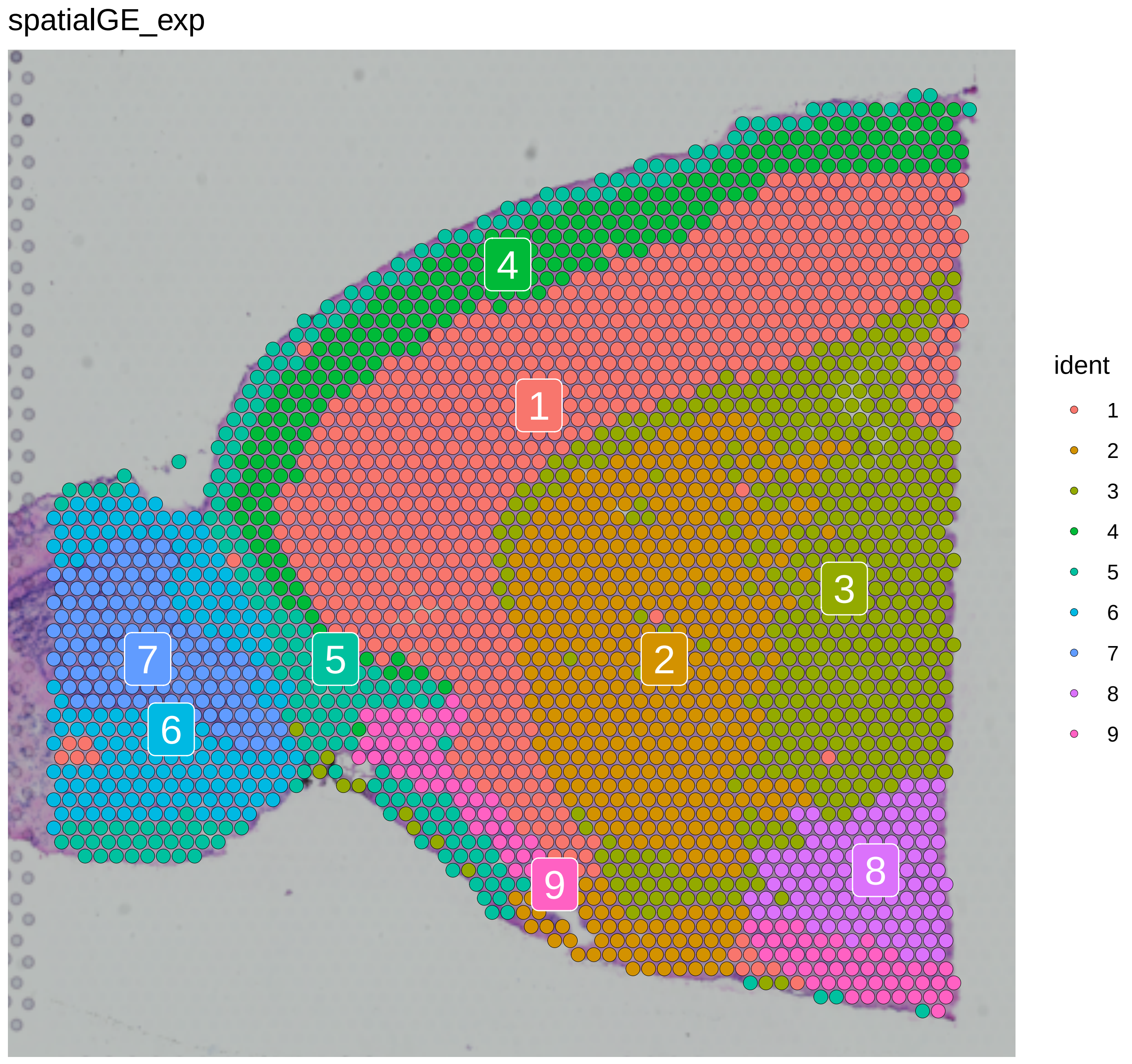
spatialGE_exp
4
1
3
7
5
2
6
9
8
ident
1
2
3
4
5
6
7
8
9

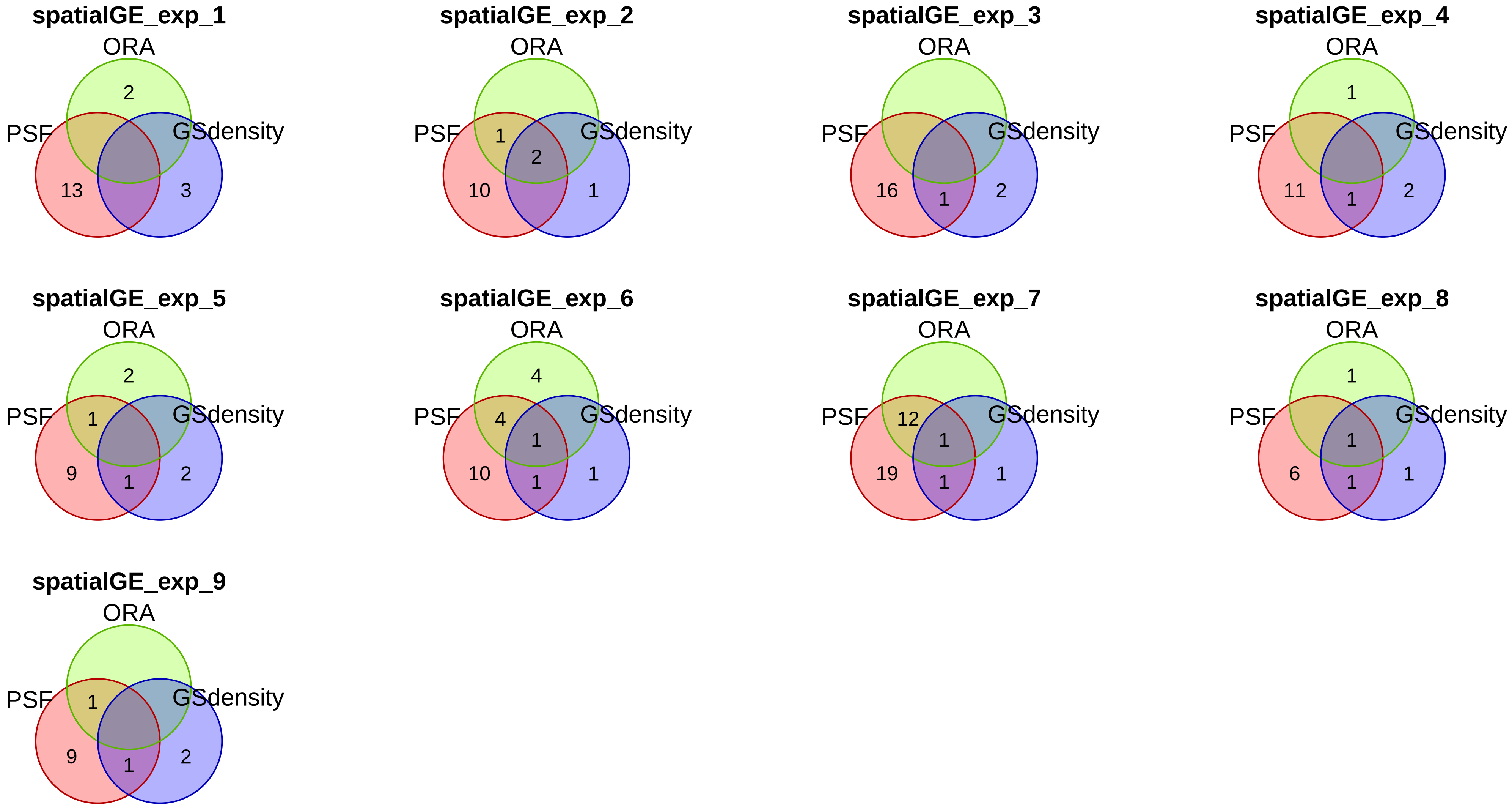
spatialGE_exp_1
ORA
PSF
GSdensity
2
13
3
spatialGE_exp_2
ORA
PSF
GSdensity
1
2
10
1
spatialGE_exp_3
ORA
PSF
GSdensity
16
1
2
spatialGE_exp_4
ORA
PSF
GSdensity
1
11
1
2
spatialGE_exp_5
ORA
PSF
GSdensity
2
1
9
1
2
spatialGE_exp_6
ORA
PSF
GSdensity
4
4
1
10
1
1
spatialGE_exp_7
ORA
PSF
GSdensity
12
1
19
1
1
spatialGE_exp_8
ORA
PSF
GSdensity
1
1
6
1
1
spatialGE_exp_9
ORA
PSF
GSdensity
1
9
1
2

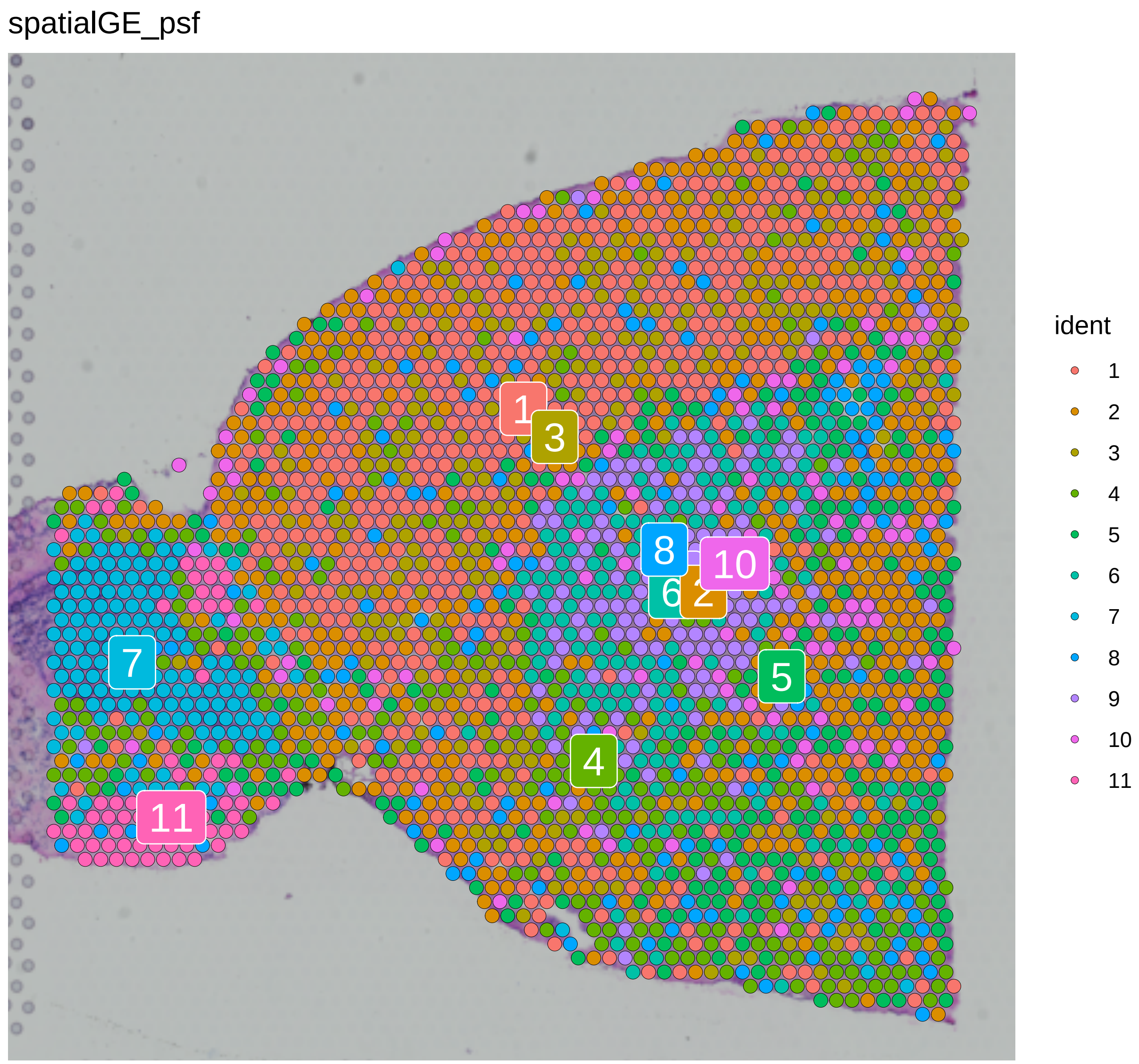

spatialGE_psf
1
2
3
4
5
6
7
8
10
11
ident
1
2
3
4
5
6
7
8
9
10
11

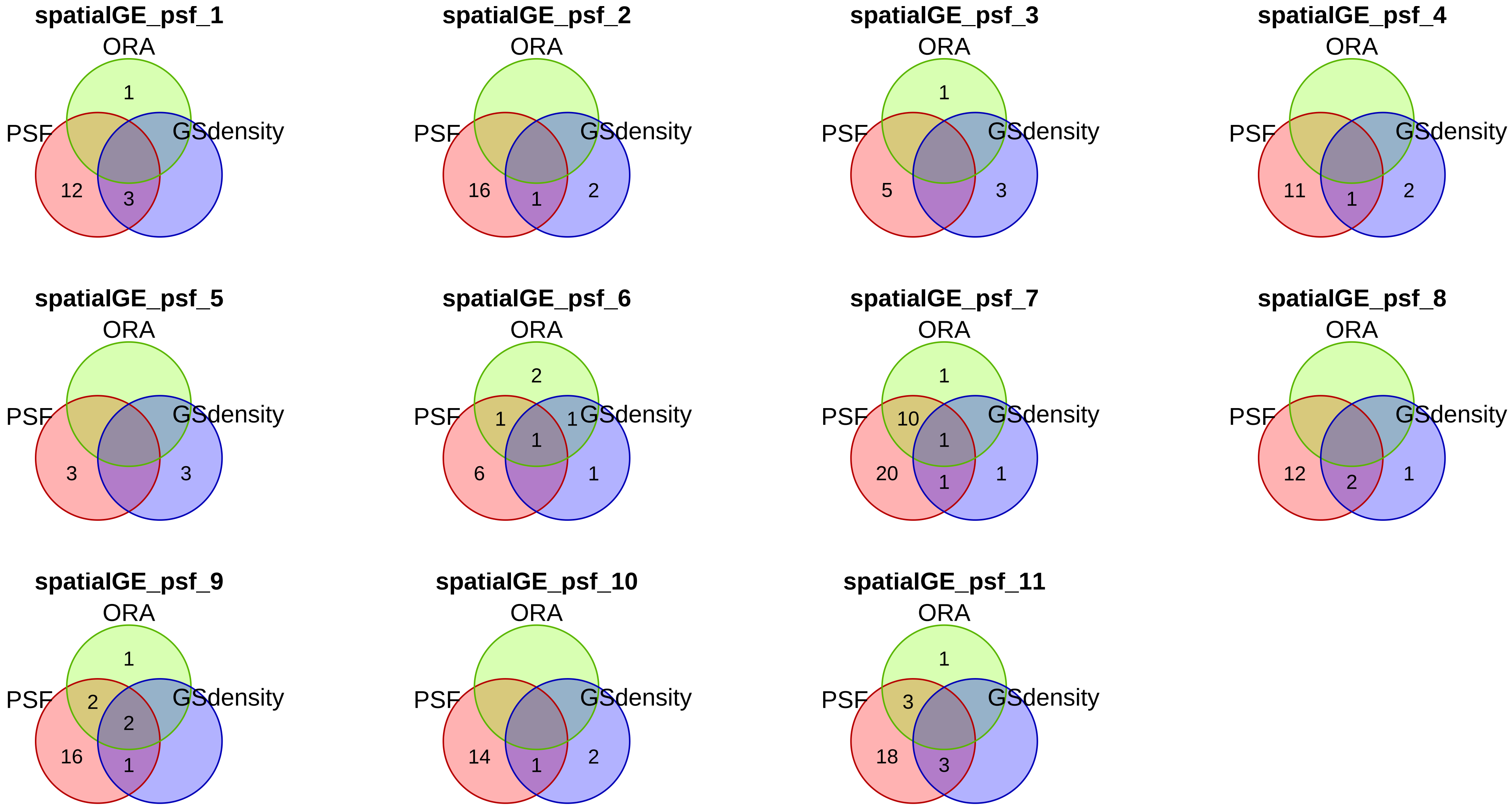
spatialGE_psf_1
ORA
PSF
GSdensity
1
12
3
spatialGE_psf_2
ORA
PSF
GSdensity
16
1
2
spatialGE_psf_3
ORA
PSF
GSdensity
1
5
3
spatialGE_psf_4
ORA
PSF
GSdensity
11
1
2
spatialGE_psf_5
ORA
PSF
GSdensity
3
3
spatialGE_psf_6
ORA
PSF
GSdensity
2
1
1
1
6
1
spatialGE_psf_7
ORA
PSF
GSdensity
1
10
1
20
1
1
spatialGE_psf_8
ORA
PSF
GSdensity
12
2
1
spatialGE_psf_9
ORA
PSF
GSdensity
1
2
2
16
1
spatialGE_psf_10
ORA
PSF
GSdensity
14
1
2
spatialGE_psf_11
ORA
PSF
GSdensity
1
3
18
3

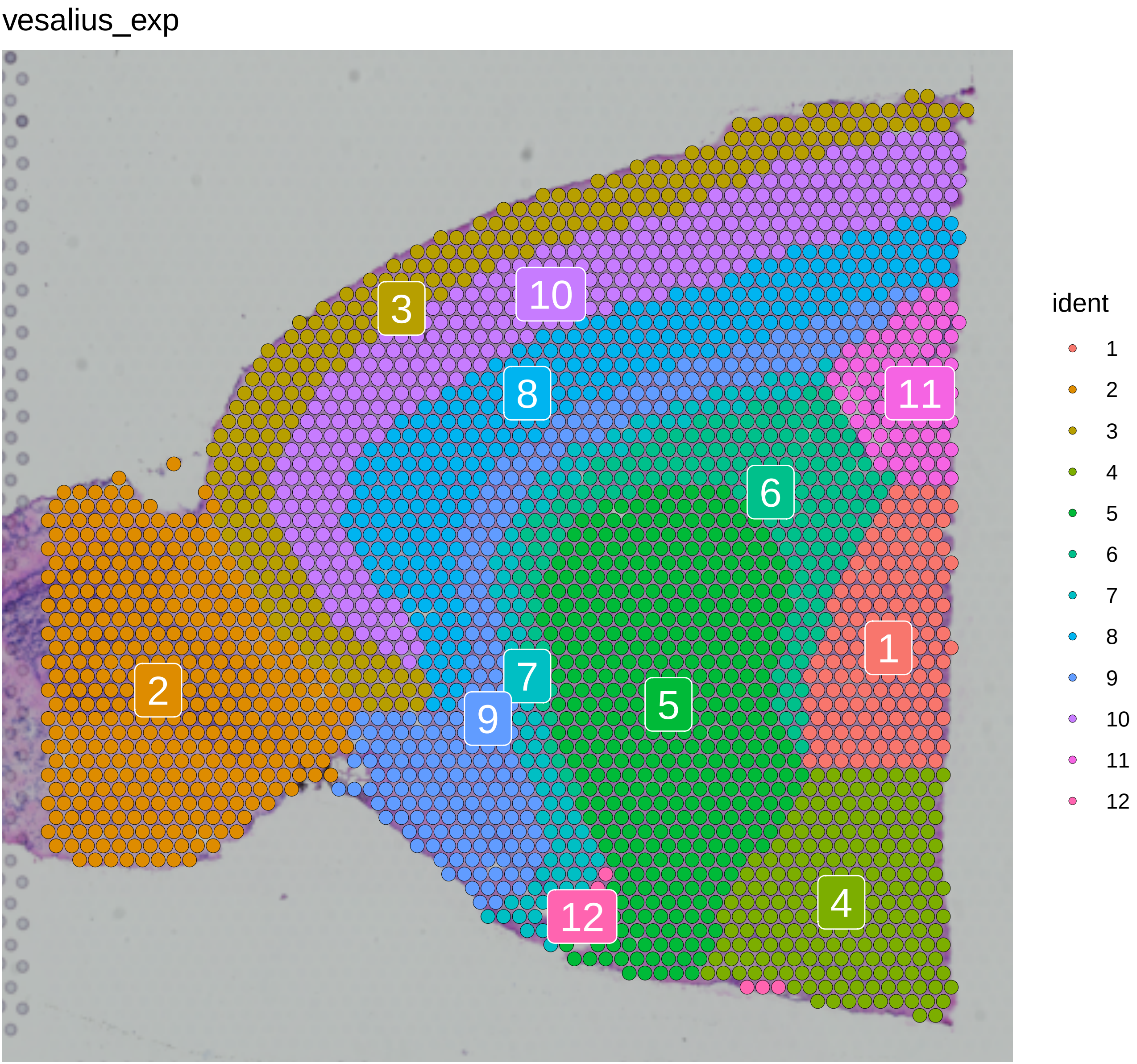

vesalius_exp
3
10
8
11
6
1
2
7
9
5
4
12
ident
1
2
3
4
5
6
7
8
9
10
11
12

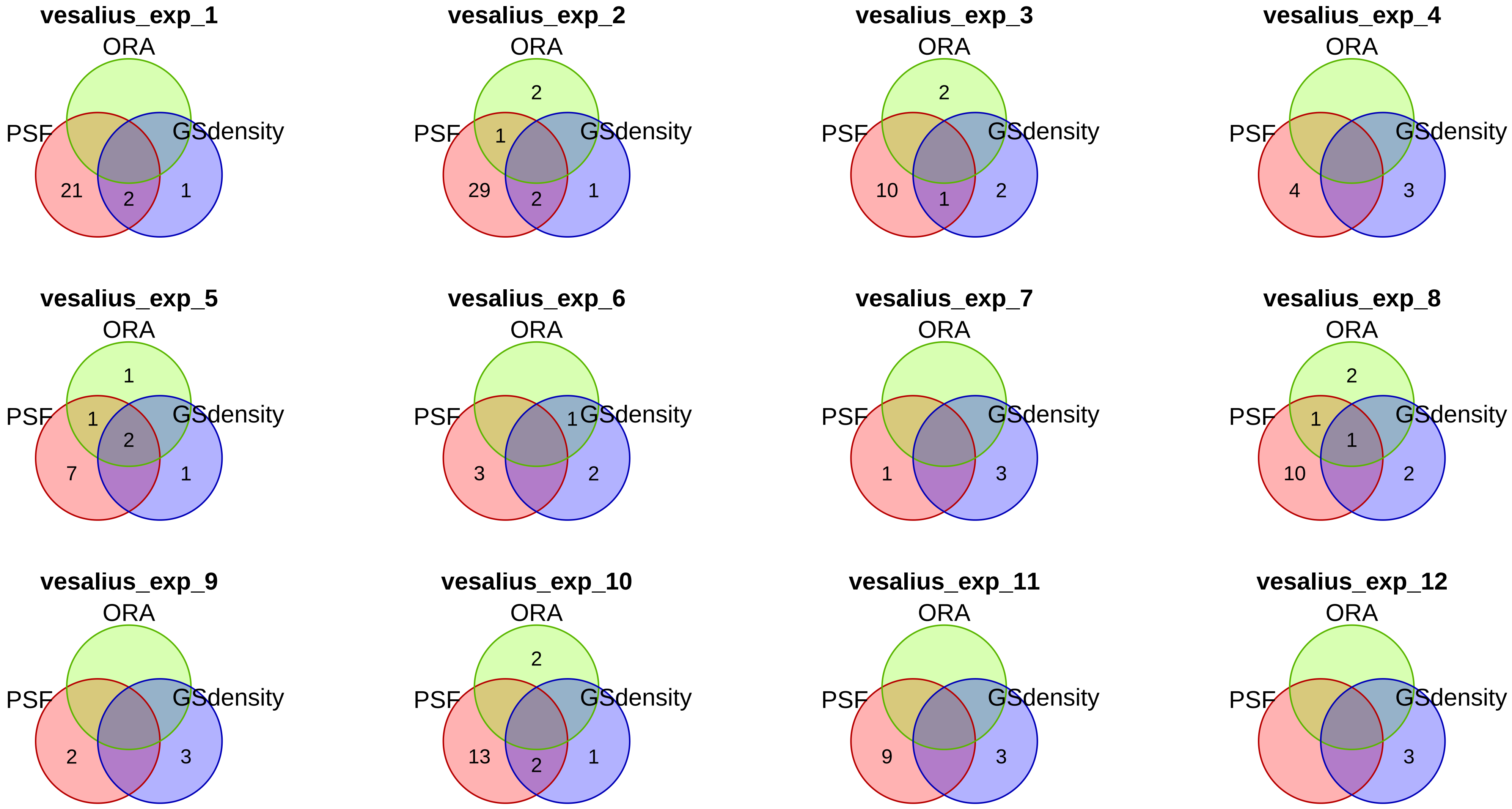
vesalius_exp_1
ORA
PSF
GSdensity
21
2
1
vesalius_exp_2
ORA
2
PSF
1
GSdensity
29
2
1
vesalius_exp_3
ORA
2
PSF
GSdensity
10
1
2
vesalius_exp_4
ORA
PSF
GSdensity
4
3
vesalius_exp_5
ORA
1
PSF
1
GSdensity
2
7
1
vesalius_exp_6
ORA
PSF
1
GSdensity
3
2
vesalius_exp_7
ORA
PSF
GSdensity
1
3
vesalius_exp_8
ORA
2
PSF
1
GSdensity
1
10
2
vesalius_exp_9
ORA
PSF
GSdensity
2
3
vesalius_exp_10
ORA
2
PSF
GSdensity
13
2
1
vesalius_exp_11
ORA
PSF
GSdensity
9
3
vesalius_exp_12
ORA
PSF
GSdensity
3

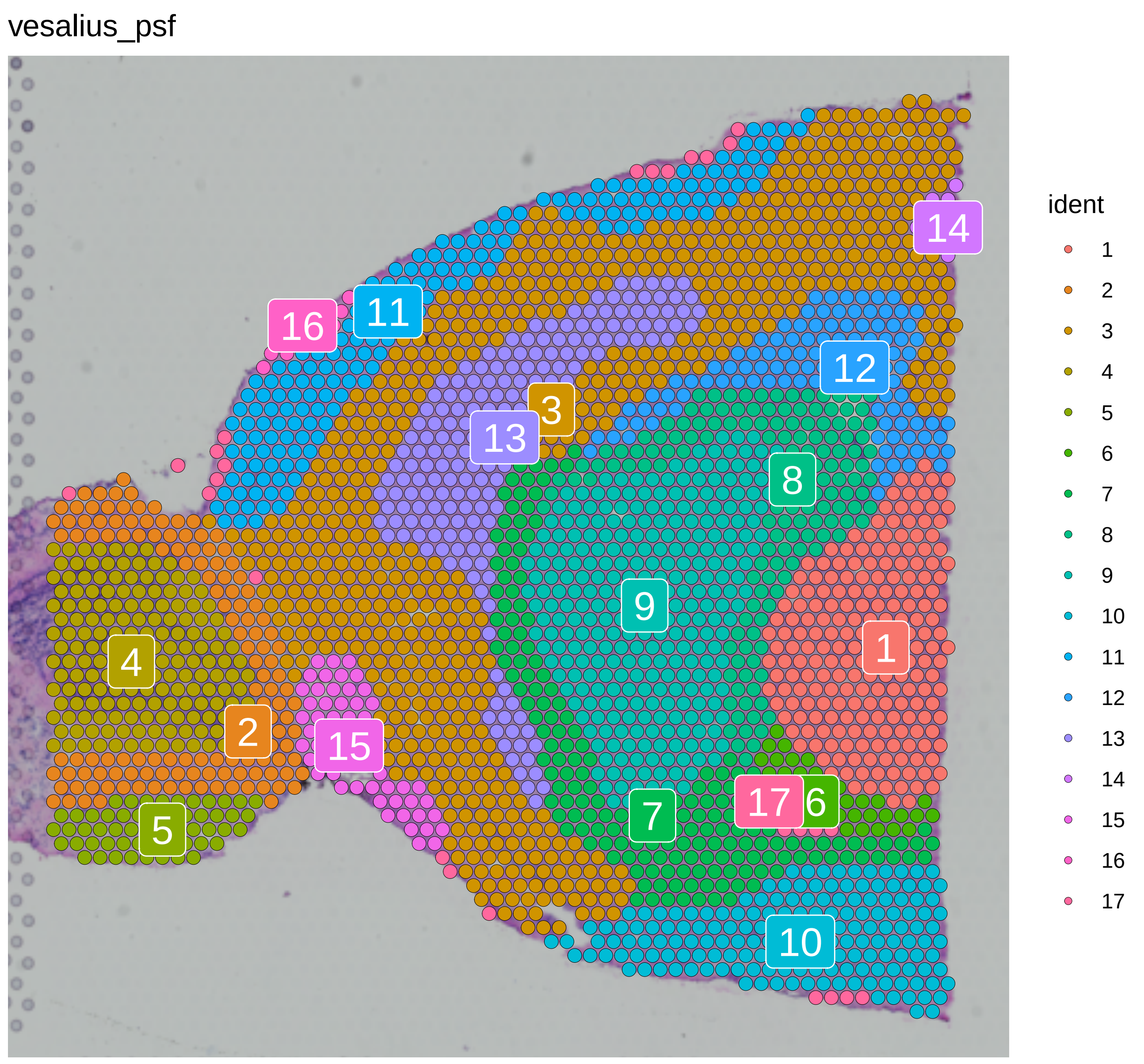

vesalius_psf
16
11
14
12
3
13
8
9
1
4
2
15
17
6
7
5
10
ident
1
2
3
4
5
6
7
8
9
10
11
12
13
14
15
16
17

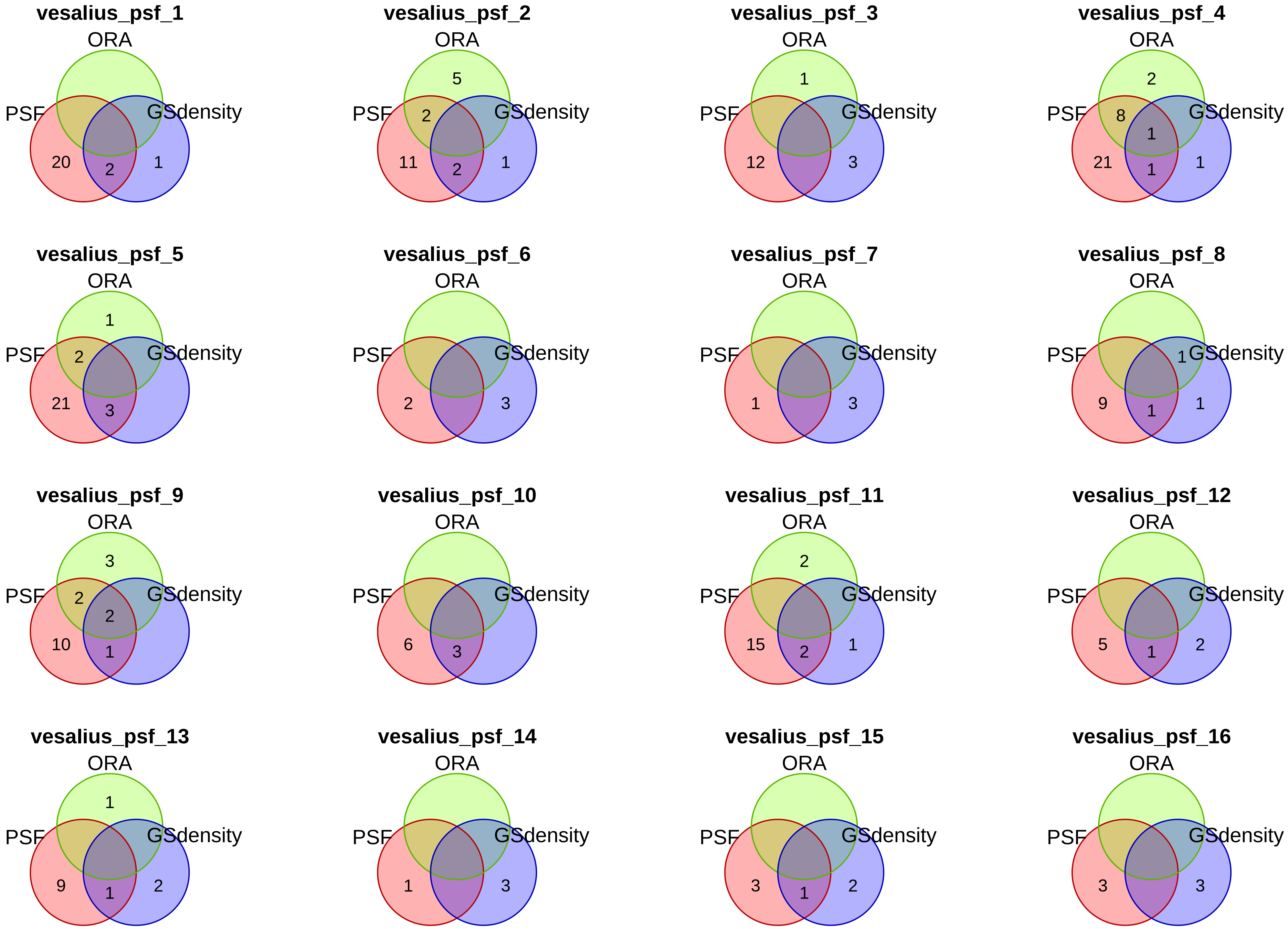
vesalius_psf_1
ORA
PSF
GSdensity
20
2
1
vesalius_psf_2
ORA
5
PSF
2
GSdensity
11
2
1
vesalius_psf_3
ORA
1
PSF
GSdensity
12
3
vesalius_psf_4
ORA
2
PSF
8
GSdensity
1
21
1
1
vesalius_psf_5
ORA
1
PSF
2
GSdensity
21
3
vesalius_psf_6
ORA
PSF
GSdensity
2
3
vesalius_psf_7
ORA
PSF
GSdensity
1
3
vesalius_psf_8
ORA
PSF
1
GSdensity
9
1
1
vesalius_psf_9
ORA
3
PSF
2
GSdensity
2
10
1
vesalius_psf_10
ORA
PSF
GSdensity
6
3
vesalius_psf_11
ORA
2
PSF
GSdensity
15
2
1
vesalius_psf_12
ORA
PSF
GSdensity
5
1
2
vesalius_psf_13
ORA
1
PSF
GSdensity
9
1
2
vesalius_psf_14
ORA
PSF
GSdensity
1
3
vesalius_psf_15
ORA
PSF
GSdensity
3
1
2
vesalius_psf_16
ORA
PSF
GSdensity
3
3

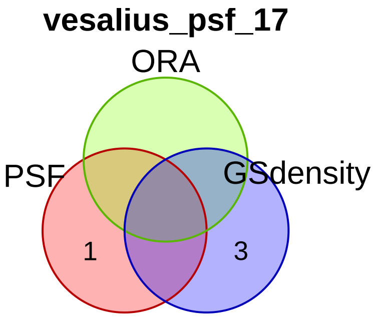
vesalius_psf_17
ORA
PSF
GSdensity
1
3
