# Supplementary material for: Topology-aware pathway analysis of spatial transcriptomics
Source: PeerJ. 2025 Aug 14;13:e19729. doi: 10.7717/peerj.19729 (PMC12358110; doi:10.7717/peerj.19729)
Supplement: Supplemental Information 6 — Pathway-wise visualization of log2 mean activity values calculated using the PSF algorithm. Each page corresponds to a single pathway, displaying 8 boxplots representing different tissue types. Within each boxplot, box groups correspond to varying pseudobulk sizes that is, the number of cells aggregated to compute gene expression. This aggregation was applied to mitigate the impact of dropout events and zero-expression values common in single-cell data. Across most tissue types and pathways, the pathway activity values begin to stabilize when 10-12 or more cells are aggregated, suggesting this range is an effective threshold for reliable activity estimation in downstream analyses. [file peerj-13-19729-s006.pdf]

Overview of pathway activity stability across tissue types and pseudobulk sizes. This multi-page figure presents pathway-wise visualization of log2 mean activity values calculated using the PSF algorithm. Each page corresponds to a single pathway, displaying 8 boxplots representing different tissue types. Within each boxplot, box groups correspond to varying pseudobulk sizes that is, the number of cells aggregated to compute gene expression. This aggregation was applied to mitigate the impact of dropout events and zero-expression values common in single-cell data. Across most tissue types and pathways, the pathway activity values begin to stabilize when 10-12 or more cells are aggregated, suggesting this range is an effective threshold for reliable activity estimation in downstream analyses.

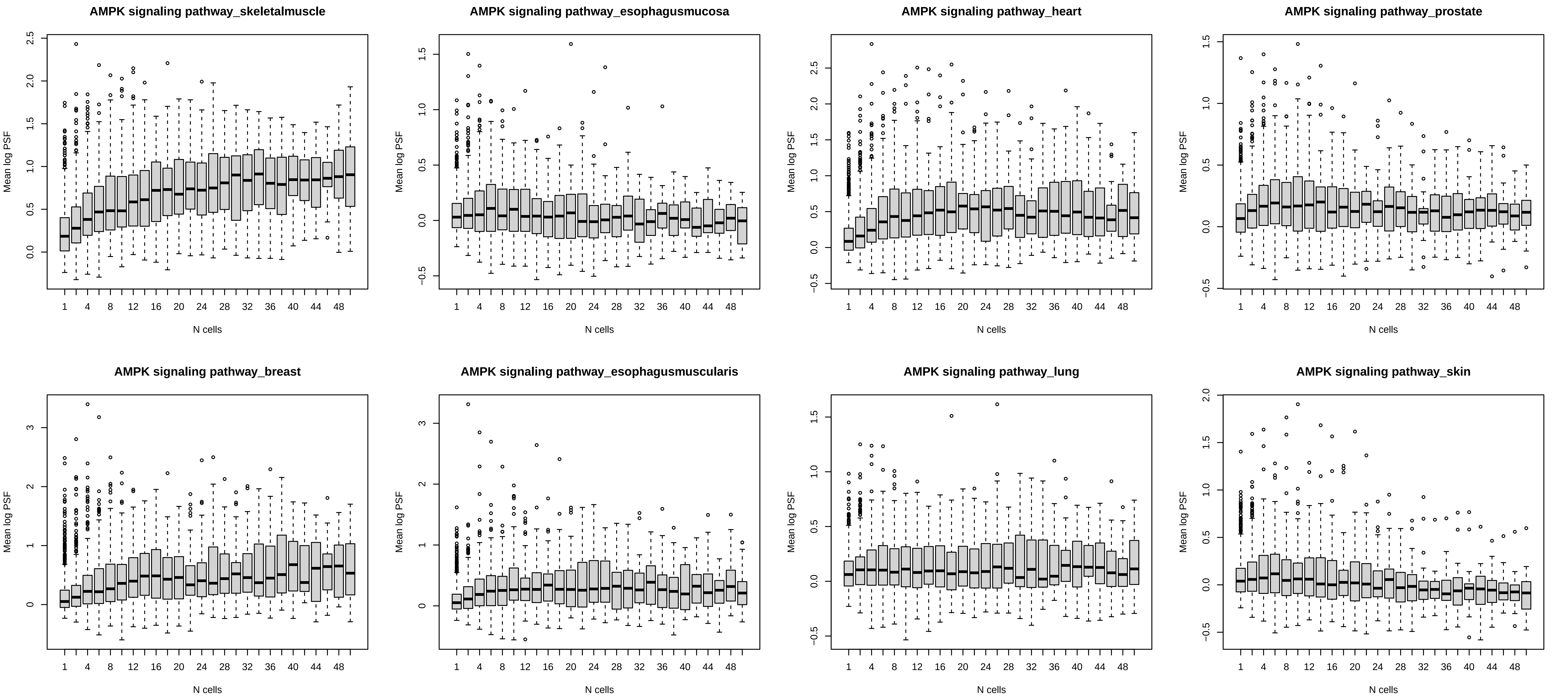
AMPK signaling pathway_skeletalmuscle
AMPK signaling pathway_esophagusmucosa
AMPK signaling pathway_heart
AMPK signaling pathway_prostate
AMPK signaling pathway_breast
AMPK signaling pathway_esophagusmuscularis
AMPK signaling pathway_lung
AMPK signaling pathway_skin
Mean log PSF
N cells
1 4 8 12 16 20 24 28 32 36 40 44 48

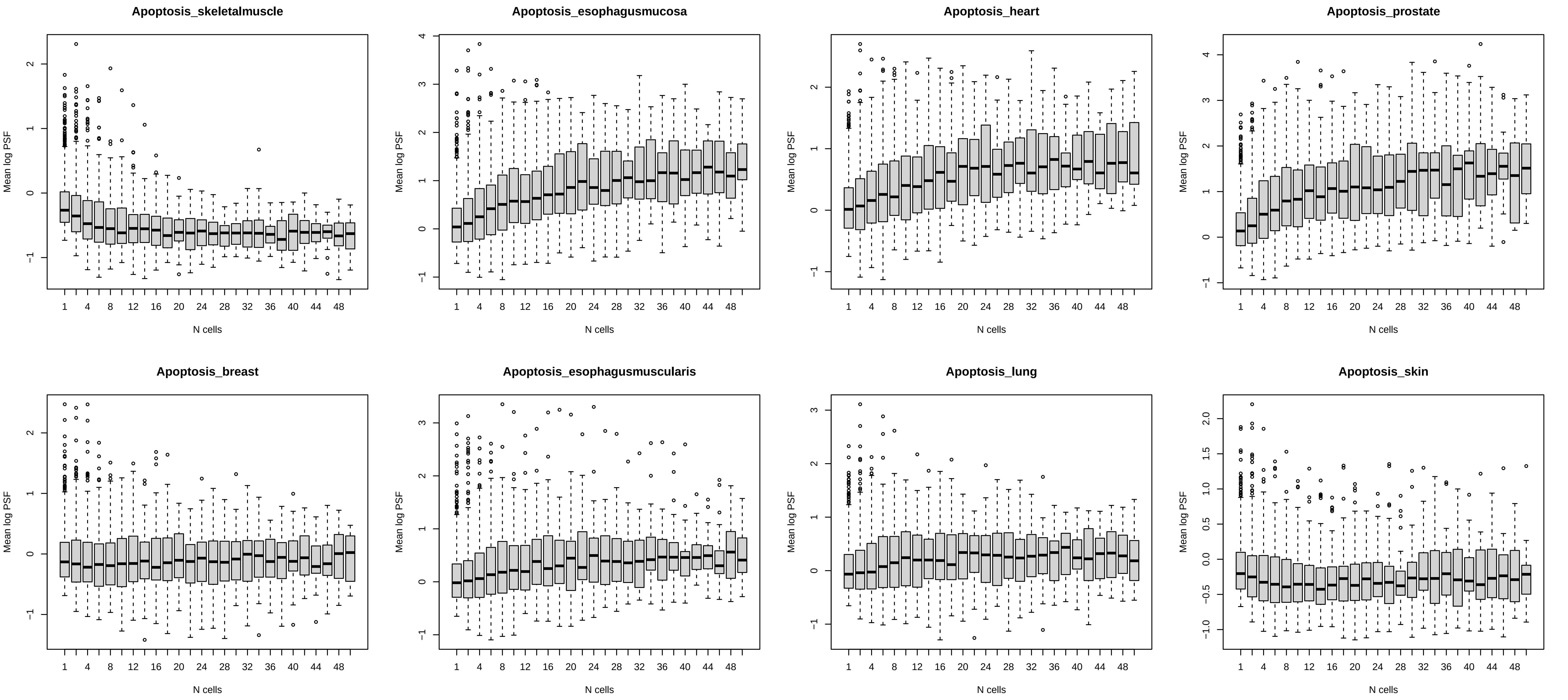

Apoptosis_skeletalmuscle
Apoptosis_esophagusmucosa
Apoptosis_heart
Apoptosis_prostate
Apoptosis_breast
Apoptosis_esophagusmuscularis
Apoptosis_lung
Apoptosis_skin
Mean log PSF
N cells

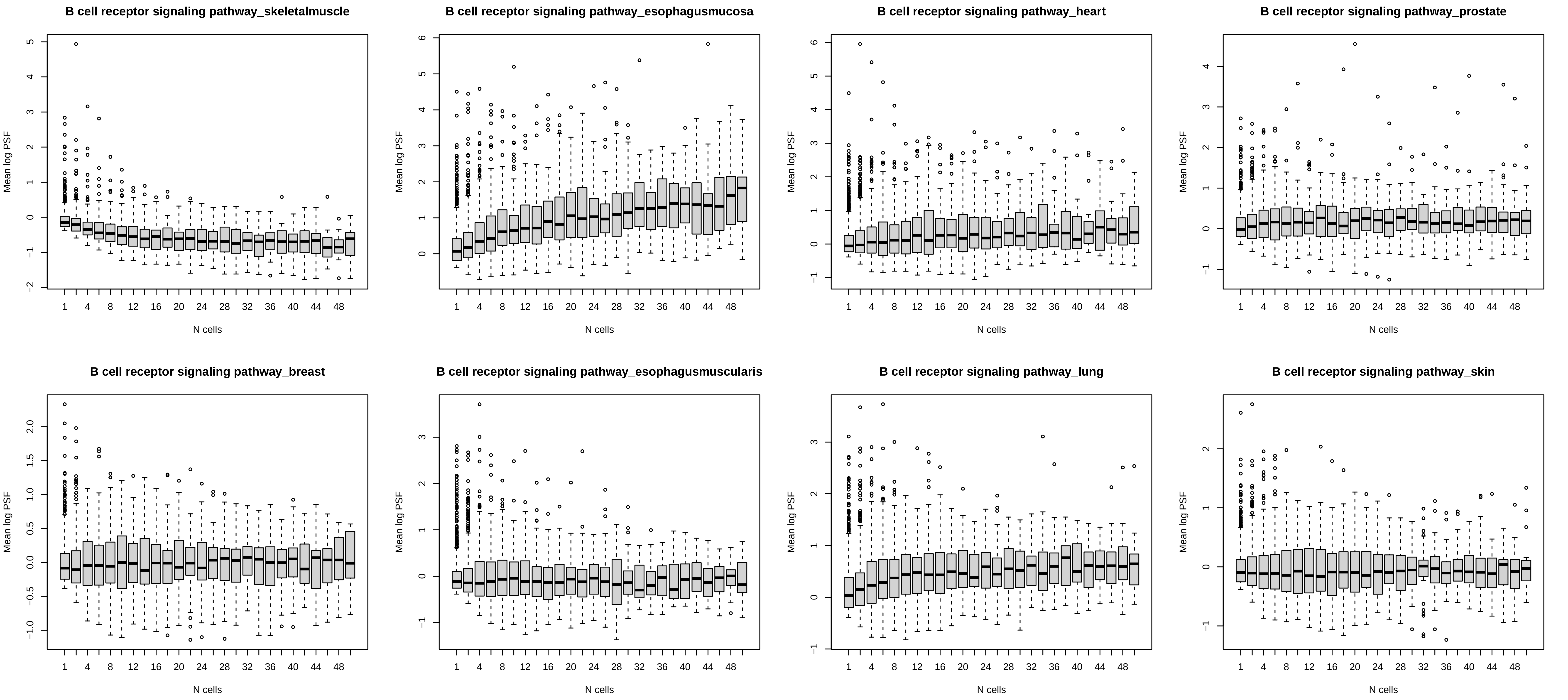
B cell receptor signaling pathway_skeletalmuscle
B cell receptor signaling pathway_esophagusmucosa
B cell receptor signaling pathway_heart
B cell receptor signaling pathway_prostate
B cell receptor signaling pathway_breast
B cell receptor signaling pathway_esophagusmuscularis
B cell receptor signaling pathway_lung
B cell receptor signaling pathway_skin
Mean log PSF
N cells

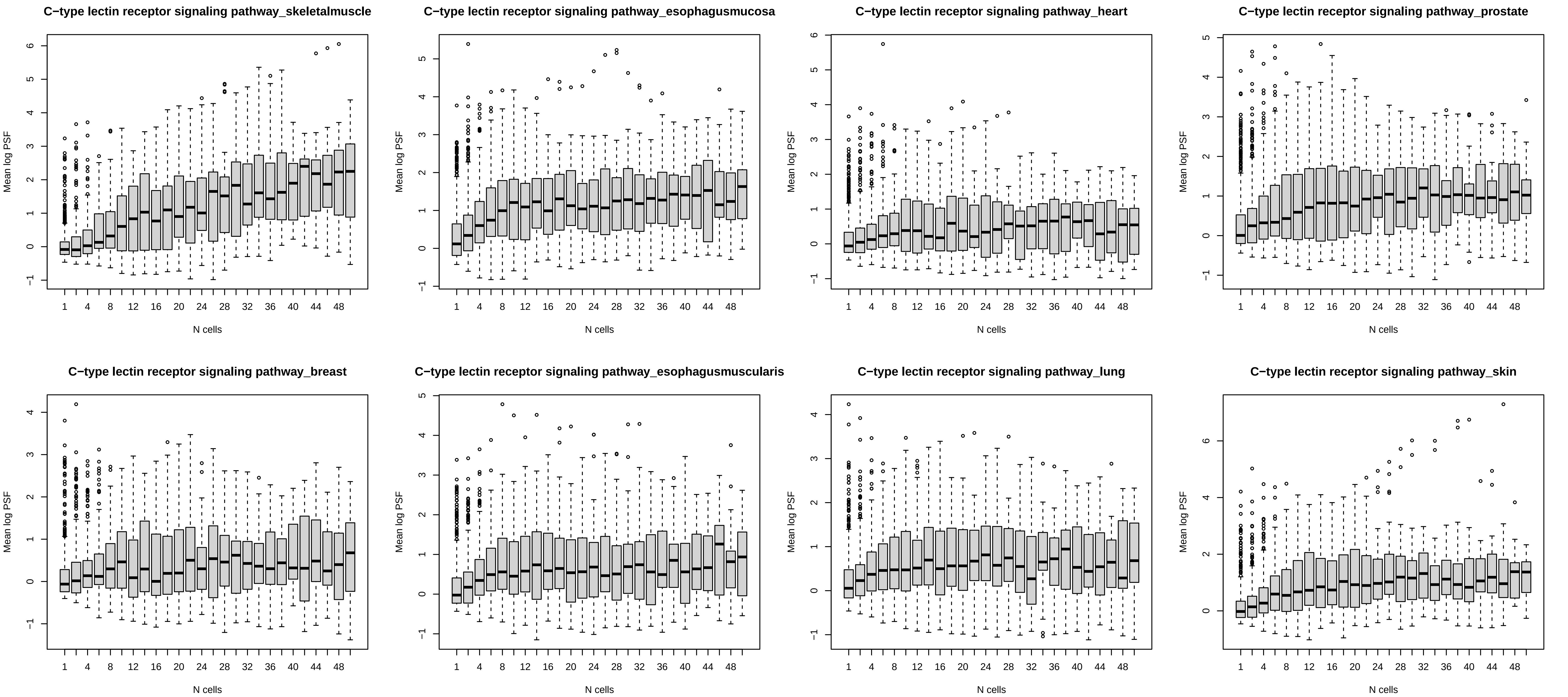

C−type lectin receptor signaling pathway_skeletalmuscle
C−type lectin receptor signaling pathway_esophagusmucosa
C−type lectin receptor signaling pathway_heart
C−type lectin receptor signaling pathway_prostate
C−type lectin receptor signaling pathway_breast
C−type lectin receptor signaling pathway_esophagusmuscularis
C−type lectin receptor signaling pathway_lung
C−type lectin receptor signaling pathway_skin
Mean log PSF
N cells
1 4 8 12 16 20 24 28 32 36 40 44 48

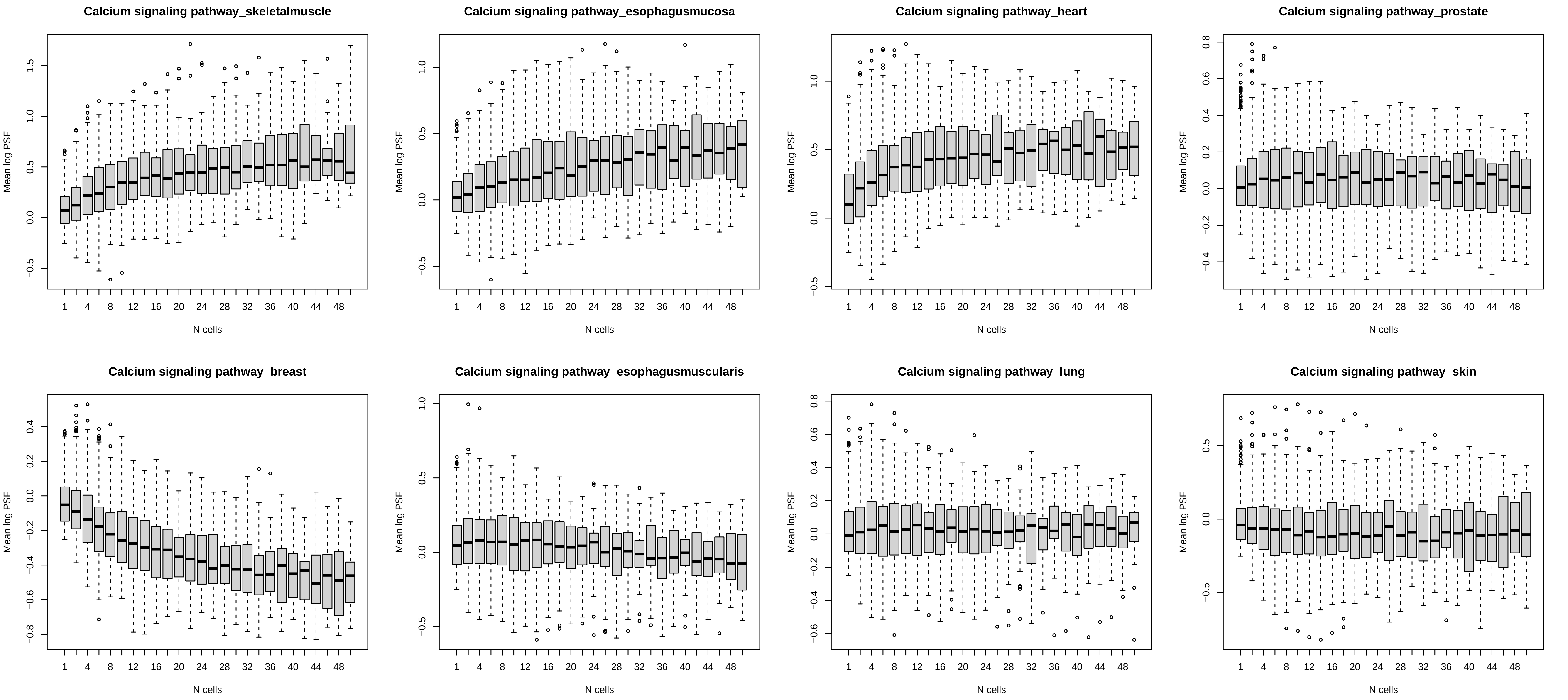
Calcium signaling pathway_skeletalmuscle
Calcium signaling pathway_esophagusmucosa
Calcium signaling pathway_heart
Calcium signaling pathway_prostate
Calcium signaling pathway_breast
Calcium signaling pathway_esophagusmuscularis
Calcium signaling pathway_lung
Calcium signaling pathway_skin
Mean log PSF
N cells
1 4 8 12 16 20 24 28 32 36 40 44 48

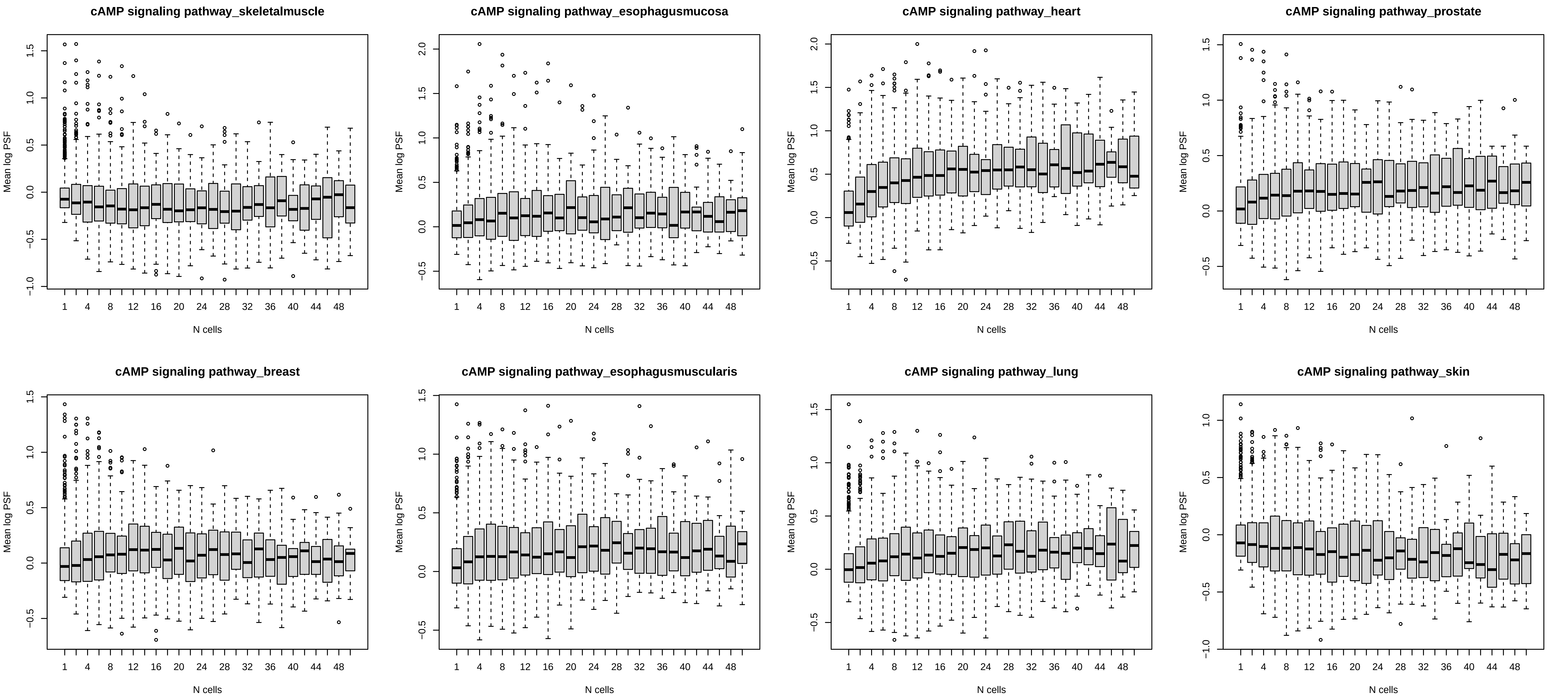

cAMP signaling pathway_skeletalmuscle
cAMP signaling pathway_esophagusmucosa
cAMP signaling pathway_heart
cAMP signaling pathway_prostate
cAMP signaling pathway_breast
cAMP signaling pathway_esophagusmuscularis
cAMP signaling pathway_lung
cAMP signaling pathway_skin
Mean log PSF
N cells
1 4 8 12 16 20 24 28 32 36 40 44 48

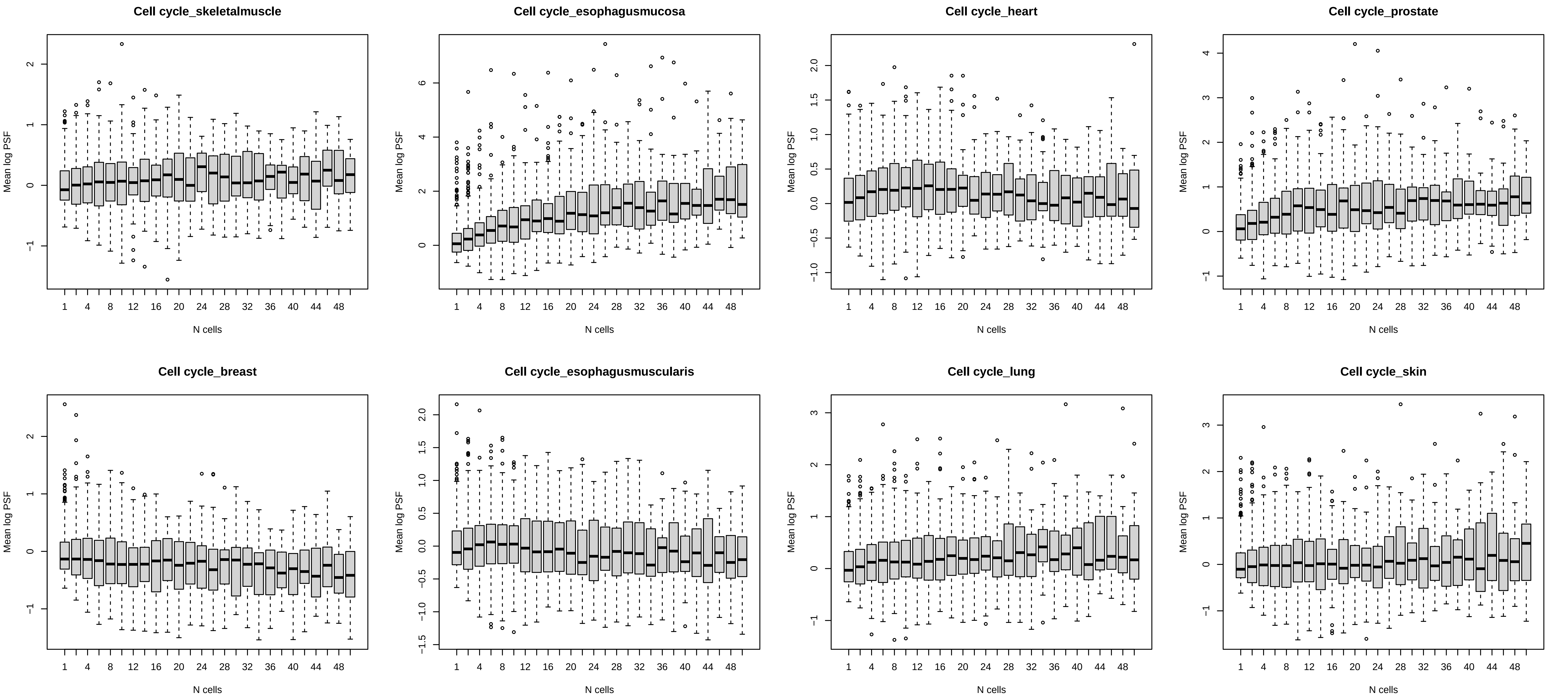
Cell cycle_skeletalmuscle
Cell cycle_esophagusmucosa
Cell cycle_heart
Cell cycle_prostate
Cell cycle_breast
Cell cycle_esophagusmuscularis
Cell cycle_lung
Cell cycle_skin
Mean log PSF
N cells

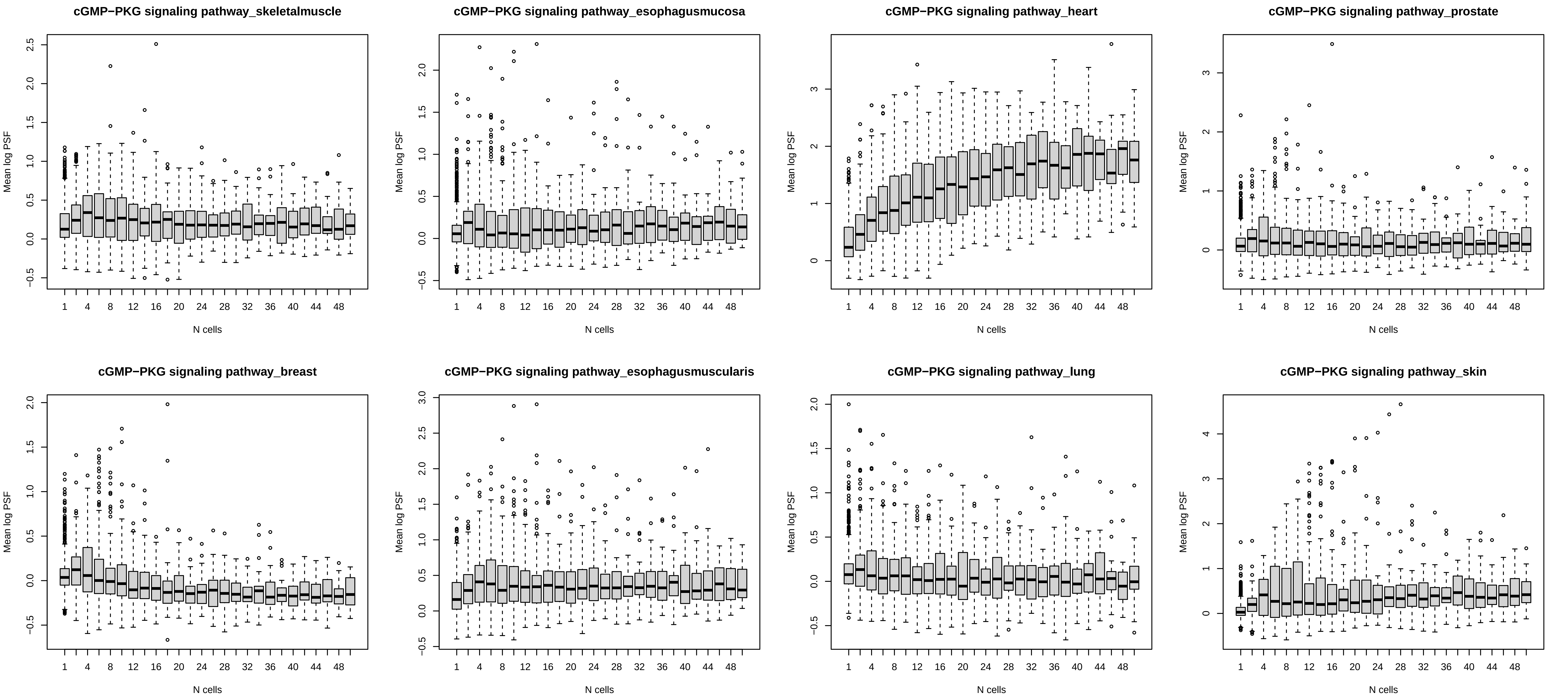
cGMP−PKG signaling pathway_skeletalmuscle
cGMP−PKG signaling pathway_esophagusmucosa
cGMP−PKG signaling pathway_heart
cGMP−PKG signaling pathway_prostate
cGMP−PKG signaling pathway_breast
cGMP−PKG signaling pathway_esophagusmuscularis
cGMP−PKG signaling pathway_lung
cGMP−PKG signaling pathway_skin
Mean log PSF
N cells

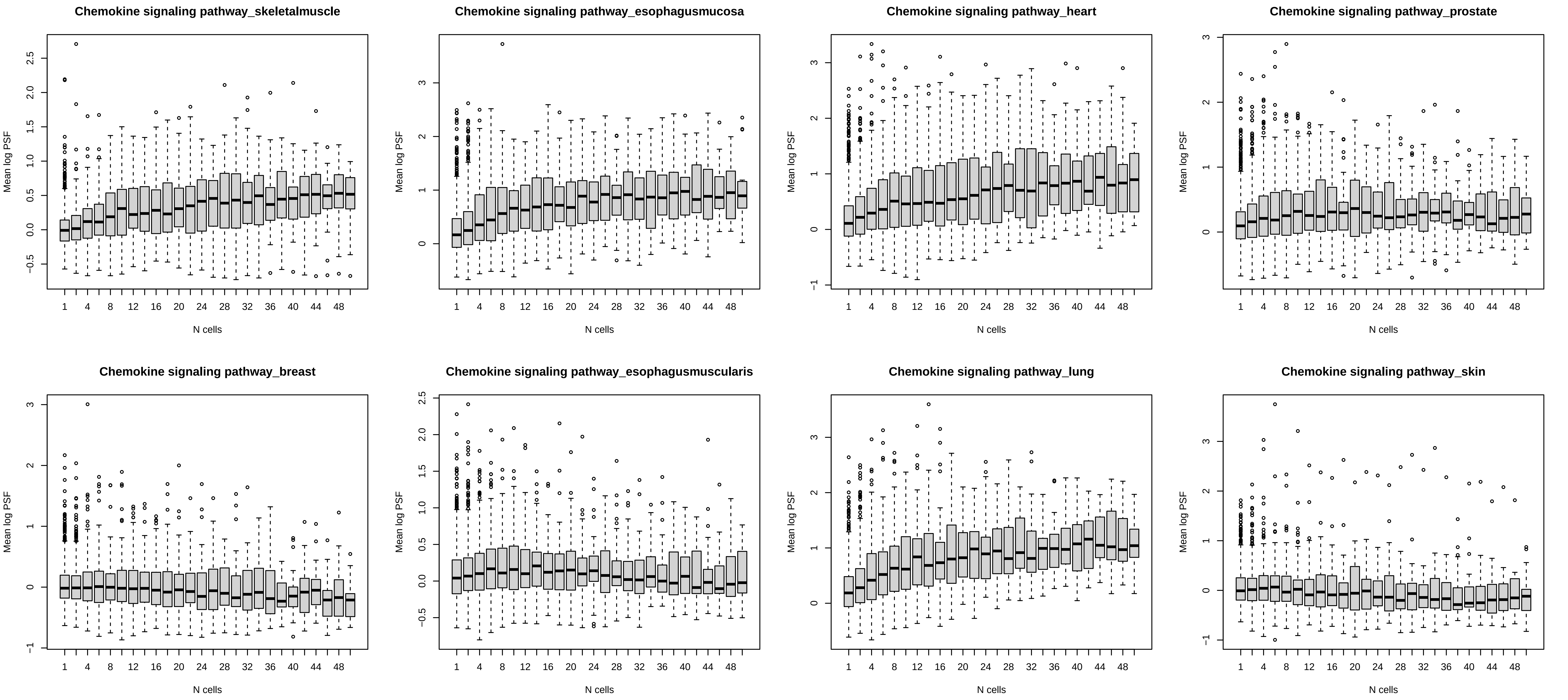
Chemokine signaling pathway_skeletalmuscle
Chemokine signaling pathway_esophagusmucosa
Chemokine signaling pathway_heart
Chemokine signaling pathway_prostate
Chemokine signaling pathway_breast
Chemokine signaling pathway_esophagusmuscularis
Chemokine signaling pathway_lung
Chemokine signaling pathway_skin
Mean log PSF
N cells

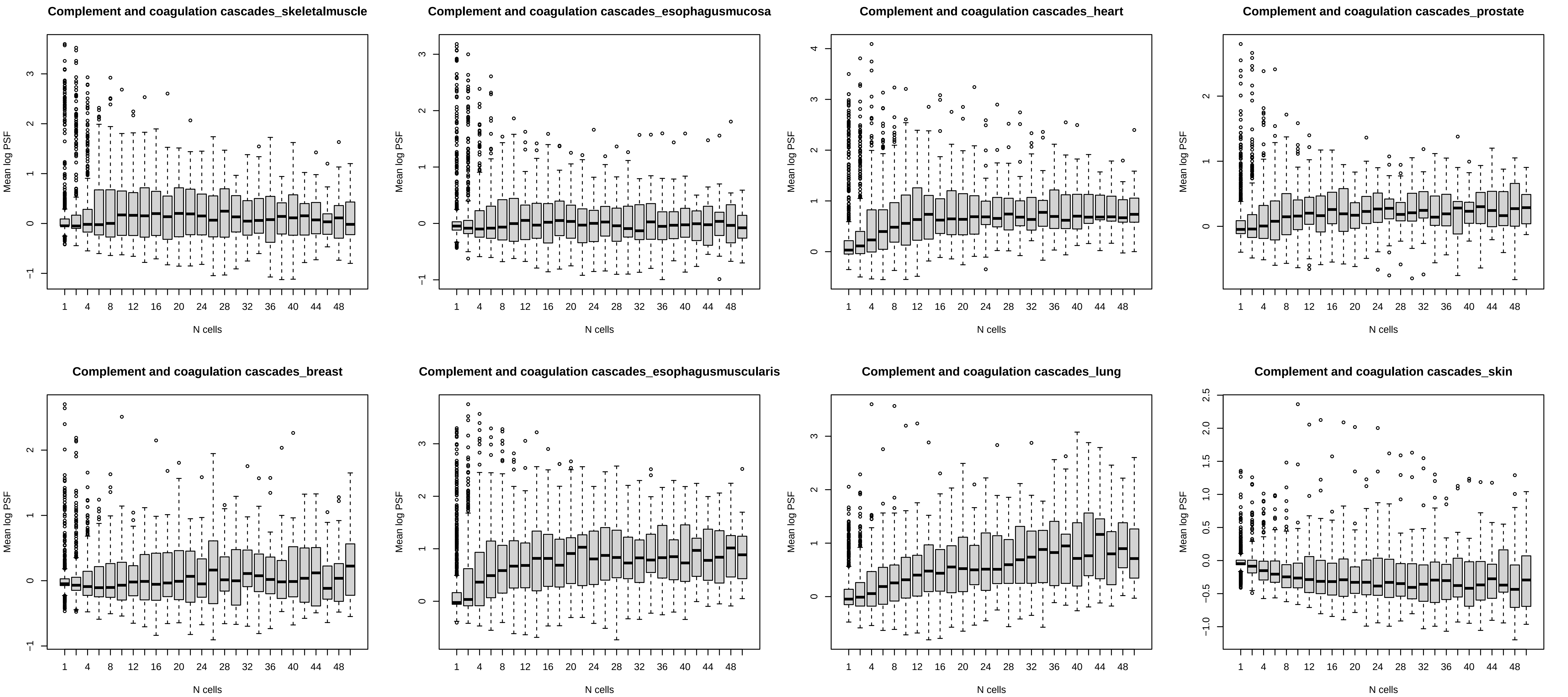
Complement and coagulation cascades_skeletalmuscle
Complement and coagulation cascades_esophagusmucosa
Complement and coagulation cascades_heart
Complement and coagulation cascades_prostate
Complement and coagulation cascades_breast
Complement and coagulation cascades_esophagusmuscularis
Complement and coagulation cascades_lung
Complement and coagulation cascades_skin
Mean log PSF
N cells
1 4 8 12 16 20 24 28 32 36 40 44 48

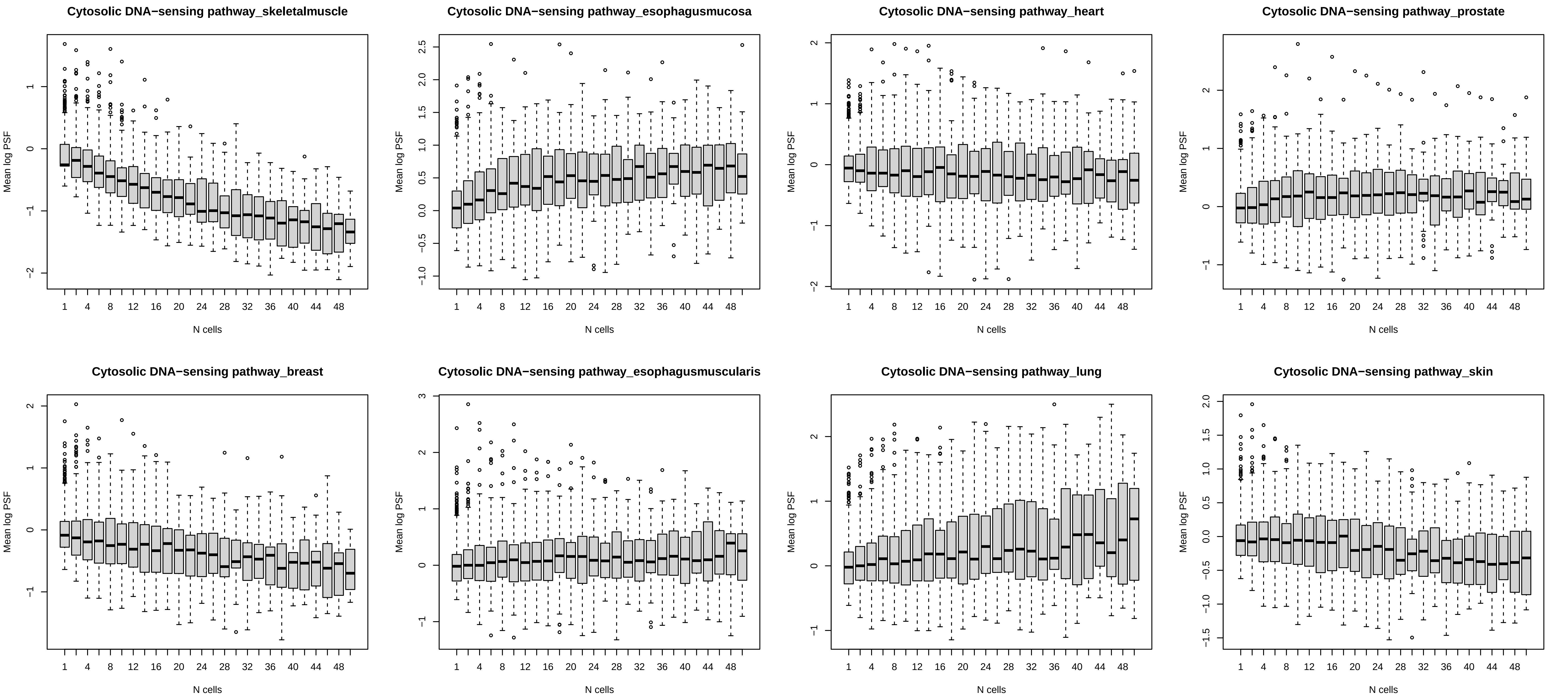

Cytosolic DNA−sensing pathway_skeletalmuscle
Cytosolic DNA−sensing pathway_esophagusmucosa
Cytosolic DNA−sensing pathway_heart
Cytosolic DNA−sensing pathway_prostate
Cytosolic DNA−sensing pathway_breast
Cytosolic DNA−sensing pathway_esophagusmuscularis
Cytosolic DNA−sensing pathway_lung
Cytosolic DNA−sensing pathway_skin
Mean log PSF
N cells
1 4 8 12 16 20 24 28 32 36 40 44 48

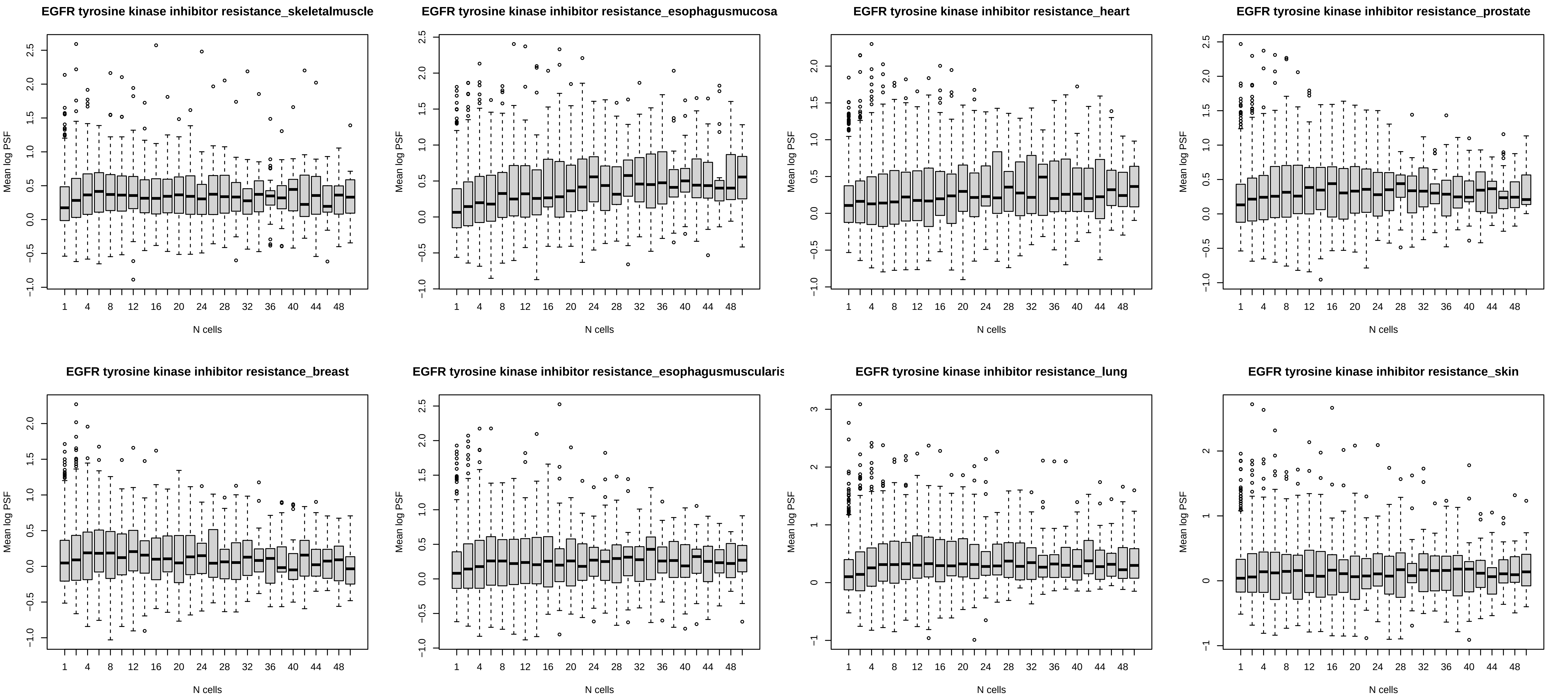

EGFR tyrosine kinase inhibitor resistance_skeletalmuscle
EGFR tyrosine kinase inhibitor resistance_esophagusmucosa
EGFR tyrosine kinase inhibitor resistance_heart
EGFR tyrosine kinase inhibitor resistance_prostate
EGFR tyrosine kinase inhibitor resistance_breast
EGFR tyrosine kinase inhibitor resistance_esophagusmuscularis
EGFR tyrosine kinase inhibitor resistance_lung
EGFR tyrosine kinase inhibitor resistance_skin
Mean log PSF
N cells

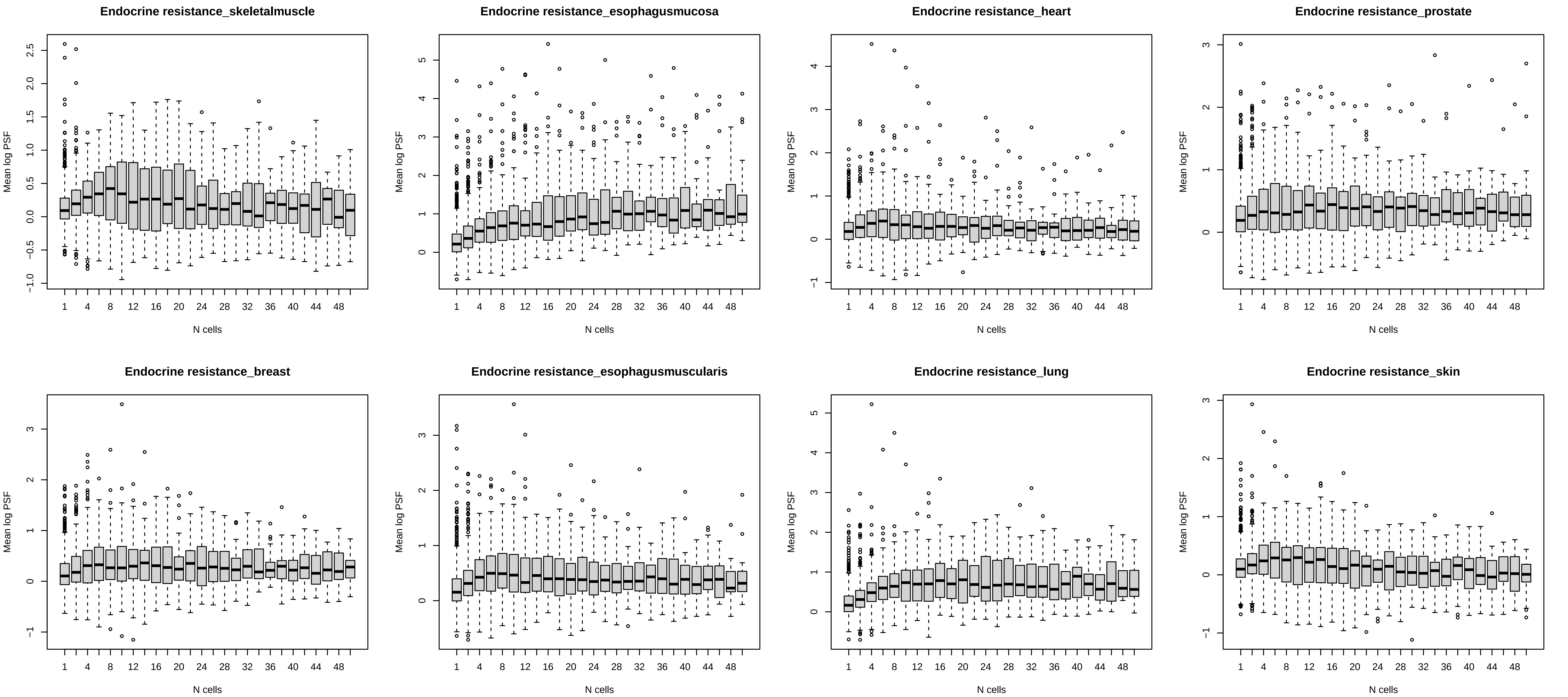
Endocrine resistance_skeletalmuscle
Endocrine resistance_esophagusmucosa
Endocrine resistance_heart
Endocrine resistance_prostate
Endocrine resistance_breast
Endocrine resistance_esophagusmuscularis
Endocrine resistance_lung
Endocrine resistance_skin
Mean log PSF
N cells
1 4 8 12 16 20 24 28 32 36 40 44 48

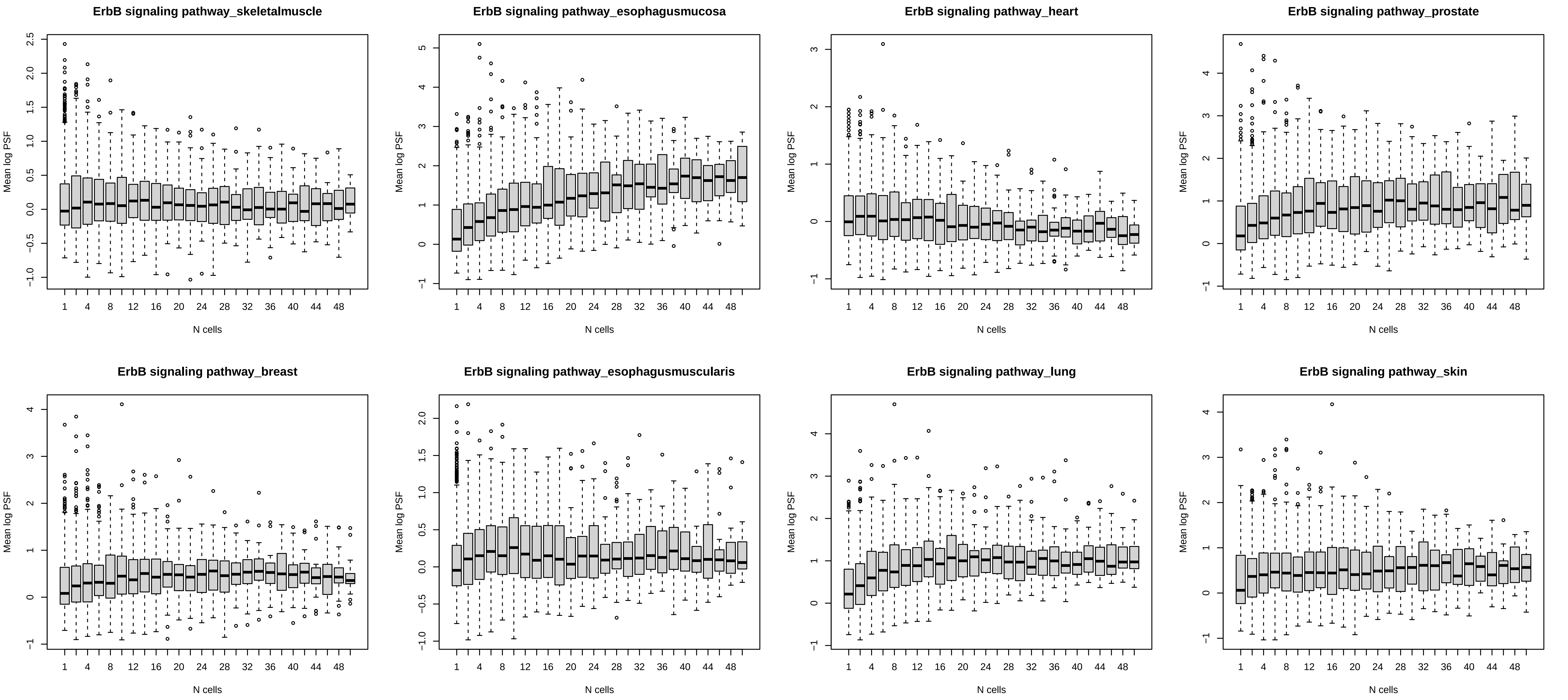
ErbB signaling pathway_skeletalmuscle
ErbB signaling pathway_esophagusmucosa
ErbB signaling pathway_heart
ErbB signaling pathway_prostate
ErbB signaling pathway_breast
ErbB signaling pathway_esophagusmuscularis
ErbB signaling pathway_lung
ErbB signaling pathway_skin
Mean log PSF
N cells

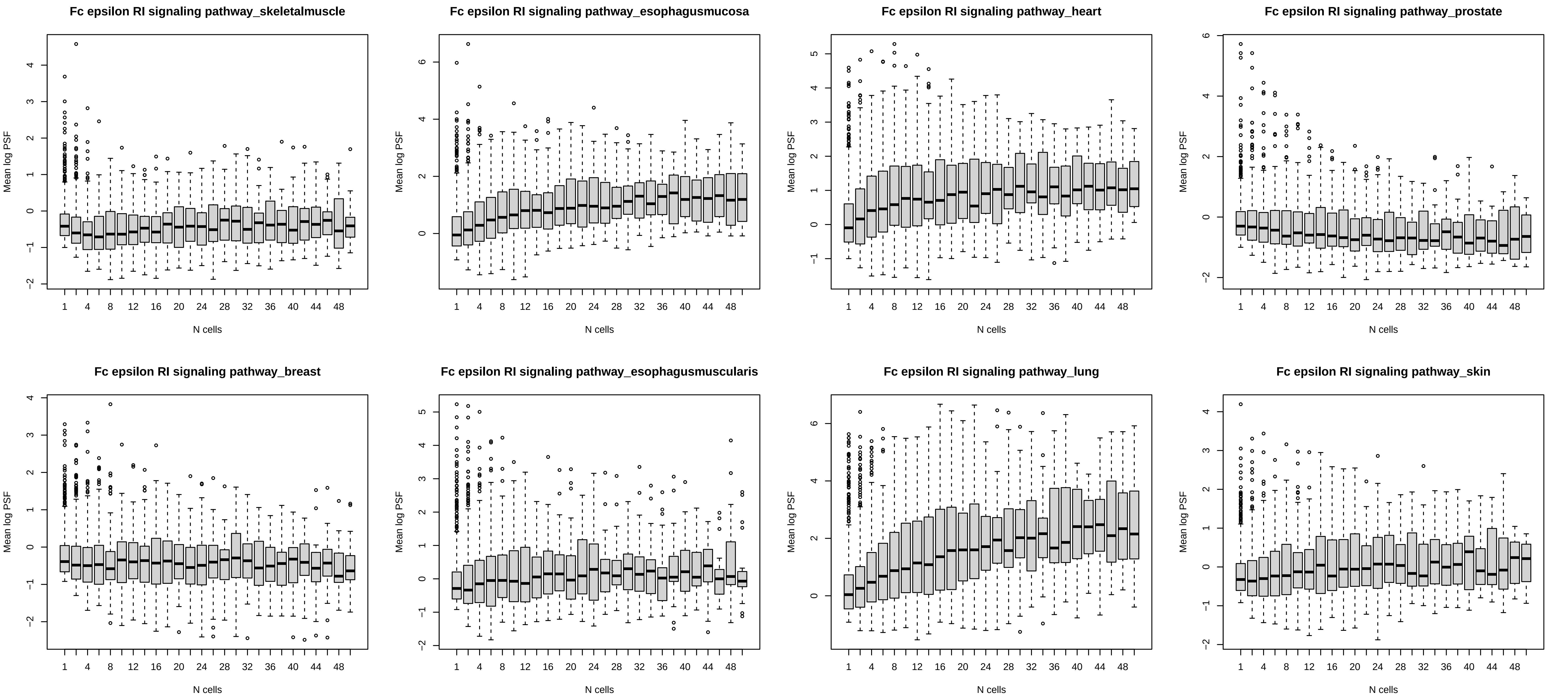
Fc epsilon RI signaling pathway_skeletalmuscle
Fc epsilon RI signaling pathway_esophagusmucosa
Fc epsilon RI signaling pathway_heart
Fc epsilon RI signaling pathway_prostate
Fc epsilon RI signaling pathway_breast
Fc epsilon RI signaling pathway_esophagusmuscularis
Fc epsilon RI signaling pathway_lung
Fc epsilon RI signaling pathway_skin
Mean log PSF
N cells
1 4 8 12 16 20 24 28 32 36 40 44 48

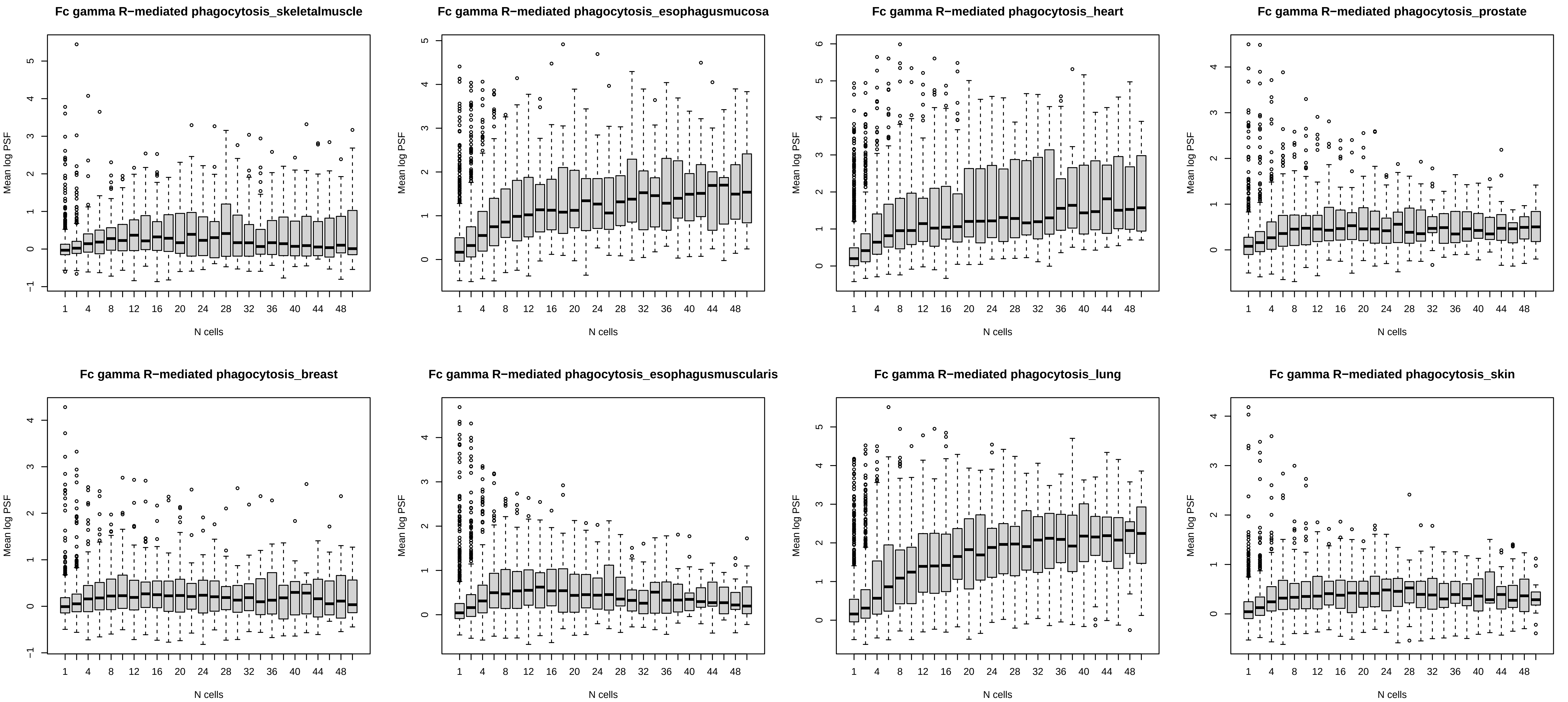
Fc gamma R−mediated phagocytosis_skeletalmuscle
Fc gamma R−mediated phagocytosis_esophagusmucosa
Fc gamma R−mediated phagocytosis_heart
Fc gamma R−mediated phagocytosis_prostate
Fc gamma R−mediated phagocytosis_breast
Fc gamma R−mediated phagocytosis_esophagusmuscularis
Fc gamma R−mediated phagocytosis_lung
Fc gamma R−mediated phagocytosis_skin
Mean log PSF
N cells
1 4 8 12 16 20 24 28 32 36 40 44 48

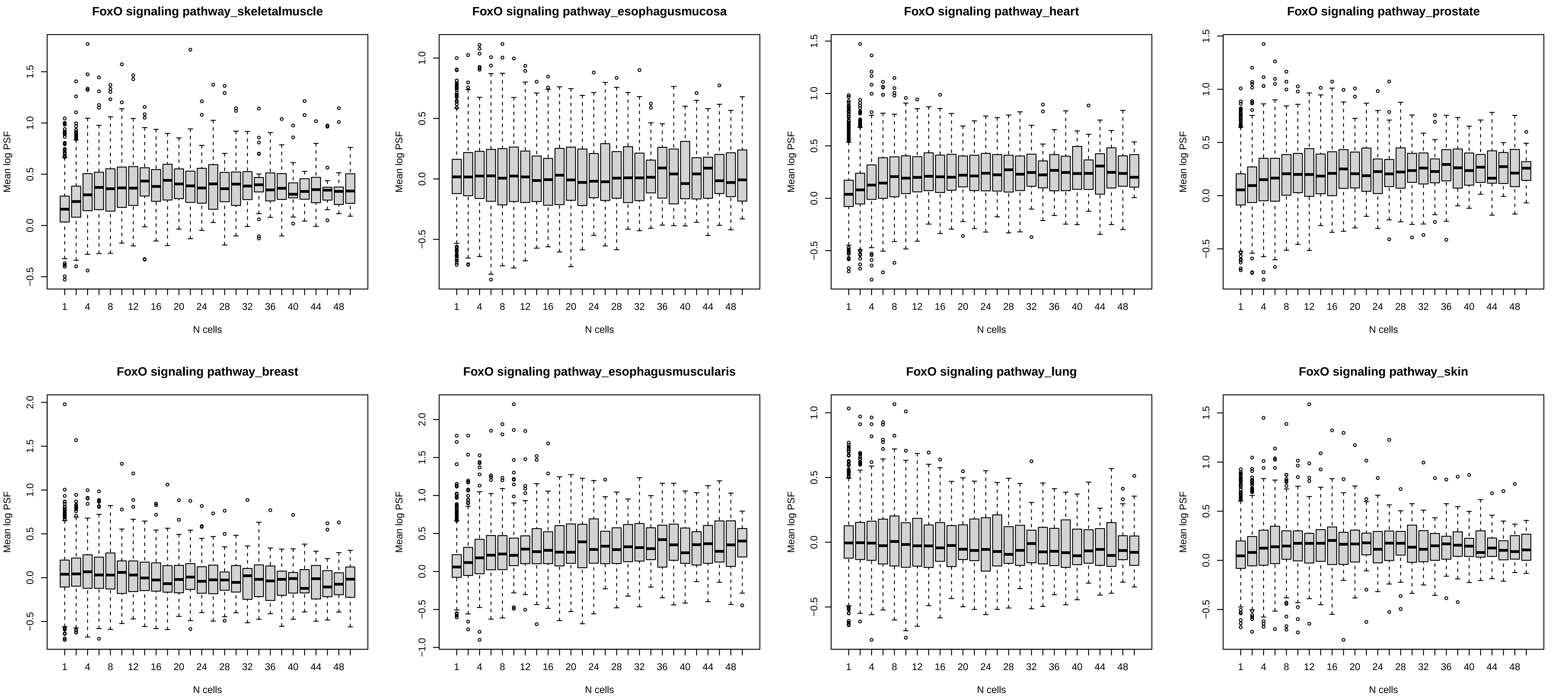
FoxO signaling pathway_skeletalmuscle
FoxO signaling pathway_esophagusmucosa
FoxO signaling pathway_heart
FoxO signaling pathway_prostate
FoxO signaling pathway_breast
FoxO signaling pathway_esophagusmuscularis
FoxO signaling pathway_lung
FoxO signaling pathway_skin
Mean log PSF
N cells

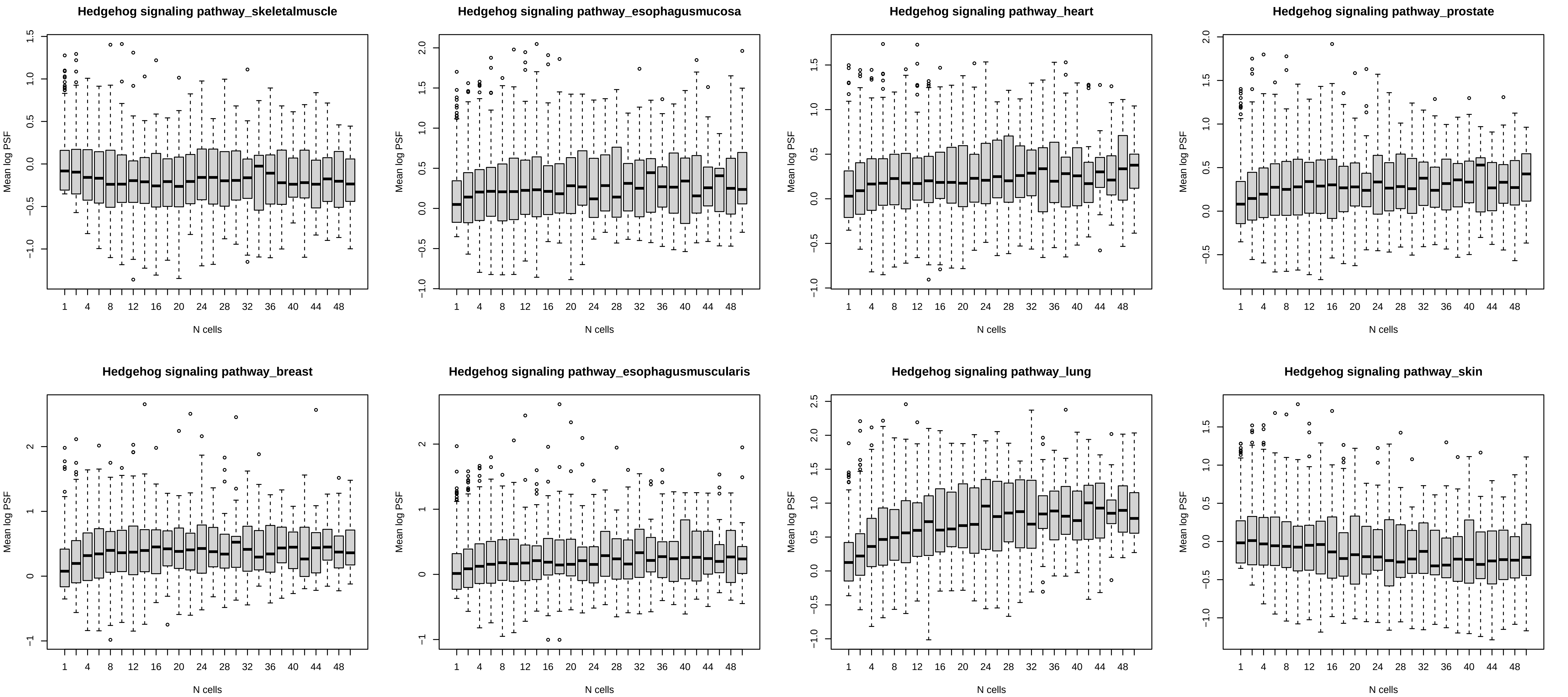
Hedgehog signaling pathway_skeletalmuscle
Hedgehog signaling pathway_esophagusmucosa
Hedgehog signaling pathway_heart
Hedgehog signaling pathway_prostate
Hedgehog signaling pathway_breast
Hedgehog signaling pathway_esophagusmuscularis
Hedgehog signaling pathway_lung
Hedgehog signaling pathway_skin
Mean log PSF
N cells

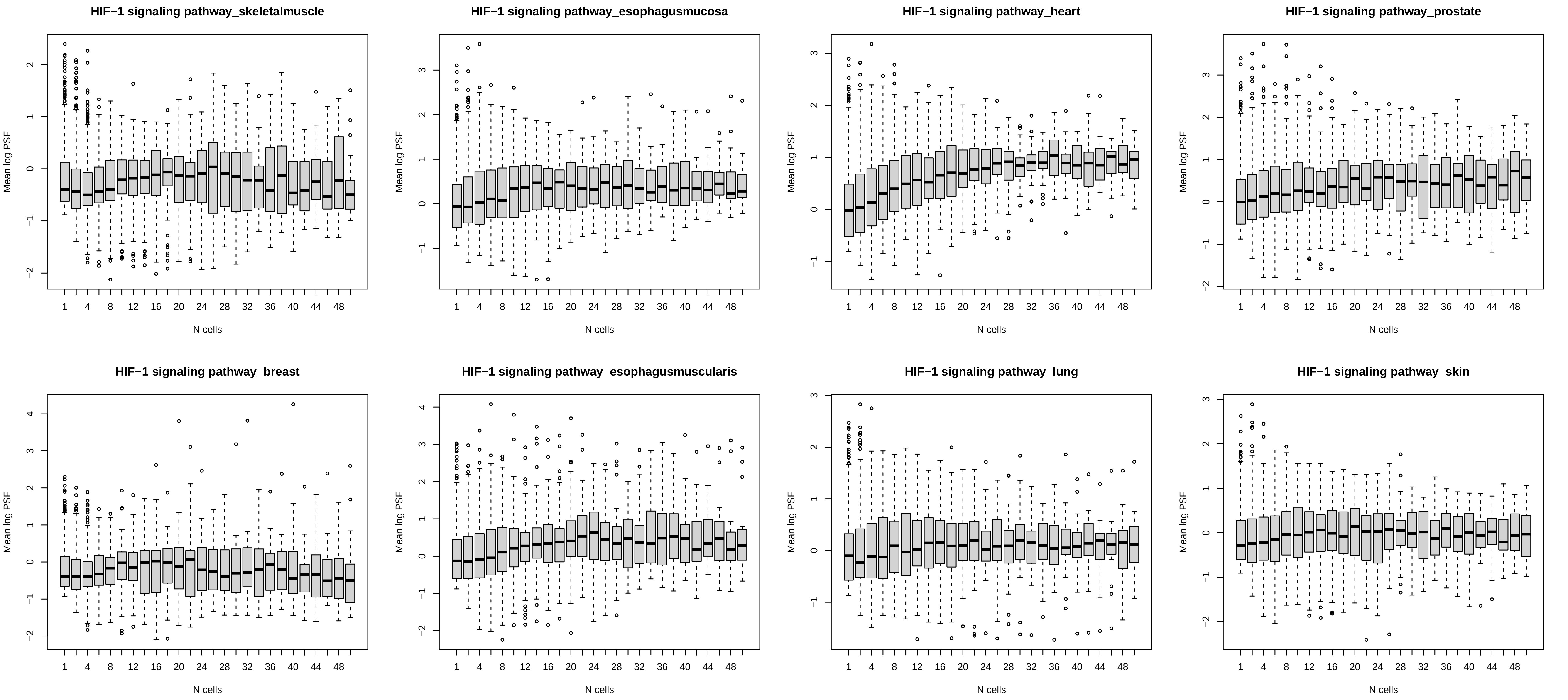
HIF−1 signaling pathway_skeletalmuscle
HIF−1 signaling pathway_esophagusmucosa
HIF−1 signaling pathway_heart
HIF−1 signaling pathway_prostate
HIF−1 signaling pathway_breast
HIF−1 signaling pathway_esophagusmuscularis
HIF−1 signaling pathway_lung
HIF−1 signaling pathway_skin
Mean log PSF
N cells

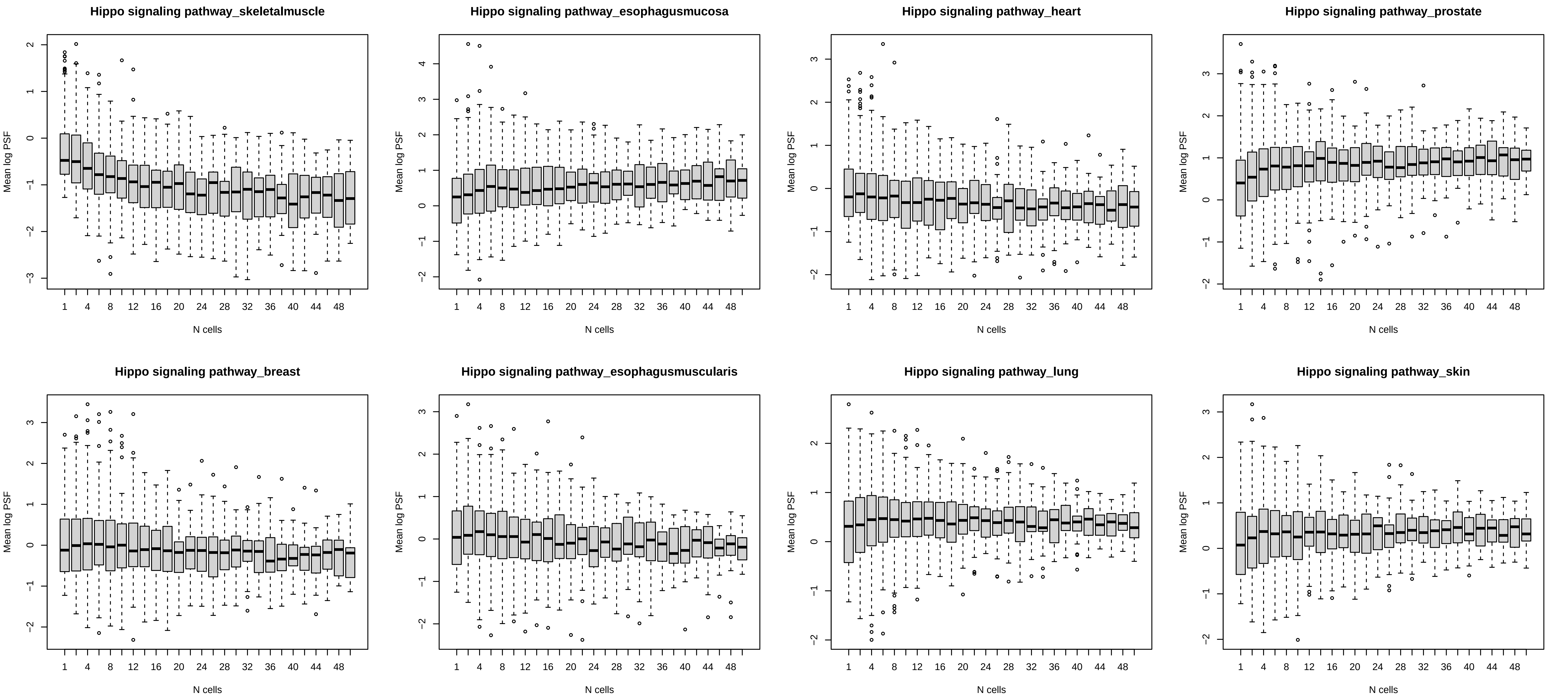
Hippo signaling pathway_skeletalmuscle
Hippo signaling pathway_esophagusmucosa
Hippo signaling pathway_heart
Hippo signaling pathway_prostate
Hippo signaling pathway_breast
Hippo signaling pathway_esophagusmuscularis
Hippo signaling pathway_lung
Hippo signaling pathway_skin
Mean log PSF
N cells

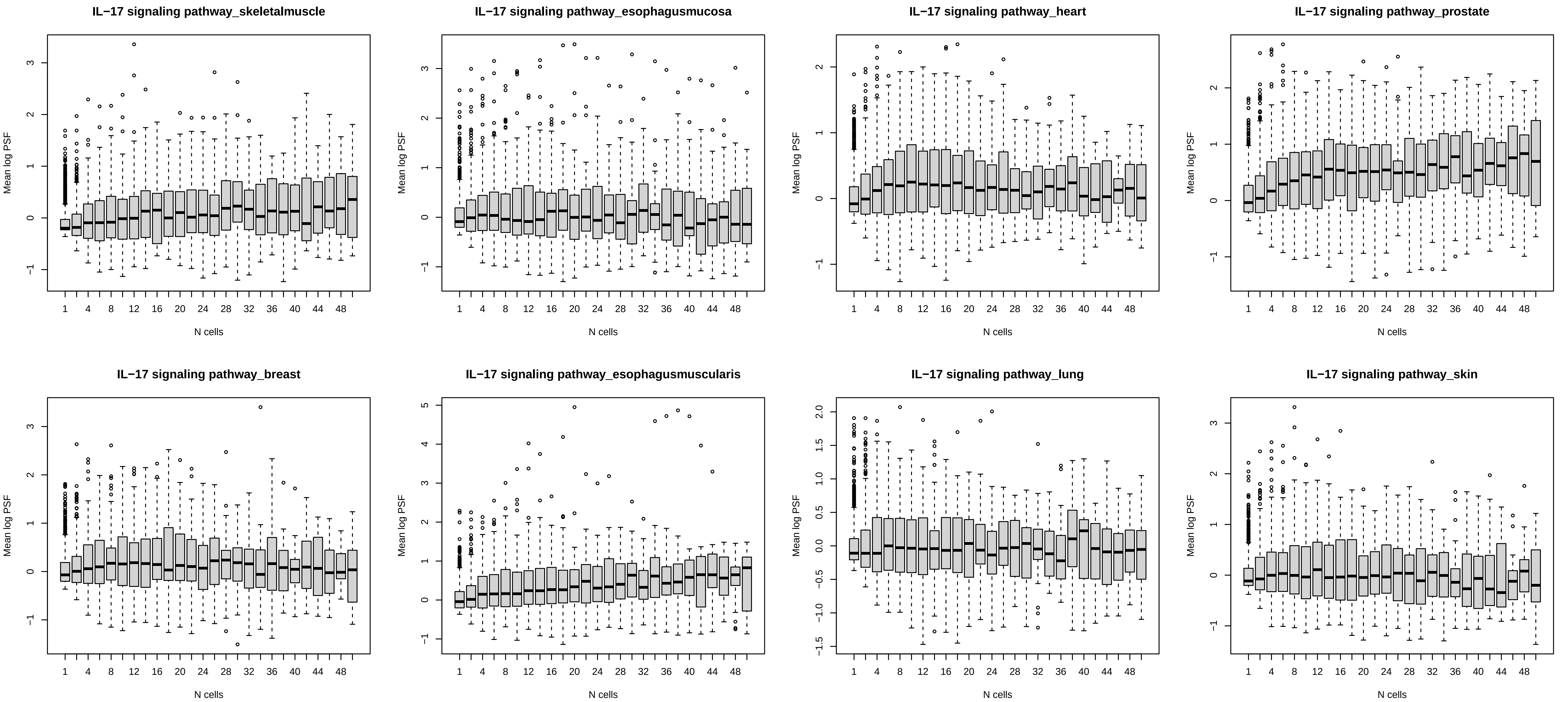

IL−17 signaling pathway_skeletalmuscle
IL−17 signaling pathway_esophagusmucosa
IL−17 signaling pathway_heart
IL−17 signaling pathway_prostate
IL−17 signaling pathway_breast
IL−17 signaling pathway_esophagusmuscularis
IL−17 signaling pathway_lung
IL−17 signaling pathway_skin
Mean log PSF
N cells

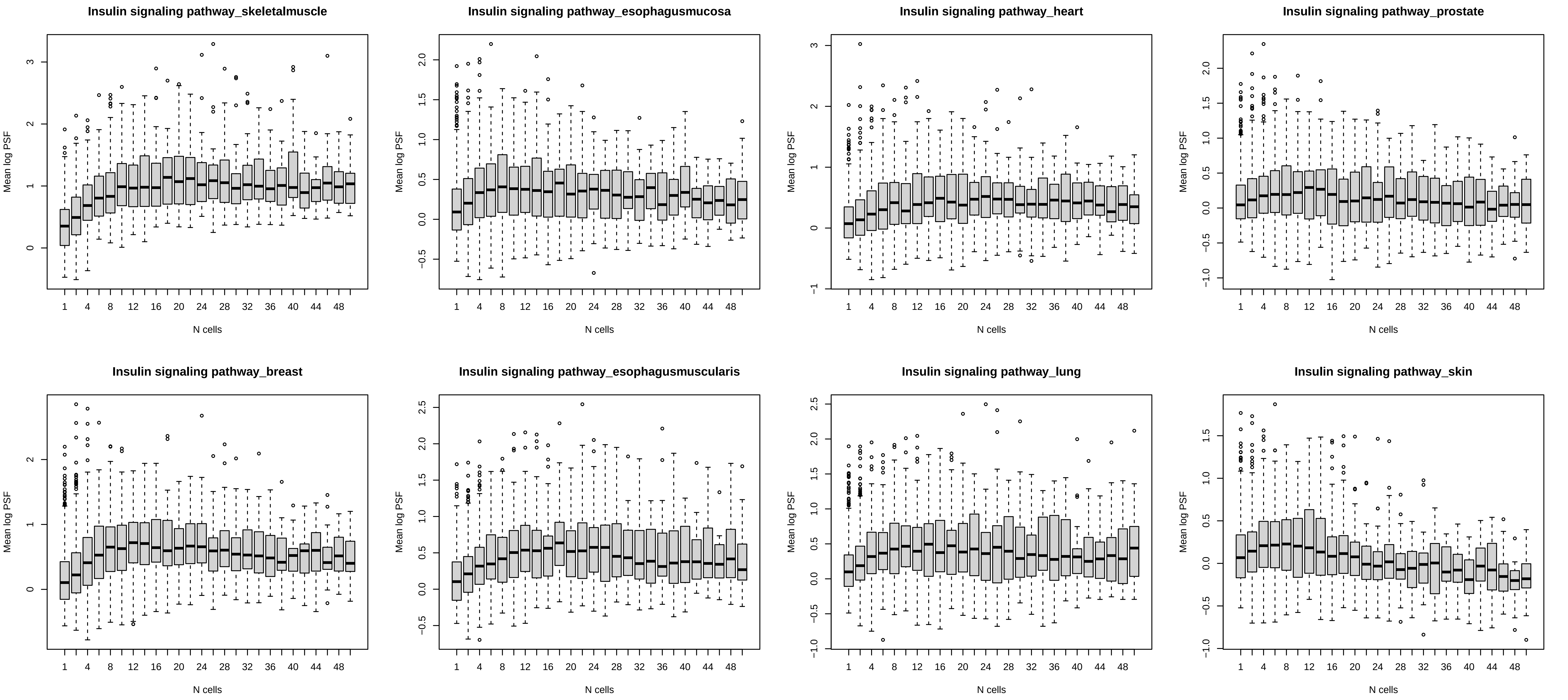

Insulin signaling pathway_skeletalmuscle
Insulin signaling pathway_esophagusmucosa
Insulin signaling pathway_heart
Insulin signaling pathway_prostate
Insulin signaling pathway_breast
Insulin signaling pathway_esophagusmuscularis
Insulin signaling pathway_lung
Insulin signaling pathway_skin
Mean log PSF
N cells
1 4 8 12 16 20 24 28 32 36 40 44 48

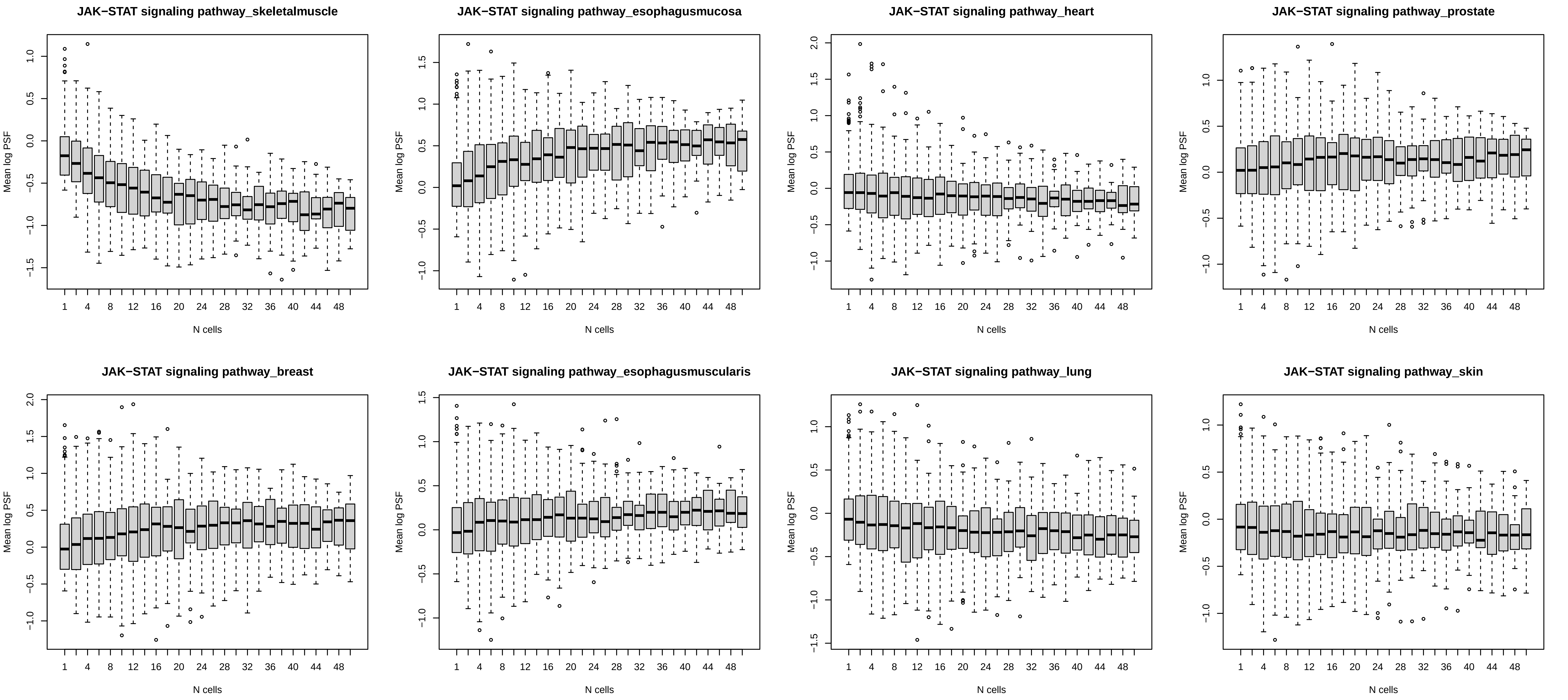
JAK−STAT signaling pathway_skeletalmuscle
JAK−STAT signaling pathway_esophagusmucosa
JAK−STAT signaling pathway_heart
JAK−STAT signaling pathway_prostate
JAK−STAT signaling pathway_breast
JAK−STAT signaling pathway_esophagusmuscularis
JAK−STAT signaling pathway_lung
JAK−STAT signaling pathway_skin
Mean log PSF
N cells
1 4 8 12 16 20 24 28 32 36 40 44 48

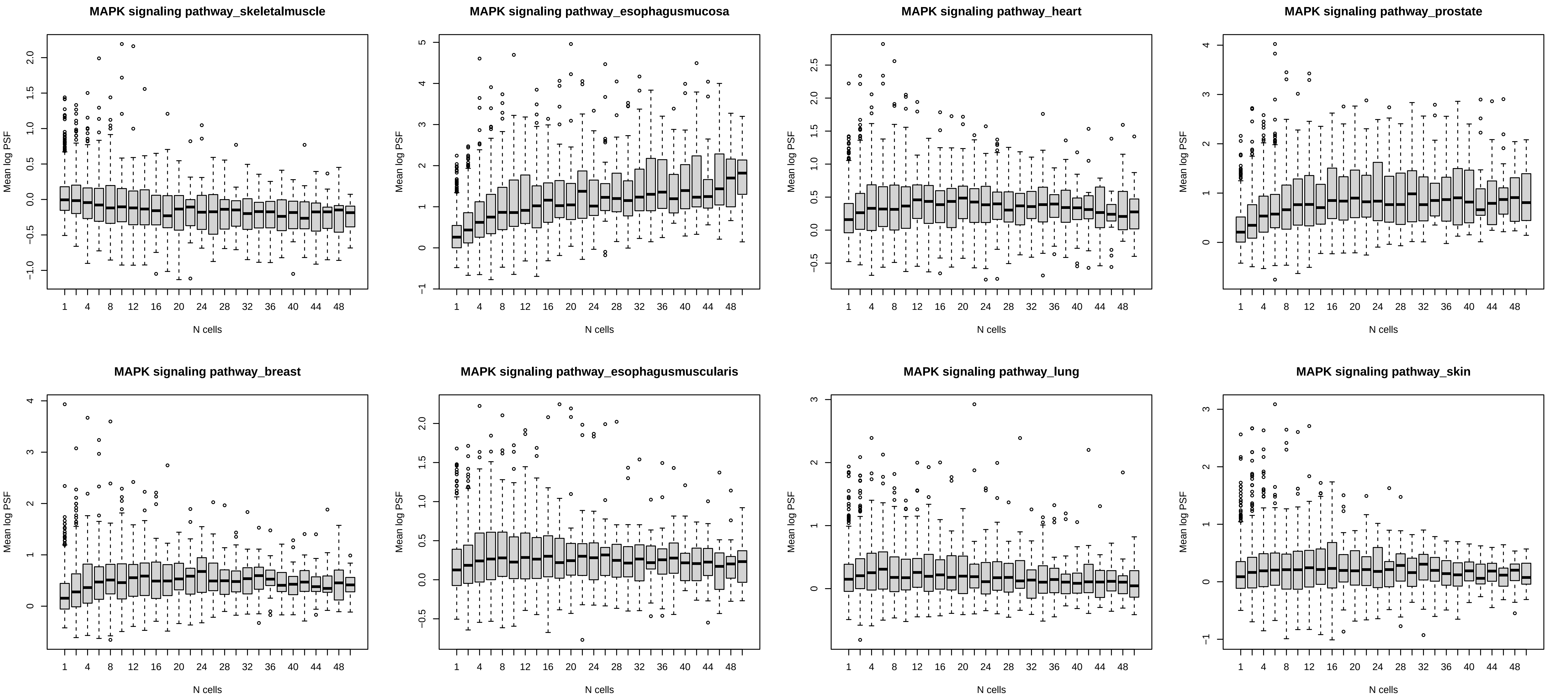
MAPK signaling pathway_skeletalmuscle
MAPK signaling pathway_esophagusmucosa
MAPK signaling pathway_heart
MAPK signaling pathway_prostate
MAPK signaling pathway_breast
MAPK signaling pathway_esophagusmuscularis
MAPK signaling pathway_lung
MAPK signaling pathway_skin
Mean log PSF
N cells
1 4 8 12 16 20 24 28 32 36 40 44 48

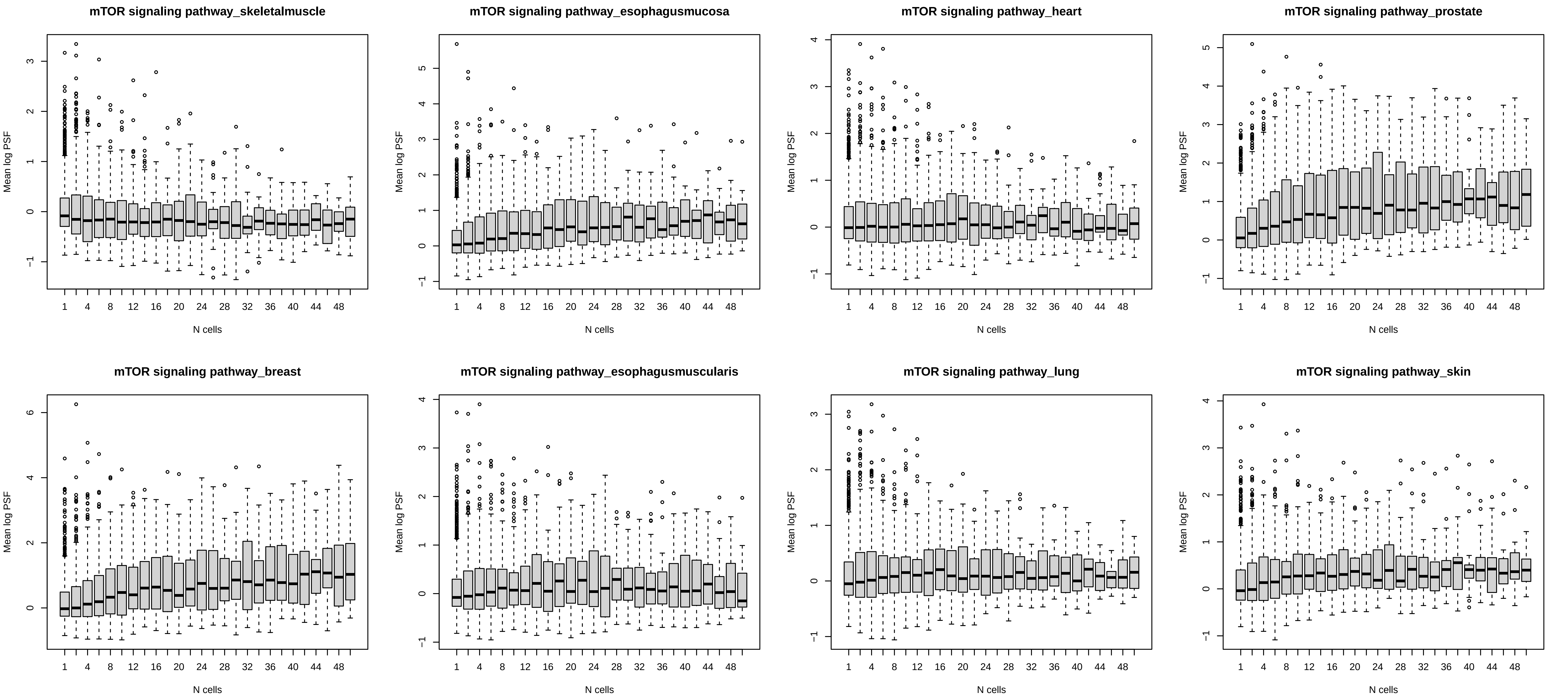
mTOR signaling pathway_skeletalmuscle
mTOR signaling pathway_esophagusmucosa
mTOR signaling pathway_heart
mTOR signaling pathway_prostate
mTOR signaling pathway_breast
mTOR signaling pathway_esophagusmuscularis
mTOR signaling pathway_lung
mTOR signaling pathway_skin
Mean log PSF
N cells
1
4
8
12
16
20
24
28
32
36
40
44
48

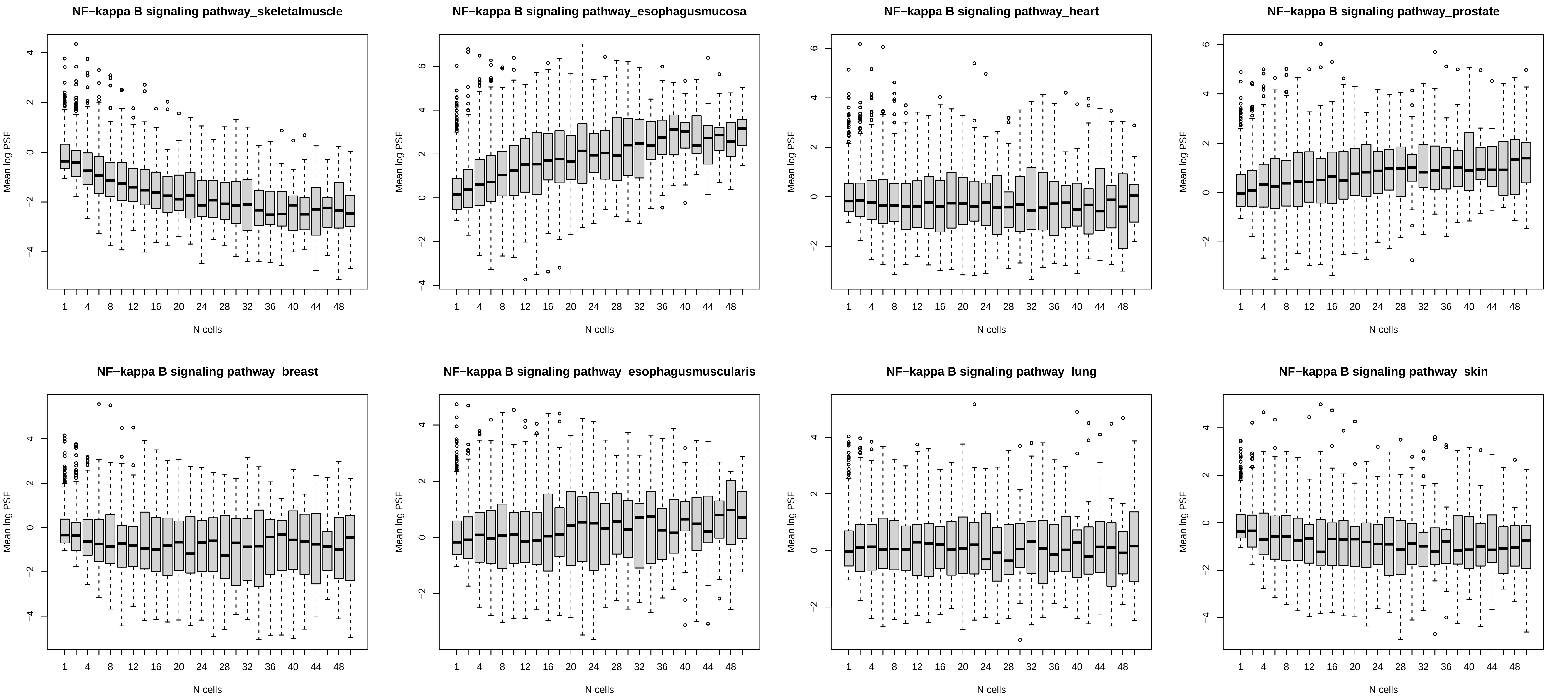
NF−kappa B signaling pathway_skeletalmuscle
NF−kappa B signaling pathway_esophagusmucosa
NF−kappa B signaling pathway_heart
NF−kappa B signaling pathway_prostate
NF−kappa B signaling pathway_breast
NF−kappa B signaling pathway_esophagusmuscularis
NF−kappa B signaling pathway_lung
NF−kappa B signaling pathway_skin
Mean log PSF
N cells
1 4 8 12 16 20 24 28 32 36 40 44 48

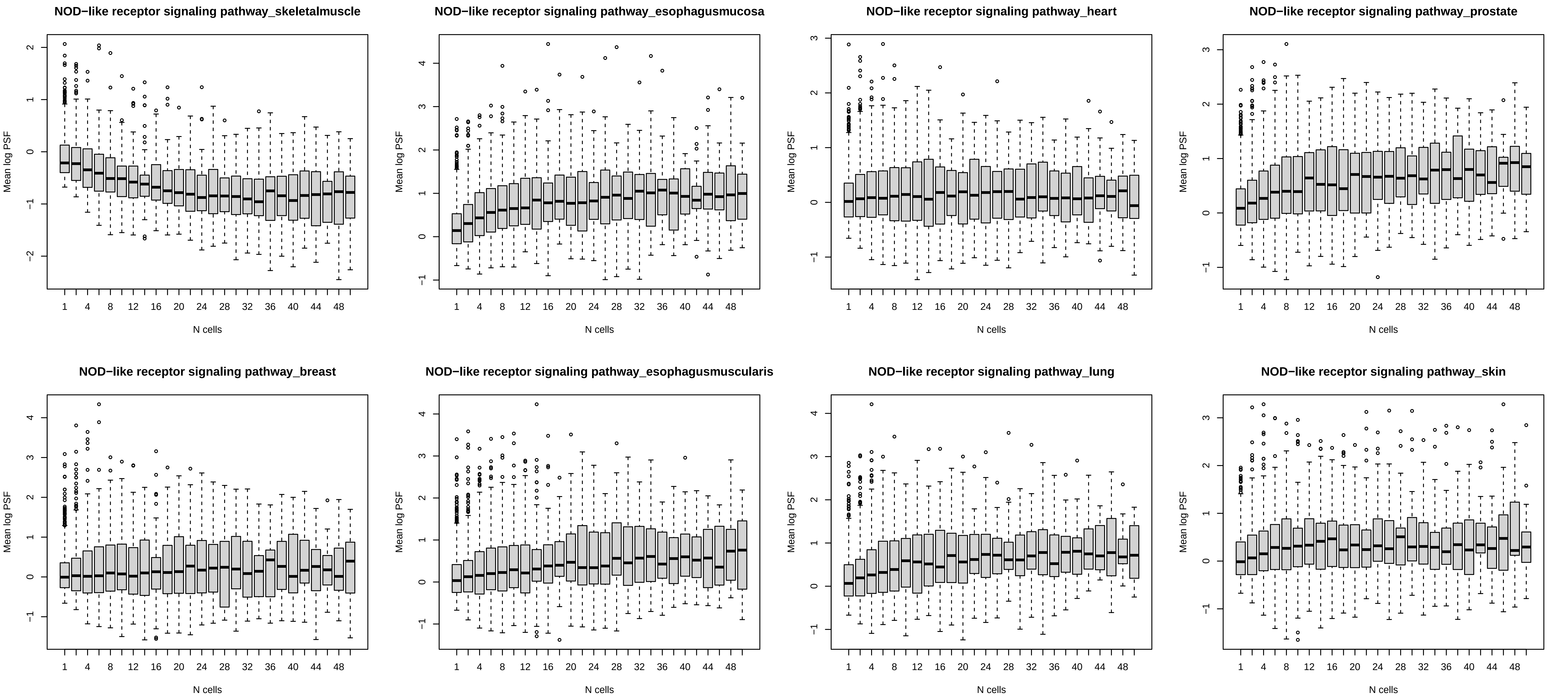
NOD−like receptor signaling pathway_skeletalmuscle
NOD−like receptor signaling pathway_esophagusmucosa
NOD−like receptor signaling pathway_heart
NOD−like receptor signaling pathway_prostate
NOD−like receptor signaling pathway_breast
NOD−like receptor signaling pathway_esophagusmuscularis
NOD−like receptor signaling pathway_lung
NOD−like receptor signaling pathway_skin
Mean log PSF
N cells

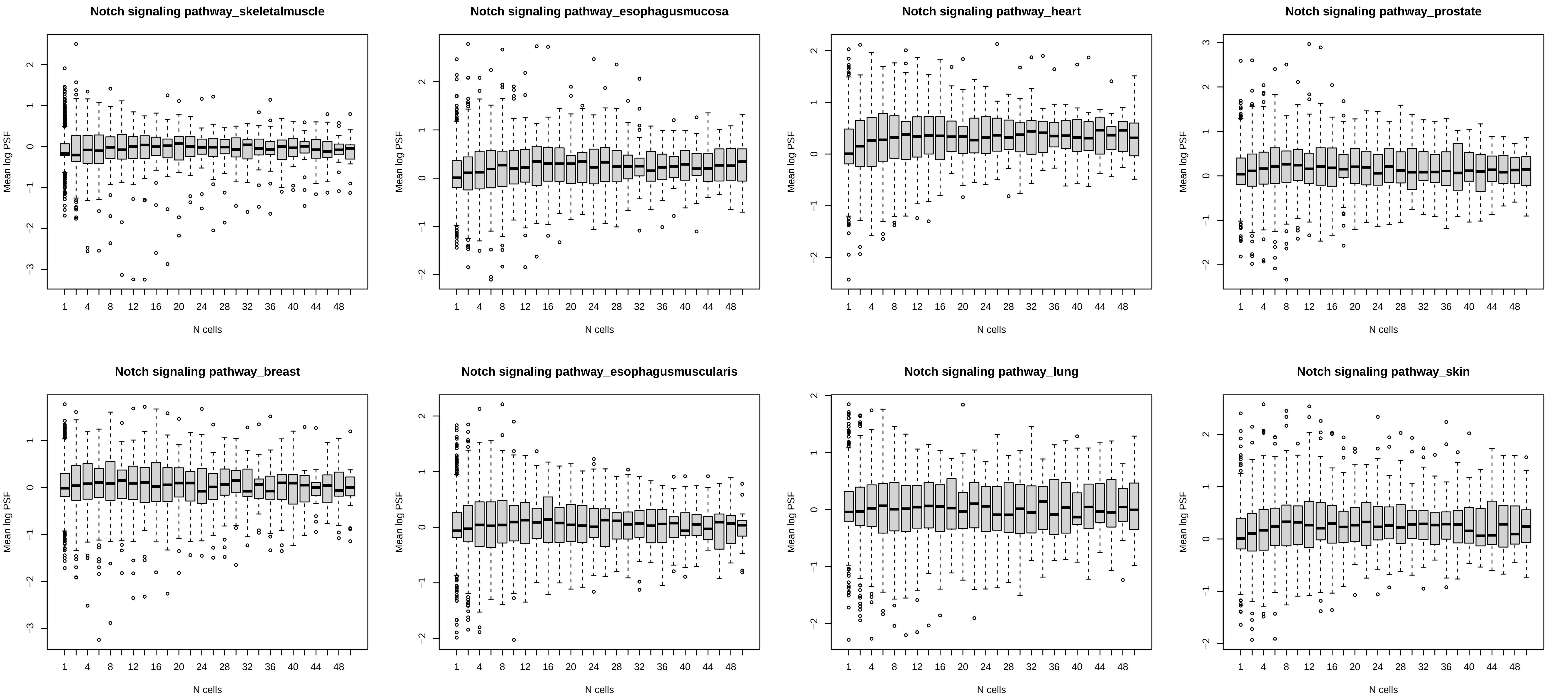
Notch signaling pathway_skeletalmuscle
Notch signaling pathway_esophagusmucosa
Notch signaling pathway_heart
Notch signaling pathway_prostate
Notch signaling pathway_breast
Notch signaling pathway_esophagusmuscularis
Notch signaling pathway_lung
Notch signaling pathway_skin
Mean log PSF
N cells
1
4
8
12
16
20
24
28
32
36
40
44
48

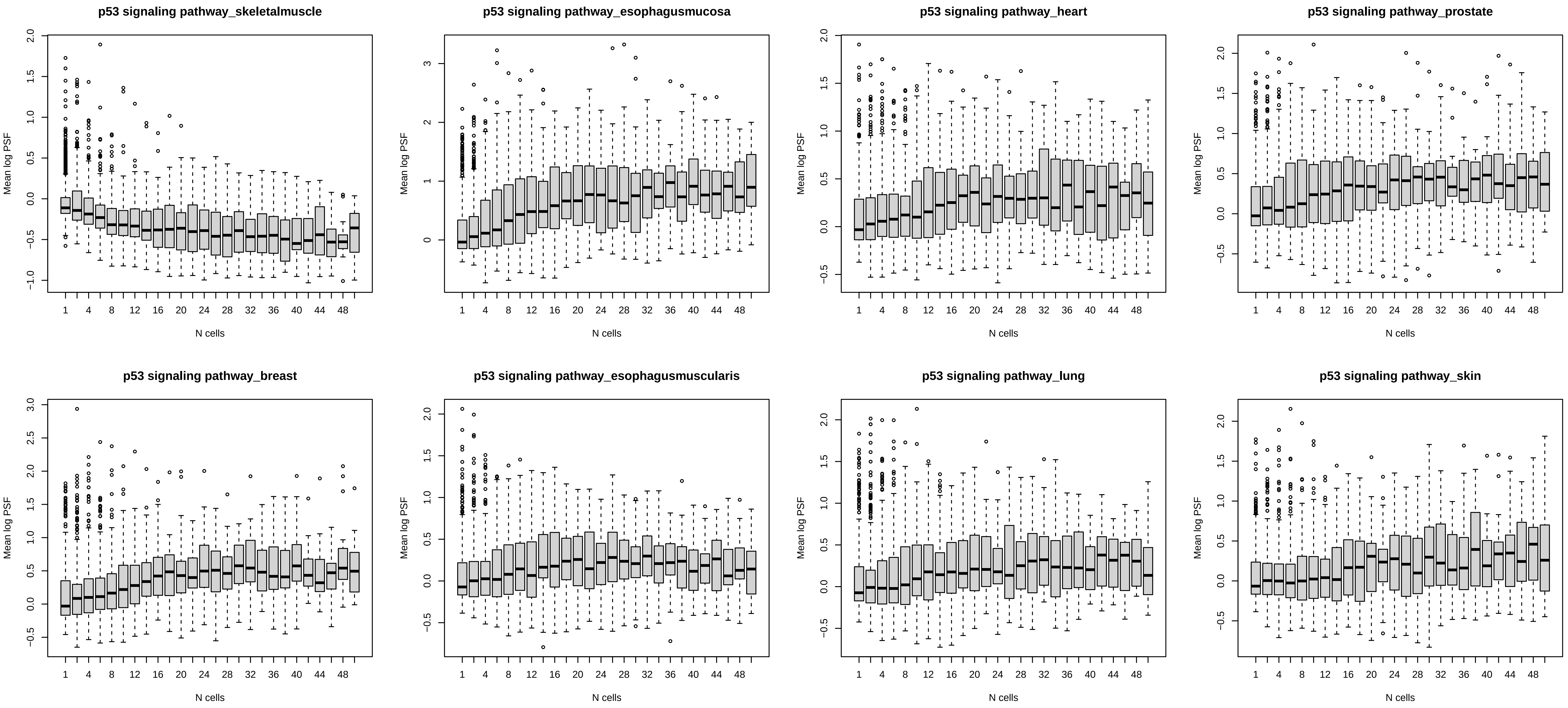
p53 signaling pathway_skeletalmuscle
p53 signaling pathway_esophagusmucosa
p53 signaling pathway_heart
p53 signaling pathway_prostate
p53 signaling pathway_breast
p53 signaling pathway_esophagusmuscularis
p53 signaling pathway_lung
p53 signaling pathway_skin
Mean log PSF
N cells
1 4 8 12 16 20 24 28 32 36 40 44 48

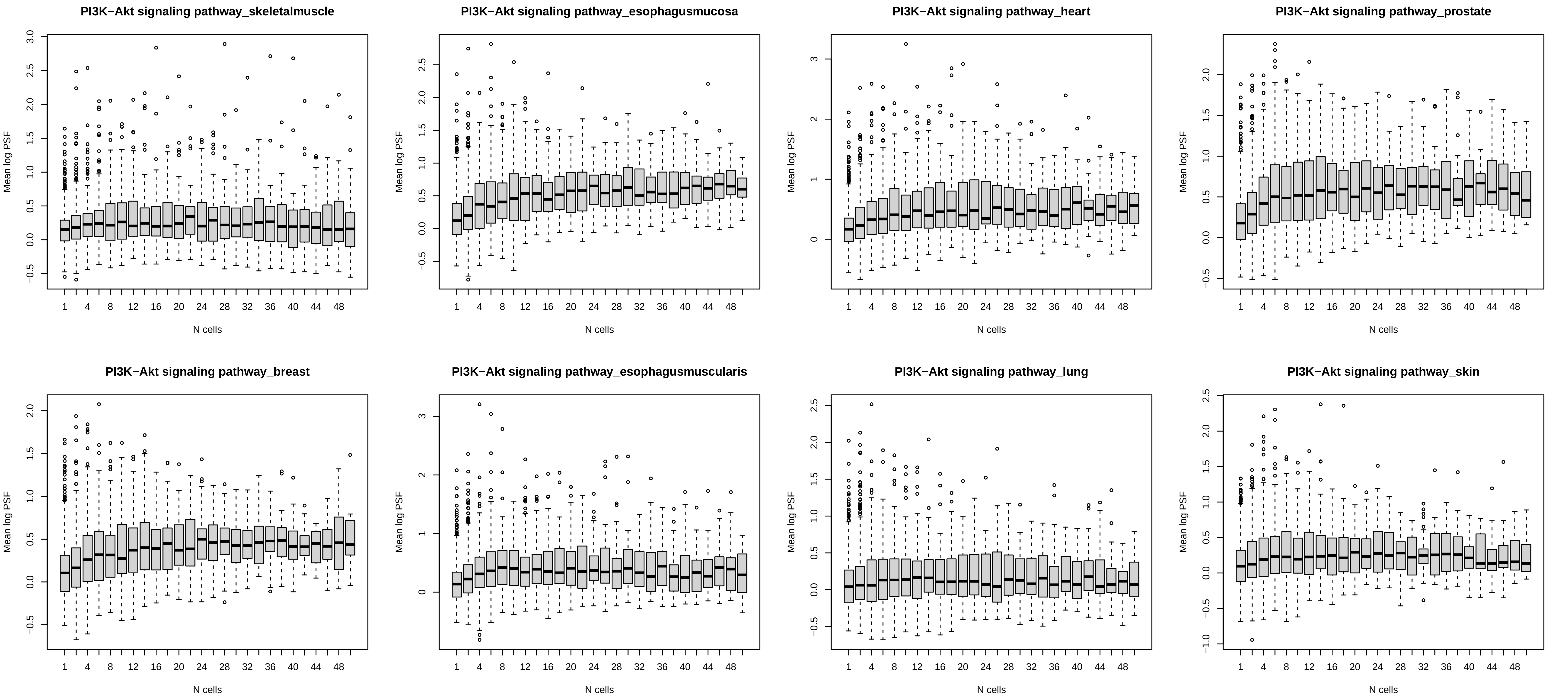
PI3K−Akt signaling pathway_skeletalmuscle
PI3K−Akt signaling pathway_esophagusmucosa
PI3K−Akt signaling pathway_heart
PI3K−Akt signaling pathway_prostate
PI3K−Akt signaling pathway_breast
PI3K−Akt signaling pathway_esophagusmuscularis
PI3K−Akt signaling pathway_lung
PI3K−Akt signaling pathway_skin
Mean log PSF
N cells
1
4
8
12
16
20
24
28
32
36
40
44
48

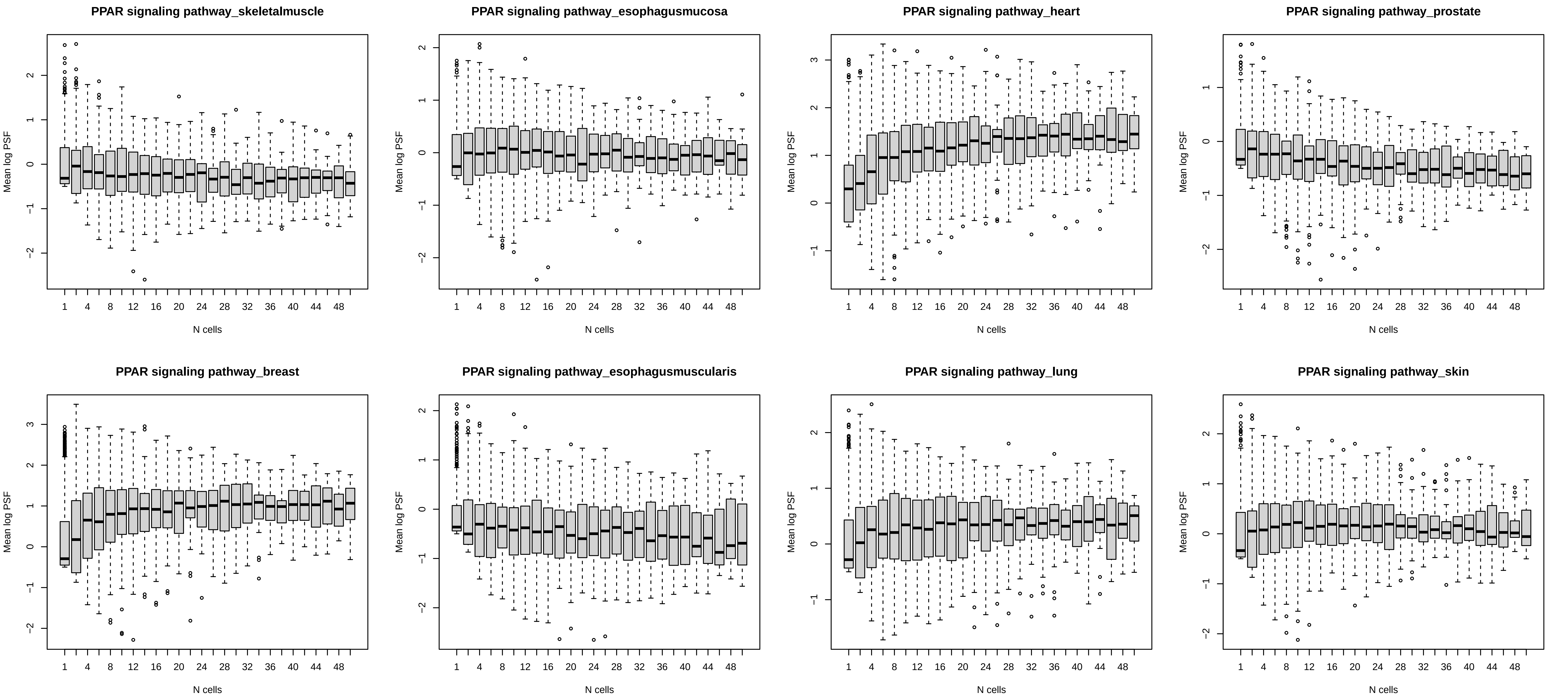
PPAR signaling pathway_skeletalmuscle
PPAR signaling pathway_esophagusmucosa
PPAR signaling pathway_heart
PPAR signaling pathway_prostate
PPAR signaling pathway_breast
PPAR signaling pathway_esophagusmuscularis
PPAR signaling pathway_lung
PPAR signaling pathway_skin
Mean log PSF
N cells
1 4 8 12 16 20 24 28 32 36 40 44 48

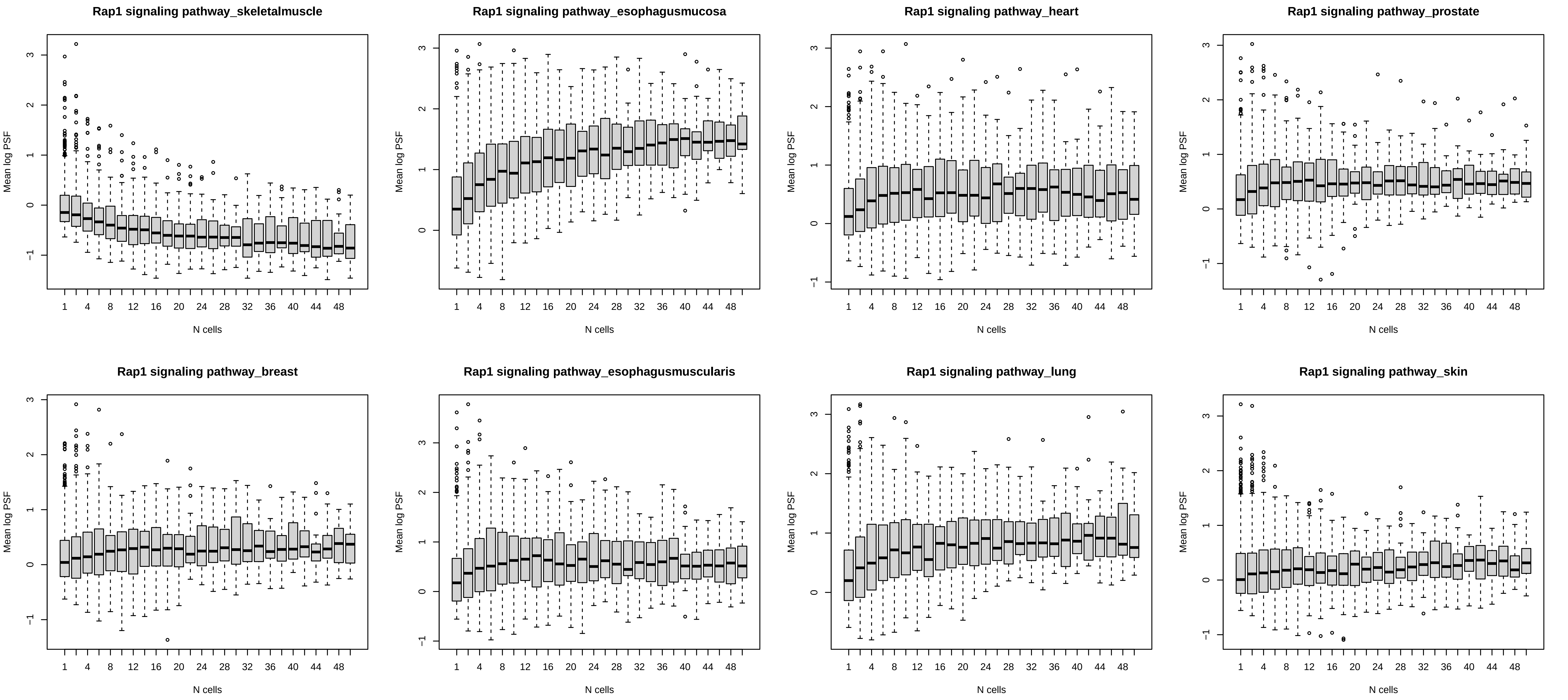

Rap1 signaling pathway_skeletalmuscle
Rap1 signaling pathway_esophagusmucosa
Rap1 signaling pathway_heart
Rap1 signaling pathway_prostate
Rap1 signaling pathway_breast
Rap1 signaling pathway_esophagusmuscularis
Rap1 signaling pathway_lung
Rap1 signaling pathway_skin
Mean log PSF
N cells
1 4 8 12 16 20 24 28 32 36 40 44 48

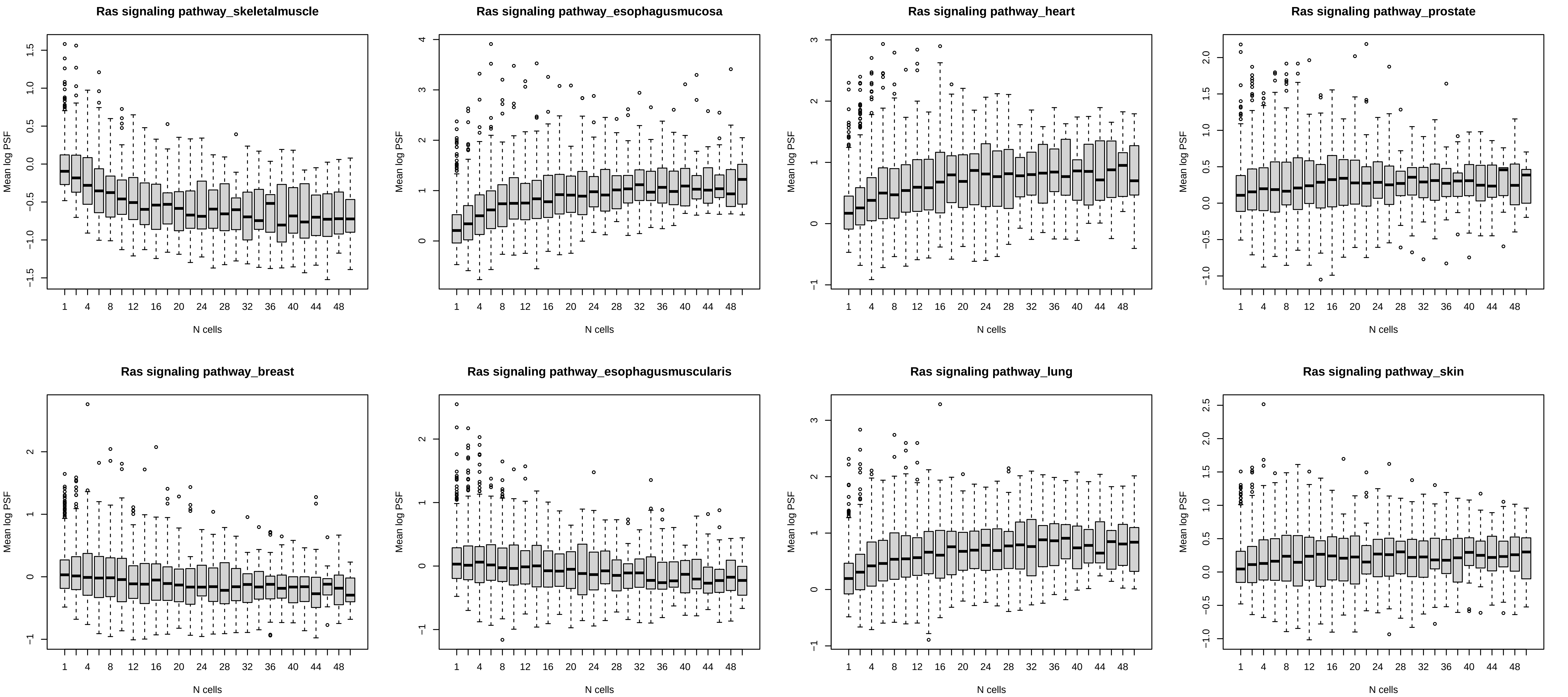
Ras signaling pathway_skeletalmuscle
Ras signaling pathway_esophagusmucosa
Ras signaling pathway_heart
Ras signaling pathway_prostate
Ras signaling pathway_breast
Ras signaling pathway_esophagusmuscularis
Ras signaling pathway_lung
Ras signaling pathway_skin
Mean log PSF
N cells

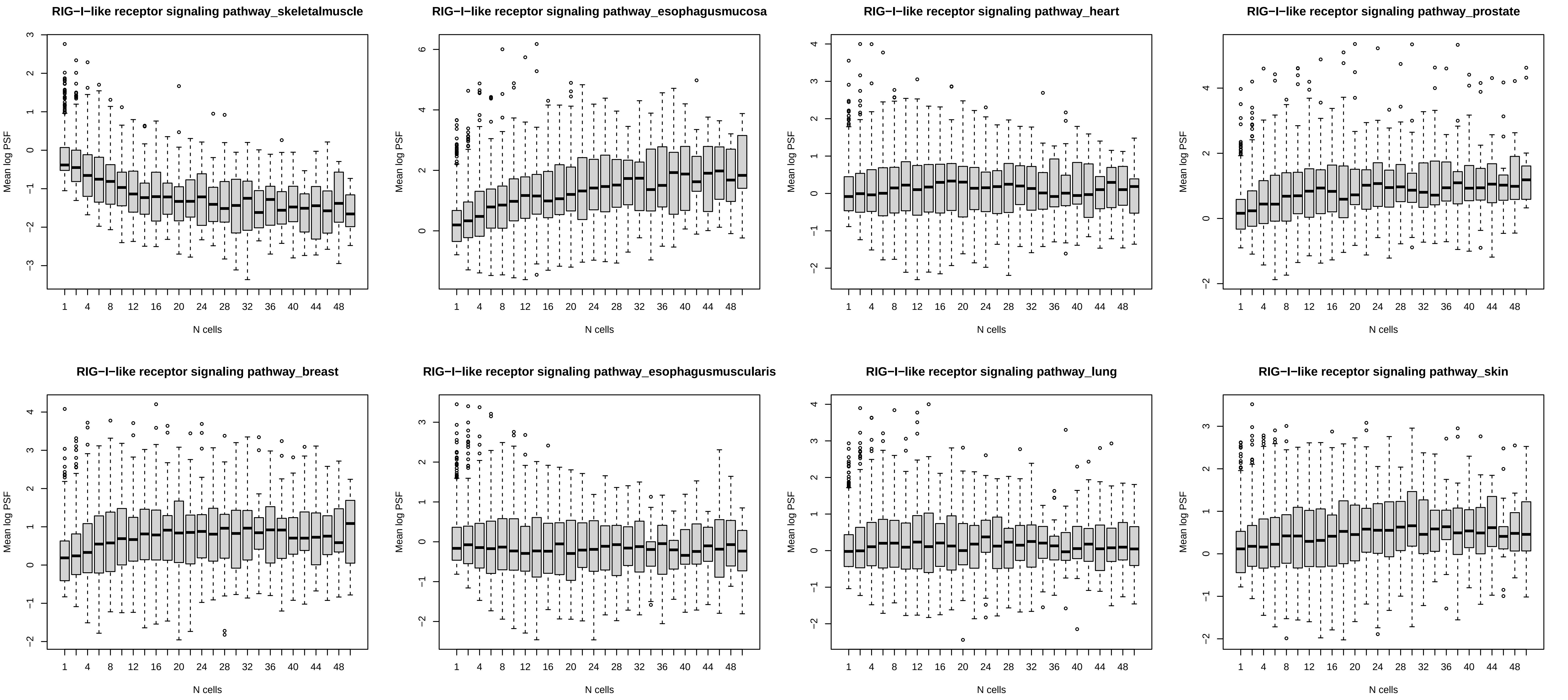
RIG−I−like receptor signaling pathway_skeletalmuscle
RIG−I−like receptor signaling pathway_esophagusmucosa
RIG−I−like receptor signaling pathway_heart
RIG−I−like receptor signaling pathway_prostate
RIG−I−like receptor signaling pathway_breast
RIG−I−like receptor signaling pathway_esophagusmuscularis
RIG−I−like receptor signaling pathway_lung
RIG−I−like receptor signaling pathway_skin
Mean log PSF
N cells
1 4 8 12 16 20 24 28 32 36 40 44 48

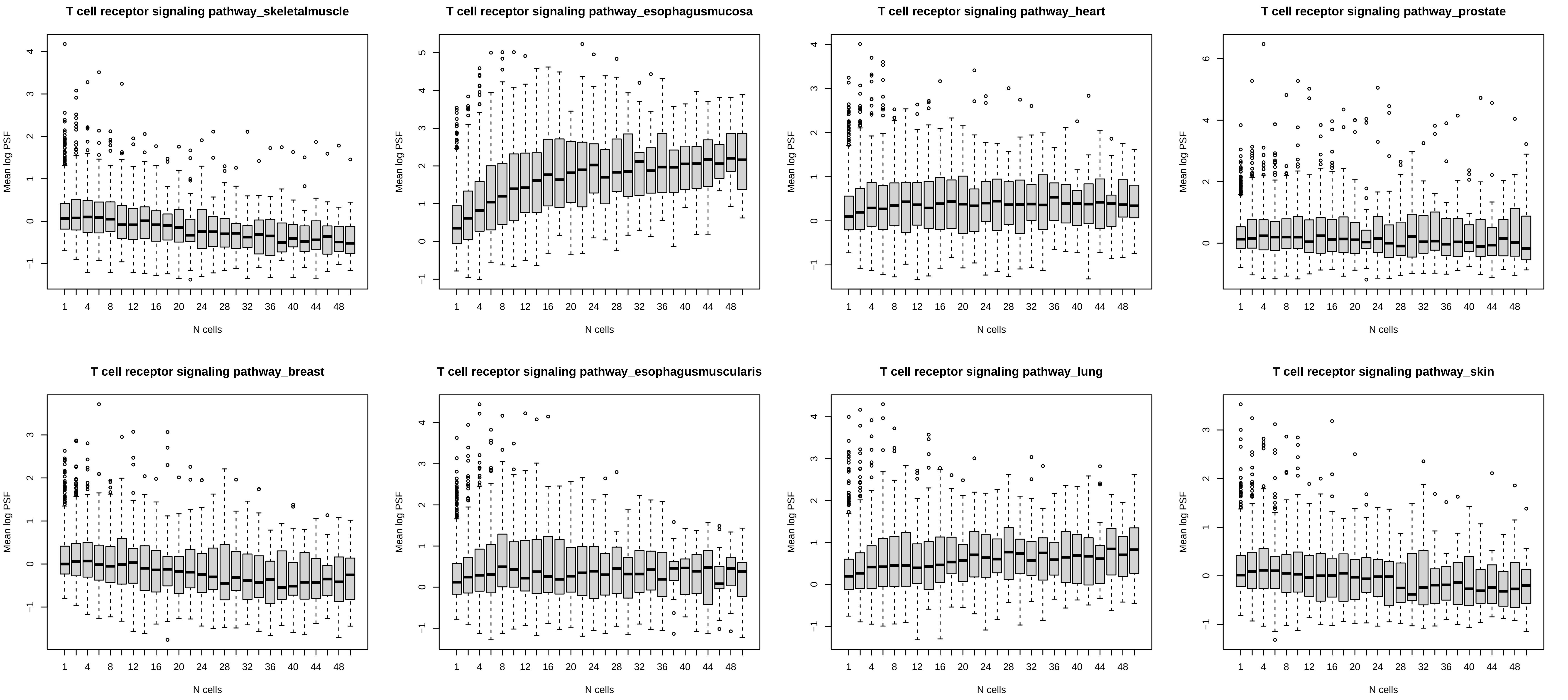
T cell receptor signaling pathway_skeletalmuscle
T cell receptor signaling pathway_esophagusmucosa
T cell receptor signaling pathway_heart
T cell receptor signaling pathway_prostate
T cell receptor signaling pathway_breast
T cell receptor signaling pathway_esophagusmuscularis
T cell receptor signaling pathway_lung
T cell receptor signaling pathway_skin
Mean log PSF
N cells
1 4 8 12 16 20 24 28 32 36 40 44 48

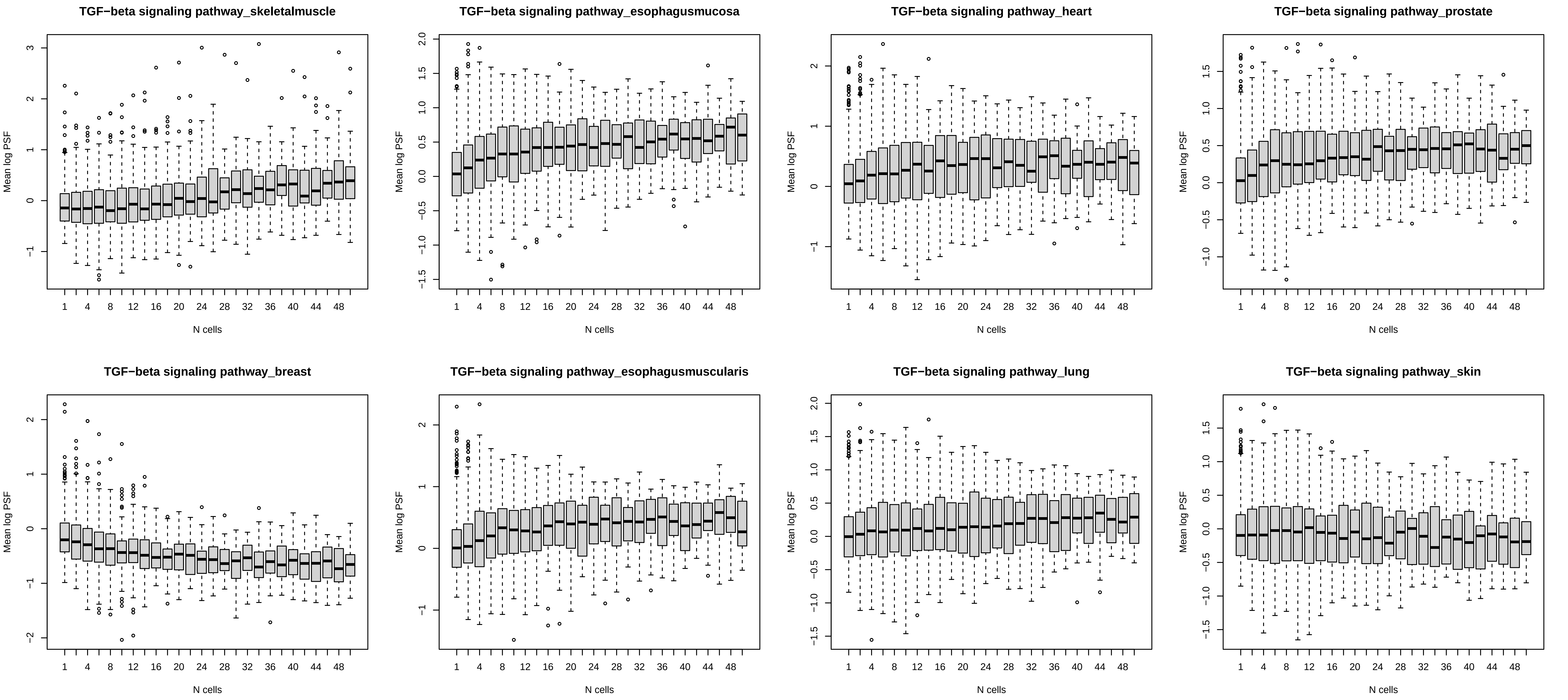
TGF−beta signaling pathway_skeletalmuscle
TGF−beta signaling pathway_esophagusmucosa
TGF−beta signaling pathway_heart
TGF−beta signaling pathway_prostate
TGF−beta signaling pathway_breast
TGF−beta signaling pathway_esophagusmuscularis
TGF−beta signaling pathway_lung
TGF−beta signaling pathway_skin
Mean log PSF
N cells

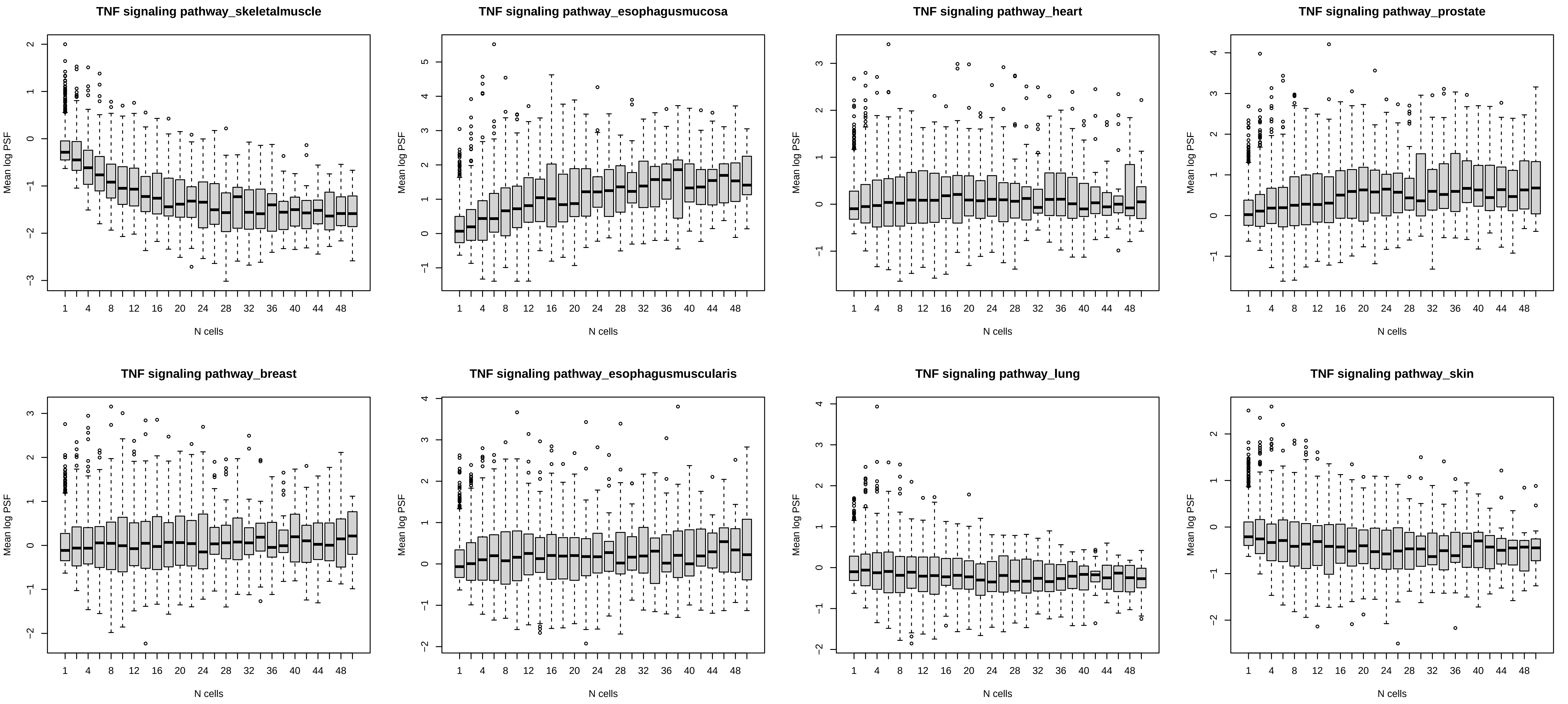
TNF signaling pathway_skeletalmuscle
TNF signaling pathway_esophagusmucosa
TNF signaling pathway_heart
TNF signaling pathway_prostate
TNF signaling pathway_breast
TNF signaling pathway_esophagusmuscularis
TNF signaling pathway_lung
TNF signaling pathway_skin
Mean log PSF
N cells

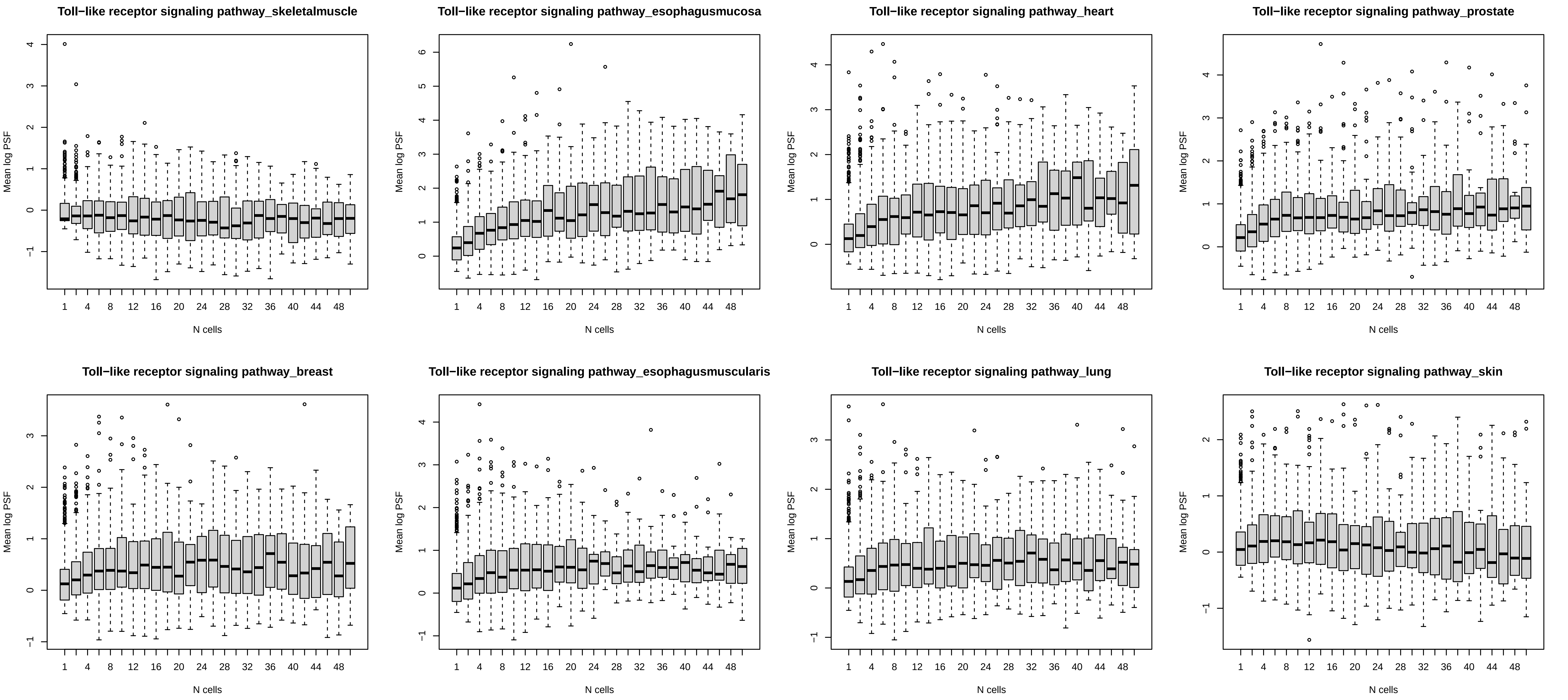
Toll−like receptor signaling pathway_skeletalmuscle
Toll−like receptor signaling pathway_esophagusmucosa
Toll−like receptor signaling pathway_heart
Toll−like receptor signaling pathway_prostate
Toll−like receptor signaling pathway_breast
Toll−like receptor signaling pathway_esophagusmuscularis
Toll−like receptor signaling pathway_lung
Toll−like receptor signaling pathway_skin
Mean log PSF
N cells
1 4 8 12 16 20 24 28 32 36 40 44 48

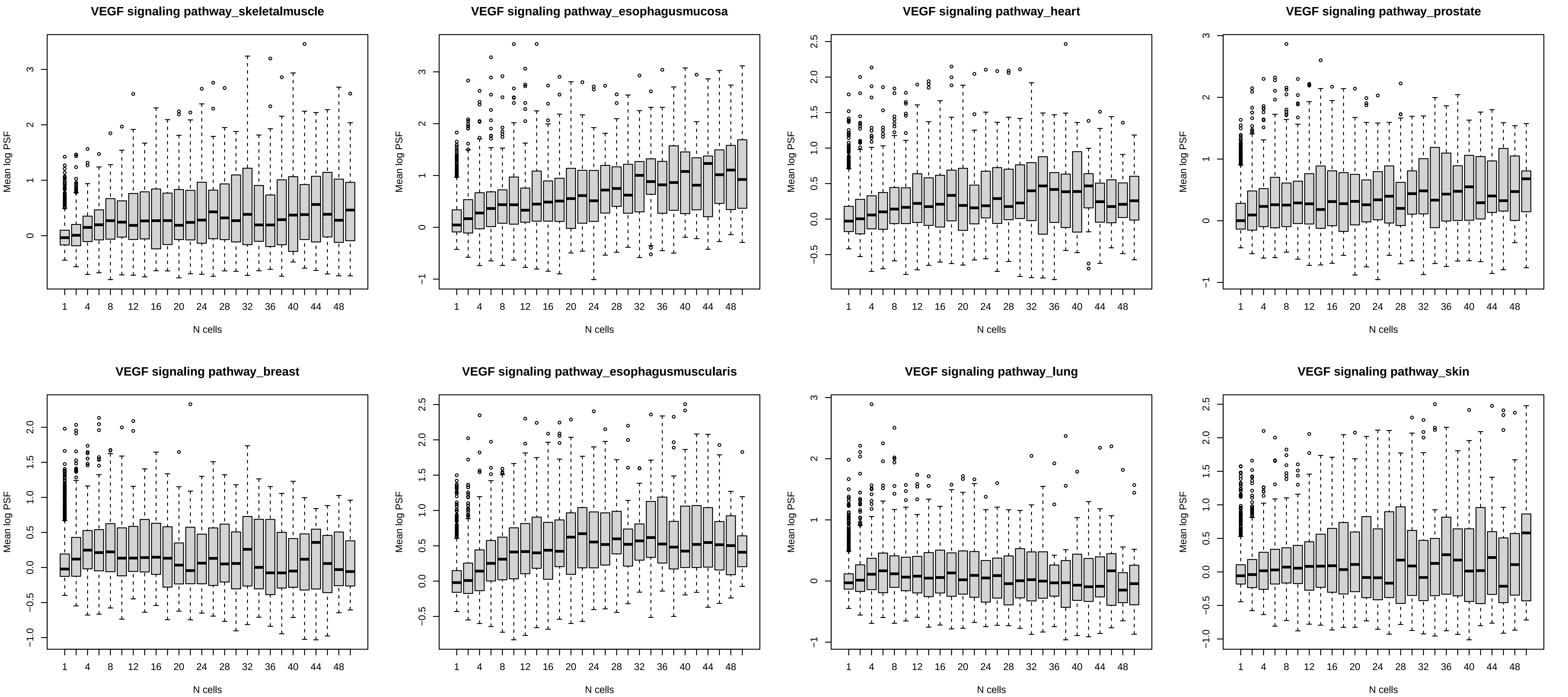
VEGF signaling pathway_skeletalmuscle
VEGF signaling pathway_esophagusmucosa
VEGF signaling pathway_heart
VEGF signaling pathway_prostate
VEGF signaling pathway_breast
VEGF signaling pathway_esophagusmuscularis
VEGF signaling pathway_lung
VEGF signaling pathway_skin
Mean log PSF
N cells
1 4 8 12 16 20 24 28 32 36 40 44 48

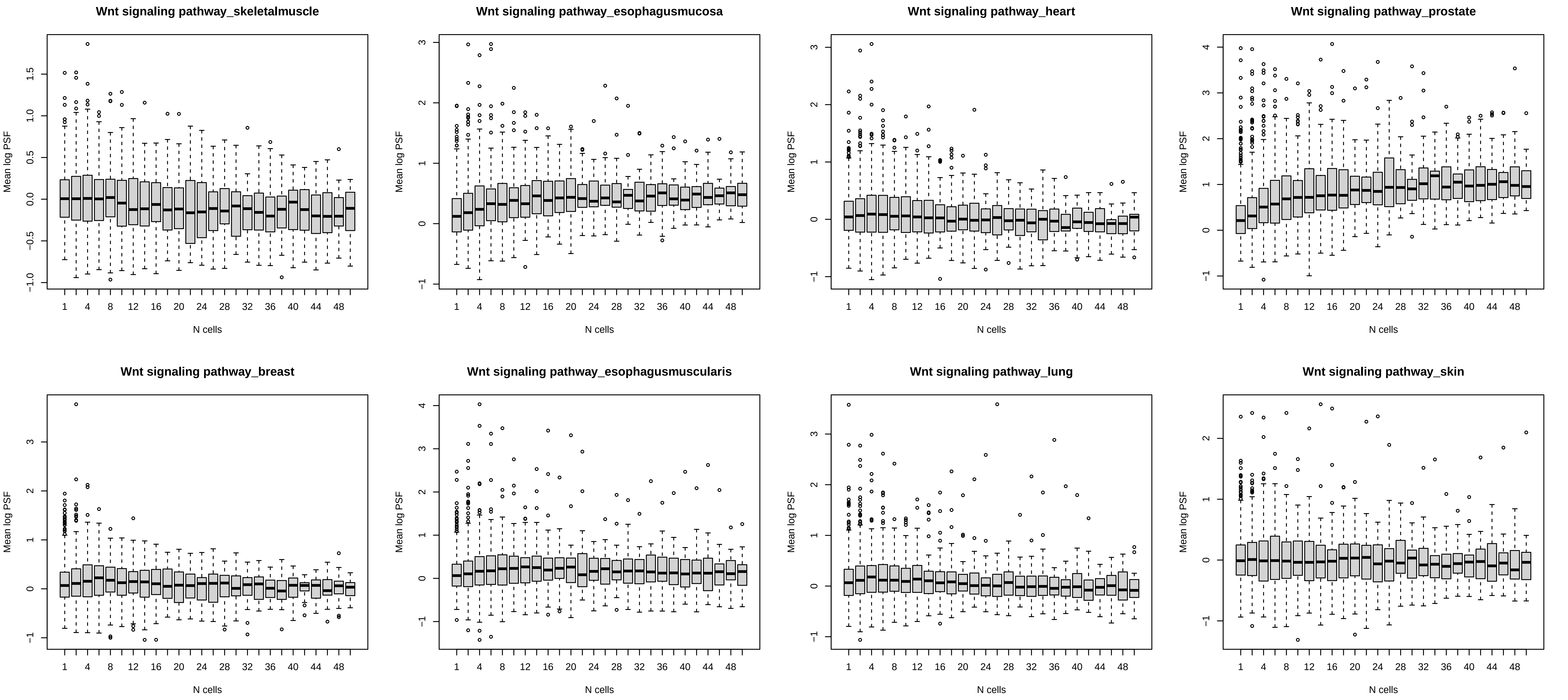
Wnt signaling pathway_skeletalmuscle
Wnt signaling pathway_esophagusmucosa
Wnt signaling pathway_heart
Wnt signaling pathway_prostate
Wnt signaling pathway_breast
Wnt signaling pathway_esophagusmuscularis
Wnt signaling pathway_lung
Wnt signaling pathway_skin
Mean log PSF
N cells
1 4 8 12 16 20 24 28 32 36 40 44 48
